# Scoping review of wearable device data evaluated in clinical studies

**DOI:** 10.1136/bmjopen-2025-111027

**Published:** 2026-11-02

**Authors:** Emer Gunne, Saber Azami-Aghdash, Sinead Holden, Peter Doran

**Affiliations:** 1 School of Medicine, University College Dublin, Dublin, Ireland; 2 Tabriz Health Services Management Research Center, Health Management and Safety Promotion Research Institute, Tabriz, Iran (the Islamic Republic of); 3 University College Dublin, Dublin, Ireland

**Keywords:** Digital Technology, Wearable Electronic Devices, Machine Learning, Clinical Trial

## Abstract

**Abstract:**

**Background:**

Wearable device data are increasingly used to provide physiological and behavioural measures in clinical studies.

**Aims:**

This scoping review maps out the data collected by wearable devices from participants in clinical studies, including the approaches used to interpret this data.

**Methods:**

Scopus, Web of Science, the Cochrane Library and PubMed, along with ClinicalTrials.gov, were searched using relevant keywords. Clinical studies published or registered from January 2020 to July 2026 inclusive, in which wearable devices were used to monitor participants, were sought. Data on disease demographics, sample size, gender, ethnicity, study design, wearable type, application and data interpretation approaches were extracted using a data extraction form within Covidence software and then exported and compiled for analysis.

**Results:**

Out of 13 744 identified records, 147 original articles were included. Cardiovascular diseases, followed closely by neurological diseases, were the most frequently studied clinical indications. The median sample size was 85 (range 1–455 699). Overall, the most measured wearable data were physical activity (106 (72%)), heart rate (48 (33%)) and sleep (34 (22%)). 98 studies used wearable data to measure outcomes (52 primary and 46 secondary), and 49 studies used wearable data for disease monitoring. 27 studies applied advanced computational or machine learning methods, with 9 studies integrating multiple wearable-derived variables to develop composite or disease-specific digital biomarkers.

**Conclusions:**

The studies included in this review report the use of wearables to provide continuous, real-world data on patients’ physiological and behavioural metrics to assess health outcomes and disease progression outside traditional clinical settings. Advanced computational approaches remain a minority, with most studies relying on device-derived measures and conventional analytical methods.

**STRENGTHS AND LIMITATIONS OF THIS STUDY**

- The Arksey and O’Malley framework was used to conduct the study, with adherence to the Preferred Reporting Items for Systematic reviews and Meta-Analyses extension for Scoping Reviews (PRISMA-ScR) reporting guidelines to ensure transparency and reproducibility.
- A comprehensive search strategy was applied across Scopus, Web of Science, the Cochrane Library and PubMed, with ClinicalTrials.gov also searched to identify relevant clinical studies.
- A structured data charting approach was used to systematically characterise wearable devices, measured data, clinical applications and analytical approaches across included studies.
- Consistent with a scoping review methodology, formal quality appraisal and risk-of-bias assessment were not performed, limiting the ability to comment on the strength of the evidence.
- The review was restricted to English-language, peer-reviewed publications, which may have resulted in relevant studies being excluded.

## Introduction

 Advances in digital health technologies have facilitated decentralised clinical trials, allowing study participants to be managed using electronic tools for remote onboarding and consenting, along with wearables for home monitoring. The Pfizer REMOTE trial (2014)^1^ was the first-ever randomised virtual clinical trial. Another study to highlight the role of digital health technologies in reducing the costs of clinical trials by facilitating decentralised and hybrid trial designs was the REACT-AF trial, which used Apple Watches to remotely monitor atrial fibrillation (AF), eliminating the need for in-person follow-up visits and thereby reducing participant burden and trial expenses.^2–4^

A wearable device is an electronic device containing sensors that is worn to collect, process and communicate data from the wearer.^5^ The evolution of wearable devices over the past two decades has seen an expansion in their capabilities. Following the successful introduction of the smartwatch in 2015,^6^ we now have sophisticated multisensor wearable devices integrating a wide range of advanced features.^7^ As sensor technology has matured, wearables have evolved to include medical-grade devices with wider clinical applications such as integrated irregular heart rate (HR) algorithms to identify AF,^8^ wearable ECG measurement^9^ and blood oxygen (SpO_2_) sensors with algorithms for sleep apnoea detection.^10 11^

Wearable devices have facilitated the shift to decentralised clinical studies, enabling the provision of continuous, real-world monitoring of patients’ physiological and behavioural metrics, facilitating more accurate, remote and patient-centric assessment of health outcomes and disease progression outside traditional hospital clinical settings.

A recent review by Miyakoshi and Ito^12^ reported that the number of clinical studies registered on ClinicalTrials.gov incorporating wearable devices has increased since 2012; however, absolute numbers or percentages were not reported. An exploratory search of ClinicalTrials.gov using the term ‘wearable device’ and filtering by ‘First Posted Date’ reveals the following counts: 2012 (n=10), 2019 (n=154) and 2023 (n=273). While this search does not statistically demonstrate a growth in the total number of trials involving wearable technology, it suggests an upward trend.

The V3 framework^13^ (Verification, Analytical Validation and Clinical Validation) is a model used to evaluate digital health technologies like wearables to ensure they function correctly, measure accurately and produce clinically meaningful data. This is essential for regulatory acceptance and clinical integration of digital biomarkers.

The Food and Drug Administration (FDA) recently released its final guidance for using digital health technology for remote data acquisition in clinical investigations.^14^ It is expected that as the regulatory environment evolves and wearable devices progress through approval pathways with the relevant regulatory bodies (US FDA and the European Medicines Agency and Notified Bodies), there will be increasing opportunities to remotely monitor physiological data of clinical populations in clinical studies.^15–17^

This scoping review is novel in that it integrates evidence from both interventional and observational clinical research studies to provide a comprehensive overview of wearable device data use, including the disease areas most investigated, the physiological and behavioural variables most frequently captured, the use of wearable data as clinical outcomes or monitoring tools and the adoption of advanced computational or machine learning (ML) approaches for data analysis.

## Methods

The Arksey and O’Malley^18^ methodological framework for scoping studies was used in conducting this review, in addition to the Preferred Reporting Items for Systematic Reviews and Meta-Analyses extension for Scoping Reviews (PRISMA-ScR)^19^ statement for reporting (online supplemental file 1). It is a six-stage framework designed to achieve in-depth and comprehensive results following an iterative process to ensure a thorough approach to the literature. The framework’s six stages consist of: (a) defining the research question, (b) identifying relevant studies or a search strategy, (c) selecting studies, (d) charting the data, (e) collating, summarising and reporting the data and (f) an optional consultation stage.^18^

### Identifying the research question

The main research question of this current study is: ‘what are the current approaches to the collection and analysis of data extracted from wearable electronic devices used in clinical research studies?’

We used the ‘PCC’ framework^20^ (Population, Concept and Context) to define the research question:

- Population: patients participating in clinical research studies in which wearable electronic devices were used to monitor their disease status.
- Concept: physiological parameters are measurable factors that reflect the normal or pathological function of a biological system, such as HR, oxygen saturation, respiratory rate (RR), blood pressure and ECG and behavioural parameters include activity levels and sleep.
- Context: there were no restrictions on context, but most of the reviewed clinical studies focused on the use of wearable electronic devices in patients’ natural living environments—at home.

This study was designed as a scoping review, with the primary objective of mapping existing literature on the collection and analysis of data derived from wearable electronic devices in clinical studies, including the types of devices used, clinical populations studied, data collection approaches and analytic methods applied.

### Information sources

An experienced University Hospital librarian was consulted in the preparation of the present study’s search strategy. The search strategy comprised both MeSH (Medical Subject Headings) and keyword search terms (online supplemental file 2). The search was completed on PubMed, Web of Science, the Cochrane Library and Scopus databases. We searched ClinicalTrials.gov to identify potentially eligible registered clinical research studies. A decision was made to narrow the search to trials from January 2020 to July 2026 to build on the broader and more heterogeneous review by Huhn *et al* up to the year 2020.^21^ The search date range enabled a more focused and clinically relevant analysis considering the rapid evolution, growing accessibility and increasing maturity of wearable technologies and advanced computational or ML approaches in recent years.

### Study selection

Clinical research studies in which wearable devices were used to collect data from participants were included in the review. The process for selecting sources of evidence was carried out by two authors according to the following eligibility criteria.

Inclusion criteria are outlined below:

1. Type of study: clinical study. Defined as a work that reports on the results of a research study to evaluate interventions or exposures on biomedical or health-related outcomes.^22^ The two main types of clinical research studies are interventional studies (clinical trials) and observational studies.^22^ In a clinical trial, participants may receive diagnostic, therapeutic or other types of interventions, and assignments are determined by the protocol. An observational study is defined as a work that reports on the results of a clinical study in which participants may receive diagnostic, therapeutic or other types of interventions, but the investigator does not assign participants to specific interventions (as in an interventional study).^22^
2. Publication date: last 7 years (from January 2020 to July 2026 inclusive).
3. Wearable devices used to measure a physiological parameter. For this scoping review, we define a wearable device as an electronic device containing sensors that is worn by the study participant.^9^ A physiological parameter is a measurable factor that reflects the normal or pathological function of a biological system.^10^ Physiological parameters include but are not limited to HR, oxygen saturation, RR, blood pressure and ECG. Behaviour parameters include activity levels and sleep.
4. Application of the wearable: wearable device data are used as a component of a study outcome, or the wearable data are used for disease monitoring.

Exclusion criteria are outlined below:

1. General publication exclusions: non-English language publications, articles limited to abstracts, posters, conference presentations, viewpoints, perspective-based articles, reviews or guidelines or publications of modifications to previous protocols.
2. Study design exclusions: survey studies, qualitative studies only assessing attitudes towards wearables without collecting data and cross-sectional studies.
3. Population exclusions: studies conducted solely in healthy populations without a clinical condition, intervention or clinically relevant outcome. Population-based cohorts were eligible when wearable-derived physiological or behavioural data were evaluated for disease detection, monitoring or clinically relevant responses to a healthcare intervention.
4. Study type exclusions: validation studies (prototype or usability-focused) or device comparative studies.
5. Wearable technology exclusions: wearables not used for specified periods or continuous monitoring. For these studies, we interpreted continuous monitoring as the process of collecting physiological or behavioural data from a participant in near real time using a wearable device that is worn throughout daily activities and sleep, except during necessary interruptions such as charging or bathing. Also, devices that did not measure a physiological or behavioural parameter and studies that did not involve wearables or lacked sufficient information regarding the physiological or behavioural data collected and its use.
6. Intervention and application exclusions: studies focused solely on gait analysis and studies using continuous glucose monitors exclusively.

All identified articles were imported into Covidence review software, where duplicates were automatically removed. The EndNote reference manager was used to both automatically and manually remove duplicates and studies whose design met the exclusion criteria. The remaining studies were eligible for title and abstract screening.

Title and abstract screening were conducted in EndNote by the first author. A full-text review was subsequently carried out in Covidence review software by this author. The PRISMA 2020^19^ flow diagram, illustrating the flow of studies through the different stages of the review was compiled to report the selection and screening process results.^16^

### Charting the data

The Extraction 2 Tool in Covidence, designed for scoping reviews, was used for data extraction. A customisable data extraction template (online supplemental file 3) was developed, informed by the scoping review objectives and in line with the PRISMA-ScR reporting guidelines. The template was refined throughout the extraction process, with all changes made to the template being promoted to every study included, making sure consistent data were collected across all studies. The first author carried out data extraction while the second author double-checked the process. Covidence guided the reviewer through the process, and studies were displayed one at a time with clear prompts to guide users through the required extraction fields. Once all studies had been extracted, the Covidence export-to-file facility was used to export the results data to Excel for analysis.

### Data analysis

A descriptive synthesis approach was employed to summarise and characterise the included studies. This approach aimed to systematically organise and present key features of the literature rather than generate or interpret qualitative themes. The first stage involved an initial familiarisation with the papers included; secondly, identifying and extracting predefined data items based on the data extraction template; thirdly, tabulating and grouping extracted information into structured categories to enable comparison across studies and finally, reviewing the results of each element. Descriptive statistics, such as frequency, percentage, mean and median, were used to report quantitative data. Data analysis was performed by the first author, while the second author cross-checked the analysis using the Covidence data extraction export file opened in Microsoft Excel (2016). Where data items were not reported in the included studies, these were recorded as ‘not reported’ within the data extraction template and excluded from the relevant summaries where appropriate. Each element of the extraction template used to characterise the studies was discussed by the team to prepare the results for reporting. The primary outcomes of interest were descriptive in nature and included study characteristics (geographical distribution, sample size and study design), participant demographics (gender and ethnicity), clinical populations, types and applications of wearable devices and data collection and analytical approaches.

### Patient and public involvement

None.

## Results

### Results of screening

A total of 13 744 studies were identified through our database search, as depicted in the PRISMA flowchart in figure 1. A Covidence export of 11 143 articles was subsequently opened in the EndNote reference management tool, where a further 717 duplicates were removed automatically, and 807 duplicates, 24 non-English studies, 14 laboratory-only studies, 34 survey studies, 180 qualitative-only studies, 424 reviews, 75 cross-sectional studies and 38 perspectives were manually eliminated. 2448 studies went through title and abstract screening; 358 studies were included for full-text review, resulting in 211 studies being excluded based on the criteria listed in the PRISMA flow diagram (figure 1). Finally, 147 studies were included in the scoping review^23–169^ (online supplemental file 3, tables S1-S4).

**Figure 1:**
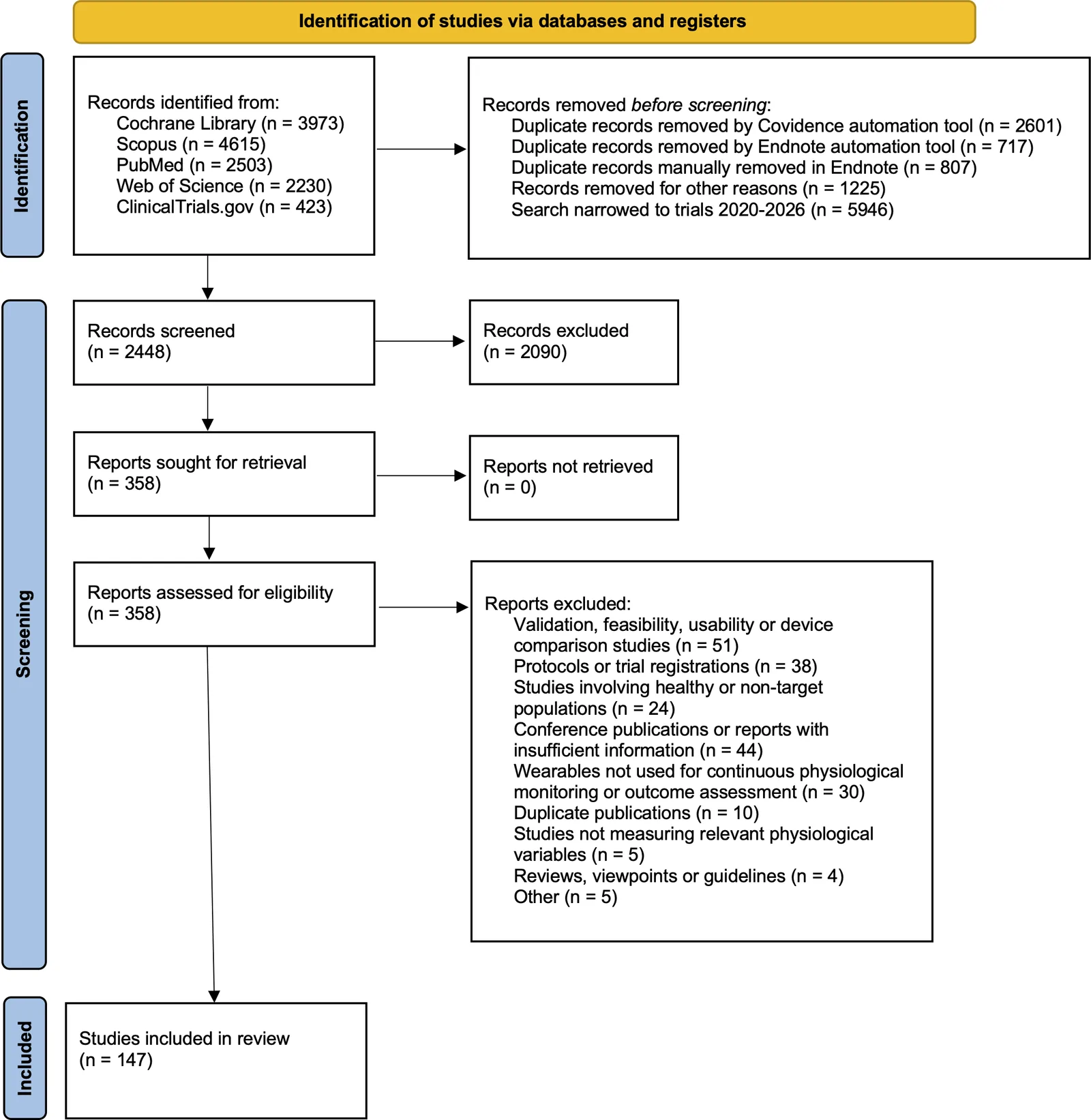
Preferred Reporting Items for Systematic Reviews and Meta-Analyses (PRISMA) 2020 flow diagram for new reviews that included searches of databases and registers only.

### Characteristics of studies

Of the 147 studies extracted and included in the review analysis, 78 (53%) were randomised controlled trials (table 1). Study duration ranged from 1 day to 1095 days, with a median of 84 days’ duration. The median number of participants per study was 85 (range 1–4 55 699), with a combined total of 626 572. Participant demographic characteristics were inconsistently reported across studies. Among the 112 studies reporting participant sex, 394 901/605 397 (65.2%) participants were female. This was strongly influenced by the largest study, reported by Lubitz *et al*^50^, in which 322 131/455 699 (70.7%) participants were female; excluding this study, 72 770/149 698 (48.6%) participants were female. Participants’ ages ranged from 4 to 96 years, reflecting the diverse clinical populations included in the review. Race and ethnicity were less consistently reported, and reporting categories varied considerably between studies, precluding meaningful aggregation across the included populations.

**Table 1:** Characteristics of studies

Study characteristics	Studies (N=147), n (%)	Participants (N=626 572), n (%)
Year of publication		
2020	6 (4.1)	341 (0.1)
2021	7 (4.8)	26 856 (4.3)
2022	7 (4.8)	457 580 (73)
2023	17 (11.6)	106 935 (17.1)
2024	37 (25.2)	6449 (1.0)
2025	41 (27.9)	24 340 (3.9)
2026	32 (21.8)	4071 (0.6)
Continents		
North America	51 (34.7)	468 668 (74.8)
Europe	55 (37.4)	131 435 (21)
Asia	29 (19.7)	25 232 (4)
Oceania	9 (6.1)	685 (0.1)
Multicontinental	3	552 (0.09)
Study design		
Observational studies	58 (39.5)	142 026 (22.7)
Randomised controlled trial	78 (53.1)	28 315 (4.5)
Non-randomised interventional studies	11 (7.5)	456 231 (72.8)

N, total number of studies or participants; n (%), number and percentage within each category.

Among studies for which the wear pattern could be determined (n=101), 46 (45.5%) were continuous wear (24 hours) throughout the study. Other studies reported using wearables for specified periods of wear, such as night-time only to monitor sleep or Monday–Friday, 07:00 to 21:00, for step count.

#### Geographical distribution of studies

Europe performed the greatest number of studies (n=55, 37.4%), including the second largest study by SchalKamp *et al,*^59^ which had 103 712 participants. 51 (34.7%) of the studies took place in North America, including the largest study,^50^ with 455 699 participants. Most studies were conducted in high-income countries (127/147, 86.4%), with limited representation from middle-income countries and no studies conducted exclusively in low-income countries.

#### Clinical population studied

Cardiovascular diseases and neurological diseases were the most common diseases studied, followed closely by oncology, with n=33, n=32 and n=31 studies, respectively. A further analysis within the disease category quantified the number of studies conducted, identifying 14 (9.5%) on rare diseases, including patients with the following rare diseases: pachyonychia congenita,^49^ pancreatic neuroendocrine tumour,^28^ amyotrophic lateral sclerosis^37^ and Barth syndrome^63^ (figure 2).

**Figure 2:**
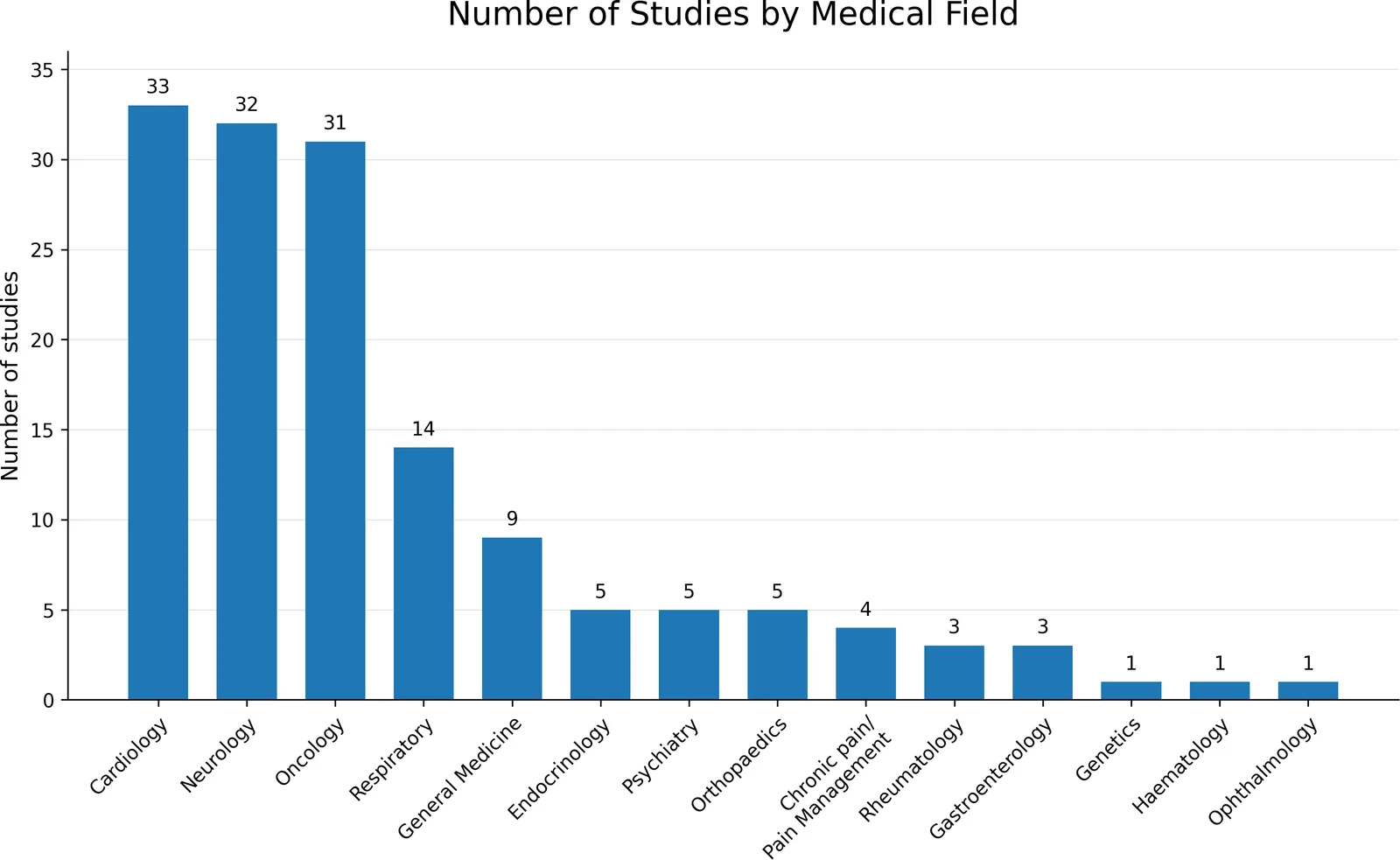
Medical field of clinical studies employing wearable devices to monitor participants (n=147).

#### Wearable device categories used in clinical studies

All studies report the type of wearable device used. Wrist-worn consumer-grade devices were the predominant (51%) wearable devices used in the studies. The most popular devices used were Fitbit (41, 28%), ActiGraph (16, 11%), Garmin (11, 7%), Apple Watch (8, 5%) and Withings (5, 3%). We have categorised the type of wearable used in the study and report their frequency as follows in table 2.

**Table 2:** Wearable device category

Wearable category	Number of studies (%)	Description	Examples
Consumer-grade wearable	75 (51)	Commercial-grade sensors for biometric data; often include smartwatch features like notifications and mobile integration.	Fitbit, Garmin, Apple Watch, Huawei, Withings
Research-grade activity or biometric monitor	55 (37.4)	Reliable sensors for validated physical activity and physiological data, used in research settings.	Actiwatch, Axivity AX3, GENEActiv, Empatica E4
Medical-grade biometric monitor	17 (11.6)	FDA-cleared devices for clinically accurate biometric monitoring suitable for patient care.	Biobeat Watch, VitalPatch, AVIVO, LifeVest, Medtronic SEEQ, Zephyr BioPatch, Bittium ECG, Biovotion Everion

FDA, Food and Drug Administration.

#### Application of wearable data

Overall, the most measured wearable data were physical activity (106/147 (72%)), HR (48/147 (33%)) and sleep (34/147 (23%)) (online supplemental file 4).

Outcome assessment was the most common application of wearable device data used by 98 (67%) of the studies. 52 (35%) of the studies used wearable data as a component of primary outcomes, while 46 (31%) used it as a component of secondary outcomes. For example, Chen *et al*^26^ used a Verily’s smartwatch to assess whether digital measurements (activity levels and sleep) could detect treatment effects in a substudy of the Phase 2 trial of mevidalen for Lewy body dementia. This study concluded that digital measurements detected treatment effects in a smaller cohort over a shorter period than conventional clinical assessments.

Lim *et al*^47^ used a GENEActiv, a wrist-worn accelerometer, to identify the impact of medicines with anticholinergic or sedative properties on sedentary time. Secondary outcomes: measured change in 24-hour activity intensity, sleep time and total activity time at baseline, 6 months post-baseline and 12 months post-baseline.

Gepner *et al*^34^ used a Biobeat chest patch sensor to perform objective continuous assessment of HR, HR variability, SpO_2_, RR and skin temperature post-BNT162b2 mRNA COVID-19 vaccination to assess reactions.

Umbricht *et al*^62^ used a GENEActiv actigraphy device to develop a human activity recognition model to assess patient activity in relation to negative symptoms of schizophrenia. Findings support the use of wrist-worn devices to derive activity-based and gesture-based digital outcome measures for patients with schizophrenia with negative symptoms in a clinical trial setting.

Disease monitoring was the second most common application of wearable data (49, 33%); Gashi *et al*^33^ used the Biovotion Everion wearable device to investigate whether data from the wearable and a smartphone could be used for monitoring different aspects of multiple sclerosis (MS) disease. Data were used to distinguish patients with MS from controls and to classify MS type.

Levi *et al*^45^ used a Garmin vivosmart 4 smartwatch and an application to predict and detect side-effect severity following vaccinations.

Activity data were most frequently captured in cardiovascular, neurological, oncology and psychiatric populations being measured in 20 of 33 cardiovascular studies, 25 of 32 neurological studies, 24 of 31 oncology studies and all 5 psychiatric studies. HR data were captured in 11 cardiovascular, 10 neurological, 8 oncology and 7 respiratory studies. Sleep data were most frequently captured in neurological studies (n=12), followed by oncology (n=5) and cardiovascular studies (n=1).

#### Analytical approaches

A range of analytical approaches were applied to wearable-derived data, with individual studies frequently using more than one method. Association and regression methods were the most used approaches (n=75, 51%), followed by descriptive or comparative statistical analyses (n=72, 49%) (table 3). Longitudinal approaches, including mixed-effects, repeated-measures and generalised estimating equation models, were used in 52 studies (35.4%). Survival or other time-to-event methods were used in 17 studies (11.6%), while 7 studies (4.8%) applied time-series or signal-level analytical approaches. Advanced computational approaches, including ML, classification, prediction and clustering methods, were identified in 27 studies (18.4%) (tables 4 and 5). These approaches included random forest, gradient boosting, support vector machines, clustering and neural network or deep-learning models.

**Table 3:** Summary of analytical approaches used by clinical studies

Analytical approach	Studies, n (%)	Examples
Association/regression	75 (51.0)	Correlation, linear and logistic regression, multivariable regression
Descriptive/comparative statistics	72 (49.0)	Descriptive statistics, t-tests, non-parametric tests, group comparisons
Longitudinal/repeated-measures analysis	52 (35.4)	Mixed-effects models, GEE, repeated-measures models
Survival/time-to-event analysis	17 (11.6)	Cox regression, Kaplan-Meier/time-to-event methods
Time-series/signal-level analysis	7 (4.8)	Temporal patterns, signal-level or multidimensional time-series analysis
Advanced computational/ML approaches	27 (18.4)	Classification, prediction, clustering, random forest, boosting, neural/deep learning

GEE, generalised estimating equations; ML, machine learning.

**Table 4:** Advanced computational and ML applications of wearable-derived data

Study	Clinical population	Wearable device	Wearable-derived information	Clinical translation/analytical application
Friesner *et al*^30^	Cancer undergoing chemoradiation	Garmin	Activity/step-derived features	Prediction of unplanned hospitalisation
Garland *et al*^32^	Chronic pain	Garmin vivosmart	HR/HRV-derived measures	Physiological pattern assessment during mindfulness intervention
Kimura *et al*^42^	MCI	Silmee W20	Physiological/behavioural wearable features	Prediction of amyloid positivity
Levi *et al*^45^	Vaccinated adults	Garmin vivosmart 4	Physiological/activity features	Prediction of vaccine side-effect severity
Patel *et al*^54^	Recently hospitalised patients	Withings Steel	Activity-derived features	Prediction of 30-day hospital readmission/outcome
Risch *et al*^56^	COVID-19	Mi Band	Longitudinal physiological features	Presymptomatic infection detection using recurrent neural networks
Schalkamp *et al*^59^	Parkinson’s disease	UK Biobank accelerometer	Activity and sleep features	Identification/risk stratification of prodromal Parkinson’s disease
Sindorf *et al*^60^	Stroke	ActiWatch Spectrum	Wearable activity/sleep-derived features	Classification/detection of sleep apnoea
Umbricht *et al*^62^	Schizophrenia	GENEActiv	Accelerometry-derived activity/gesture features	Activity and gesture classification
Van den Eynde *et al*^63^	Barth syndrome	AVIVO System	HR/RR time-series and derived temporal/spectral features	Identification of treatment-response phenotypes using clustering
Wood *et al*^66^	Cancer	Fitbit Charge HR	Activity and HR-derived measures	Modelling/prediction of physical function
Yang *et al*^69^	Cardiovascular disease	RockBand	Physical activity-derived features	Prediction of clinical recovery/deterioration
Gill *et al*^92^	AF	Fitbit Charge 2	Wearable HR/activity-derived information	Prediction of NYHA functional class using a self-supervised CNN
Wang *et al*^64^	AF	HUAWEI WATCH GT 2 Pro	PPG-derived features	ML-based AF detection and estimation of AF burden
van Vliet *et al*^122^	AF	Corsano CardioWatch 287-2	PPG/ECG-derived rhythm information	AI-based ambulatory AF detection and burden quantification
Alcoceba-Herrero *et al*^136^	COVID-19	Bakeey E66 smartwatch+pulse oximeter	Multimodal physiological measurements	Automated alert/risk classification
Barua *et al*^138^	Cardiovascular disease	Fitbit Inspire	Longitudinal wearable time-series	Identification of activity/behavioural patterns using k-means clustering
Jones *et al*^152^	Bronchiectasis	ActiGraph GT3X+/Polar OH1	Accelerometry/activity-intensity features	Random-forest classification of physical-activity intensity

AF, atrial fibrillation; AI, artificial intelligence; CNN, convolutional neural network; HR, heart rate; HRV, heart rate variability; MCI, mild cognitive impairment; ML, machine learning; NYHA, New York Heart Association; PPG, photoplethysmography; RR, respiratory rate.

**Table 5:** Composite disease-specific digital biomarkers derived from wearable data

Study	Clinical population	Wearable device	Wearable-derived information	Clinical translation/analytical application
Creagh *et al*^27^	Rheumatoid arthritis	Apple Watch	Multimodal sensor-derived features	Disease classification and assessment of RA severity
Gashi *et al*^33^	MS	Biovotion Everion	Multimodal physiological and activity features	MS classification and assessment of disability/fatigue
Gupta *et al*^37^	Amyotrophic lateral sclerosis	ActiGraph GT3X-T	Multilimbed accelerometry-derived features	Quantification of motor impairment and disease progression
Lønfeldt *et al*^44^	OCD	Empatica E4	Multimodal physiological features	Detection/classification of OCD events
Rykov *et al*^58^	MCI	Empatica E4	BVP/HRV, EDA, skin temperature; 106 derived features	Prediction of neuropsychological/cognitive scores
Xu *et al*^67^	MCI	Fitbit Charge 4	Activity, HR, sleep and other physiological features	Classification of MCI status
Yang *et al*^70^	End-stage cancer	Actigraphy XB40ACT	Activity, angle and spin time-series	Prediction of in-hospital death versus stable discharge using LSTM
Stein *et al*^79^	MG	Wearable device	SpO₂, pulse and steps+patient-reported measures	Prediction/early detection of MG deterioration using cross-validated ML
Chen *et al*^26^	COPD	HUAWEI SMARTWATCH	HRV, RR, SpO₂+cough-derived features	Multimodal COPD classification/screening

BVP, blood volume pulse; COPD, chronic obstructive pulmonary disease; EDA, electrodermal activity; HR, heart rate; HRV, heart rate variability; LSTM, long short-term memory; MCI, mild cognitive impairment; MG, myasthenia gravis; ML, machine learning; MS, multiple sclerosis; OCD, obsessive-compulsive disorder; RA, rheumatoid arthritis; RR, respiratory rate; SpO_2_, blood oxygen.

Studies combined wearable sensor data with advanced analytical approaches to enhance clinical prediction, patient monitoring and disease differentiation; nine studies integrated multiple wearable-derived variables into a composite score (table 5).

## Discussion

This study highlights the characteristics of research on wearable devices worn by participants in clinical studies. Key findings include the geographic distribution of studies (most carried out in high-income countries), the most common wearable measures collected and their application (activity 72%, HR 33% and outcome assessment 67%), the focus on certain disease groups (cardiology, neurology and oncology) and the advanced computational approaches employed.

A marked increase in eligible publications was observed over the review period, from 6–7 studies annually between 2020 and 2022 to 37–41 studies in 2024–2025; however, this reflects the number of studies meeting the review’s specific eligibility criteria rather than necessarily indicating an increase in the overall use of wearable technologies in clinical research, and 2026 represents only a partial year.

Unsurprisingly, physical activity, HR and sleep were the most frequently collected wearable variables, given consumer-grade wearables were the most common wearable device employed. The number of studies collecting SpO_2_ was 11 out of 147; 4 were COVID-19 related. The FDA issued draft guidance on pulse oximeters in January 2025, following ongoing concerns that they may be less accurate in individuals with darker skin pigmentation (February 2024).^10^ The Masimo W1 received FDA clearance (medical-grade wearable watch) to provide continuous SpO_2_ levels for at-home and clinical use (2023).^11^ Both Apple Watch (September 2024) and Samsung have had FDA approval as over-the-counter devices to assess the risk of sleep apnoea; Apple uses accelerometer sensor data while Samsung uses an SpO_2_ sensor.

Outcome measurement was the most widely used application of wearable data across the studies; it is encouraging to see these data points increasingly used as components of primary (52/98) and secondary (46/98) outcomes to monitor treatment results. The integration of wearable data into clinical research offers continuous, objective and real-time insights into participants’ physiological and behavioural responses to interventions that are not captured by traditional ‘snapshot in time’ clinical assessments.^3^ This trend reflects a growing recognition of the importance of continuous, real-time data in understanding health outcomes. Continuous objective data collected by wearables enables the detection of fluctuations and trends that could be missed by intermittent assessments. For example, Downey *et al*^170^ found that continuous monitoring of surgical patients identified patient deterioration earlier, thus facilitating timely intervention compared with patients with intermittent monitoring.

In terms of disease population, a common feature across cardiovascular disease (15/33) and oncology (20/31) studies was the use of wearables in rehabilitation and recovery, particularly to objectively quantify physical activity and functional recovery following cancer treatment, surgery or cardiovascular intervention, and to support remotely delivered exercise programmes. A notable finding from this study was the prominence of neurological diseases among the disease populations, using wearable data. Neurological disease was a frequently studied area, likely due to the potential of wearables to track key variables such as physical activity and HR. It is interesting to note the application of wearables in neuropsychiatric illness, where sleep data are being measured as a potential marker of disease. Sleep disturbances are (DSM-5 (Diagnostic and Statistical Manual of Mental Disorders, Fifth Edition)) diagnostic features for major depressive disorder, generalised anxiety disorder, post-traumatic stress disorder and bipolar disorder and are consistent features of psychoses and most neurodegenerative disorders.^171^

There is substantial opportunity to extend these technologies to understudied or complex conditions, particularly those with unpredictable clinical trajectories and limited therapeutic options. For example, interstitial lung disease remains under-represented despite its substantial symptom burden and poor prognosis. Other promising areas for expansion include autoimmune and inflammatory diseases (eg, lupus and vasculitis), infectious disease surveillance and rehabilitation medicine following illness or injury, where continuous real-world monitoring could enable earlier detection of clinical deterioration, facilitate remote monitoring and support more responsive and personalised rehabilitation or care pathways, ultimately improving patient outcomes in areas of unmet need.

Wearable devices generate large volumes of high-frequency data, offering a rich, continuous view of patients’ real-world health. This presents a unique opportunity to detect subtle trends, early deterioration or behavioural patterns that may not be captured during episodic clinic visits. However, the volume of wearable data also introduces significant challenges in data handling, storage, quality assurance and interpretation. (Izmailova^172^ Wright^173^) Data loss due to non-wear time, device syncing failures and firmware updates can impact dataset completeness and reliability. Additionally, proprietary algorithms and inconsistent data standards across device manufacturers hinder reproducibility and data harmonisation across studies. Despite these issues, ML methods are particularly well suited to extract meaningful patterns from wearable data. ML models can handle large, noisy, time-series datasets, detect non-linear relationships and support real-time risk stratification or patient monitoring.^13^ When combined with clinical variables or patient-reported outcomes, wearable data have the potential to enhance predictive power and offer new insights into disease trajectories, treatment responses and personalised care.

The statistical analysis methods employed in the studies we reviewed were predominantly traditional statistical approaches (120 out of 147). While these traditional methods remain the mainstay of analysis, advanced computational methods/ML methods have become popular in recent years (27 out of 147) for handling complex, high-dimensional and big data. Previous literature, Huhn *et al*^21^ reported low adoption (6.15%) of advanced techniques such as ML models. As confidence in the validity of artificial intelligence methods builds, incorporating these more sophisticated data processing strategies could greatly enhance the depth, speed and accuracy of research findings.

The increasing adoption of consumer-grade wearables such as Fitbit, Apple Watch and Garmin devices in clinical research offers major advantages in terms of scalability, cost-efficiency and participant engagement. These devices are widely used, allowing for Bring Your Own Device study designs that reduce hardware costs and support real-world deployment across diverse populations. This approach facilitates longitudinal monitoring of health behaviours and physiological signals in naturalistic settings, expanding access to remote care and decentralised trials.^174^ However, using commercial devices also poses data standardisation, reliability and privacy challenges. Sensor accuracy may vary between models, firmware updates can affect data integrity mid-study and proprietary algorithms can limit transparency and reproducibility.^174^ Inconsistent wear patterns and device compliance further complicate data completeness and interpretability. Despite these issues, with robust analytical validation and clear context-of-use, consumer wearables represent a promising avenue for scalable, patient-centred digital health research.

### Limitations

Only studies published in English were included, which may have introduced language bias.

The search was restricted to records from January 2020 to July 2026 to ensure relevance to rapidly evolving health technologies, although this may have excluded earlier foundational studies, which have been captured in broader reviews such as Huhn *et al*.^21^ Screening was conducted by a single reviewer, which may have increased the risk of selection bias. As this is a scoping review undertaken to map the available evidence, a quality appraisal or risk of bias of studies was not performed, nor was a meta-analysis of study results. Given the descriptive nature of this review, the focus is on how wearables and their data were used in clinical research studies rather than on how effective, relevant or clinically useful they are.

## Conclusion

In conclusion, this scoping review provides a comprehensive overview of how wearable device-derived data are currently used across interventional and observational clinical research studies. By mapping the disease areas most frequently studied, the wearable variables most measured, the role of wearable data as clinical outcomes or tools for disease monitoring and the extent to which ML approaches have been applied, this review highlights both areas of methodological maturity and important gaps. These findings provide a foundation to inform future study design, outcome selection and analytical approaches in clinical research using wearable technologies.

## Supplementary material

10.1136/bmjopen-2025-111027 online supplemental file 1

10.1136/bmjopen-2025-111027 online supplemental file 2

10.1136/bmjopen-2025-111027 online supplemental file 3

10.1136/bmjopen-2025-111027 online supplemental file 4

## Data Availability

All data relevant to the study are included in the article or uploaded as supplementary information.
