# Supplementary material for: Scoping review of wearable device data evaluated in clinical studies
Source: BMJ Open. 2026 Nov 2;16(11):e111027. doi: 10.1136/bmjopen-2025-111027 (PMC13735340; doi:10.1136/bmjopen-2025-111027)
Supplement: Supplementary file 1 [file bmjopen-2025-111027.reviewer_comments.pdf]

PEER REVIEW HISTORY

BMJ Open publishes all reviews undertaken for accepted manuscripts. Reviewers are asked to complete a checklist review form and are provided with free text boxes to elaborate on their assessment. These free text comments are reproduced below.

ARTICLE DETAILS

Title (Provisional)

A scoping review of wearable device data evaluated in clinical studies

Authors

Gunne, Emer; Azami-Aghdash, Saber; Holden, Sinead; Doran, Peter

---

VERSION 1 - REVIEW

**Reviewer**	**1**
**Name**	**Chee, Marcel**
**Affiliation**	**South Australia Eyre and Far North Local Health Network**
**Date**	**07-Dec-2025**
**COI**	**None**

Thank you for the opportunity to review this manuscript titled " A scoping review of wearable device data evaluated in clinical studies". The authors have conducted a timely scoping review on the current landscape of wearables in clinical studies, given the growing interest in AI diagnostics and digital health. The paper excels in several areas. The authors have appropriately provided a wide range of descriptive statistics in their Results section, and have clearly mapped out the types of devices, clinical populations and applications, and analytic approaches (traditional vs machine learning) within the included studies. I have a few major and minor points for your consideration to improve the clarity and validity of the review.

Major Comments

1. The authors should clarify the scope and aims of the paper. The main question is stated as “What is known about the collection and analysis of data extracted from wearable electronic devices used in clinical studies”. The extent and type of the analysis conducted should be specified. It appears that the authors have mainly conducted descriptive analysis without evaluating the quality of included studies or the quantitative results of included studies.

2. The abstract, discussion, and conclusion may be overstating the conclusions supported by the data. No quality appraisal of studies (e.g. risk of bias), nor evaluation of effectiveness or clinical utility of wearables was performed. The authors state “The studies included in this

review demonstrate the role played by wearables to provide continuous, real-world data … to assess health outcomes and disease progression". The review only describes that other investigation have used these wearables, not how effective, relevant, or clinically useful they were. Also, the authors state "the use of wearable devices for clinical studies in recent years has grown" which is plausible and suggested by Table 1, but is not statistically demonstrated. Please review the data and temper any claims throughout the abstract and discussion. Given the descriptive nature of the review, the conclusions should focus more on how wearables are currently being used rather than imply evidence of clinical benefit.

3. The manuscript states that qualitative content analysis was used but the procedures are described only at a high level, and the Results do not demonstrate evidence of content analysis. Please include additional methodological details (e.g. coding procedures, examples of categories or themes) or consider reframing the approach as a "descriptive synthesis".

4. The authors should account for the inconsistency between the current study yield and previous literature. This review (2020-2024) has identified 48 eligible studies, which is unexpectedly low given that the earlier scoping review cited in the manuscript ("The Impact of Wearable Technologies in Health Research: Scoping Review" Huhn et al., 2022) had identified 56 eligible studies in 2020 alone. Given this specific review was cited as a comparable study, just in an earlier time period, it would be helpful to explain why this review has captured fewer studies (e.g. if stricter eligibility criteria were used and why).

5. Following from point 4, a targeted search suggests that relevant trials e.g. the RATE-AF RCT (Gill 2024) which used consumer wearables to compare digoxin vs beta-blockers, do not appear to be included. If such studies were inadvertently missed, the search and screening strategy may require revisiting.

6. The authors should include in their methodology how missing data, if any, were handled.

7. The supplementary table showing the MeSH and keyword search terms, list of included studies, and data extraction template were not available during the review. The author or the journal should make this available for any subsequent review.

Minor comments

1. While ethics is typically not required for a review of published data, the authors may wish to include a brief statement confirming this.

2. A brief section in the Methods section listing the primary descriptive outcomes would improve clarity.

This review addresses an important and fast-developing area and has the potential to be a valuable resource. However, clarifying important aspects of the manuscript, such as the robustness of the article screening process and sharpening the conclusions from the available data, will substantially improve the manuscript's clarity and credibility.

**Reviewer**	**2**
**Name**	**Raeesi, Ahmad**
**Affiliation**	**Mashhad University of Medical Sciences**
**Date**	**08-Dec-2025**
**COI**	**None**

Thank you for the opportunity to review your manuscript. The manuscript provides a valuable overview of a highly relevant topic; however, there are some areas where the manuscript could be improved:

1- The review narrows its focus to "clinical" studies but does not provide an operational definition of this term. Wearable devices are used across many dimensions of health, like wellness, fitness, and public health. The authors must clearly define what constitutes a "clinical study" for their purposes.

2- The full search strategy, which is mentioned to be in "Supplementary Table 1," has not been provided. Without it, it is impossible to verify the reliability and comprehensiveness of the search.

3- The search strategy identified 8,953 articles, from which only 48 were selected. This very low inclusion rate raises questions about the specificity and effectiveness of the search strategy. Furthermore, the manual screening of 1,405 titles and abstracts by the team introduces a high risk of selection bias and reviewer fatigue bias, potentially compromising the selection process.

4- There are significant inconsistencies between the numbers reported in the PRISMA 2020 flow diagram (Figure 1, page 20) and the description of the screening process in the main text (Results of screening, page 9). The authors must thoroughly revise the PRISMA flow diagram and the accompanying text to ensure they are perfectly aligned and provide a clear, accurate, and auditable trail of the study selection process. Please double-check all numbers at each stage of the PRISMA flow.

5- The methods section (page 7) states the search was narrowed to "January 2020 to July 2024". However, the flow diagram confusingly reports a removal of 4,004 records because the "Search narrowed to trials 2000-2004." This is a critical contradiction that undermines the reader's understanding and the reproducibility of the search methodology.

6- The methods state a search period from January 2020 to July 2024. This is a relatively short timeframe. The authors should provide a clear justification for selecting this specific and limited period.

7- The results section is written in a descriptive style, but it rarely cites which of the 48 included studies support its statements. To improve traceability, please consider citing the specific included studies when summarizing key findings.

8- According to the Figure on page 21, an interesting finding is the relatively low proportion of studies focused on heart disease, a leading cause of mortality. The Discussion section would benefit from addressing this discrepancy. Why might the research landscape for wearables not align with global mortality statistics?

9- The limitations section on page 17 is concise. I suggest considering the whole limitation of the study.

10- The manuscript would benefit from careful number double-checking in the whole text, as well as language editing to improve clarity, grammar, and consistency in terminology.

VERSION 1 - AUTHOR RESPONSE

Reviewer: 1

Dr. Marcel Chee, South Australia Eyre and Far North Local Health Network

Comments to the Author:

Thank you for the opportunity to review this manuscript titled " A scoping review of wearable device data evaluated in clinical studies". The authors have conducted a timely scoping review on the current landscape of wearables in clinical studies, given the growing interest in AI diagnostics and digital health. The paper excels in several areas. The authors have appropriately provided a wide range of descriptive statistics in their Results section, and have clearly mapped out the types of devices, clinical populations and applications, and analytic approaches (traditional vs machine learning) within the included studies. I have a few major and minor points for your consideration to improve the clarity and validity of the review.

Response: thank you for the positive comments on our scoping review.

Major Comments

1. The authors should clarify the scope and aims of the paper. The main question is stated as "What is known about the collection and analysis of data extracted from wearable electronic devices used in clinical studies". The extent and type of the analysis conducted should be specified. It appears that the authors have mainly conducted descriptive analysis without evaluating the quality of included studies or the quantitative results of included studies.

Response: thank you for this important observation, we acknowledge that our analysis is primarily descriptive in nature. Consistent with the aims of a scoping review, we did not perform a formal assessment of study quality or quantitative synthesis of study outcomes. Rather, our goal was to identify key trends, gaps, and the range of methodologies currently employed in this rapidly evolving area. To address the reviewer's comment, we have revised the research question section in the Methods section, included the PCC framework we utilised (referenced below) and explicitly stated the primary objective of the scoping review as follows:

Identifying the research question

The main research question of this current study is: "What are the current approaches to the collection and analysis of data extracted from wearable electronic devices used in clinical research studies".

We utilised the 'PCC' framework[1] (Population, Concept, Context) to define the research question:

- **Population**: Patients participating in clinical research studies where wearable electronic devices were used to monitor their disease status.
- **Concept**: Physiological parameters are measurable factors that reflect the normal or pathological function of a biological system such as heart rate, oxygen saturation, respiratory rate, blood pressure and electrocardiogram and behavioural parameters include activity levels and sleep.
- **Context**: There are no restrictions on context, but most of the reviewed clinical studies focused on the use of wearable electronic devices in patients' natural living environments, at home.

This study is designed as a scoping review, with the primary objective of mapping existing literature on the collection and analysis of data derived from wearable electronic devices in clinical studies, including the types of devices used, clinical populations studied, data collection approaches, and analytic methods applied.

1. Pollock D, Peters MD, Khalil H, McInerney P, Alexander L, Tricco AC, et al. Recommendations for the extraction, analysis, and presentation of results in scoping reviews. JBI evidence synthesis. 2023;21(3):520-32

Additionally we have amended the data analysis section to explicitly state it is a descriptive synthesis as follows:

Data Analysis

A descriptive synthesis approach was employed to summarise and characterise the included studies.

We believe these clarifications improve the transparency of the study's objectives and better align the reader expectations with the scope of the analysis conducted.

2. The abstract, discussion, and conclusion may be overstating the conclusions supported by the data. No quality appraisal of studies (e.g. risk of bias), nor evaluation of effectiveness or clinical utility of wearables was performed. The authors state "The studies included in this review demonstrate the role played by wearables to provide continuous, real-world data … to assess health outcomes and disease progression". The review only describes that other investigation have used these wearables, not how effective, relevant, or clinically useful they were. Also, the authors state "the use of wearable devices for clinical studies in recent years has grown" which is plausible and suggested by Table 1, but is not statistically demonstrated. Please review the data and temper any claims throughout the abstract and discussion. Given the descriptive nature of the review, the conclusions should focus more on how wearables are currently being used rather than imply evidence of clinical benefit.

Response: Thank you for this important comment, we agree that some of the statements in the Abstract, Discussion, and Conclusion may overstate the inferences that can be drawn from our data. As the reviewer correctly notes, this scoping review was designed to descriptively map how wearable devices are being used in clinical studies, rather than to evaluate their effectiveness, clinical utility. We have carefully revised the Abstract, Discussion and Conclusion to better align with the descriptive nature of the review. Specifically we have rephrased the abstract conclusion to more neutral language avoiding implications of proven effectiveness or clinical utility as follows:

The studies included in this review report the use of wearables to provide continuous, real-world data of patients' physiological and behavioural metrics, to assess health outcomes and disease progression outside traditional clinical settings.

We have clarified in the study discussion limitations section as follows:
As this is a scoping review undertaken to map the available evidence, a quality appraisal or risk of bias of studies was not performed nor was a meta-analysis of study results. Given the descriptive nature of this review the focus is on how wearables and their data was used in clinical research studies rather than how effective, relevant or clinically useful they are.

We have tempered statements regarding growth in the use of wearables devices in the introduction by avoiding definitive claims and instead describing observed publication trends within included studies, without implying statistical significance as follows:

A recent review (Miyakoshi 2024)[1] reported that the number of clinical studies registered on ClinicalTrials.gov incorporating wearable devices has increased since 2012, however absolute numbers or percentages were not reported. An exploratory search of ClinicalTrials.gov using the term 'wearable device' and filtering by "First Posted Date" reveals the following counts 2012 (n=10), 2019 (n=154), 2023 (n=273). While this search does not statistically demonstrate a growth in the total number of trials involving wearable technology, it suggests an upward trend.

1. Miyakoshi, T. & Ito, Y.M. Assessing the current utilization status of wearable devices in clinical research. *Clin. Trials* **21**, 470–482 (2024). doi:10.1177/17407745241230287.

3. The manuscript states that qualitative content analysis was used but the procedures are described only at a high level, and the Results do not demonstrate evidence of content analysis. Please include additional methodological details (e.g. coding procedures, examples of categories or themes) or consider reframing the approach as a "descriptive synthesis".

Response: we have revised the manuscript Methods Data analysis section to reframe the analysis as a descriptive synthesis, which more appropriately reflects the objectives and methods of this scoping review. Our intent was to systematically organise and summarise key characteristics of the included studies rather than to conduct an in-depth qualitative thematic analysis involving formal coding procedures or theme development as follows:

Data Analysis

A descriptive synthesis approach was employed to summarise and characterise the included studies. This approach aimed to systematically organise and present key features of the literature rather than to generate or interpret qualitative themes. The first stage involved an initial familiarisation of the papers included, secondly identifying and extracting predefined data items based on the data extraction template, thirdly tabulating and grouping extracted information into structured categories to enable comparison across studies and finally reviewing the results of each element. Descriptive statistics such as frequency, percentage, mean, and median were used to report quantitative data.

4. The authors should account for the inconsistency between the current study yield and previous literature. This review (2020-2024) has identified 48 eligible studies, which is unexpectedly low given that the earlier scoping review cited in the manuscript ("The Impact of Wearable Technologies in Health Research: Scoping Review" Huhn et al., 2022) had identified 56 eligible studies in 2020 alone. Given this specific review was cited as a comparable study, just in an earlier time period, it

would be helpful to explain why this review has captured fewer studies (e.g. if stricter eligibility criteria were used and why).

Response: we agree that the difference in study yield between our review (48 studies from Jan 2020-July 2024) and the earlier scoping review by Huhn et al. (2022) warrants clarification. Our review applied more focused exclusion criteria, specifically we excluded healthy populations. In response we have added text to the Methods Information sources section as follows:

A decision was taken to narrow the search to trials from January 2020 to July 2024 to build on the broader and more heterogeneous review by Huhn et al. up to the year 2020[21], while enabling a more focused and clinically relevant analysis considering the rapid evolution and increasing maturity of wearable technologies and machine learning approaches.

5. Following from point 4, a targeted search suggests that relevant trials e.g. the RATE-AF RCT (Gill 2024) which used consumer wearables to compare digoxin vs beta-blockers, do not appear to be included. If such studies were inadvertently missed, the search and screening strategy may require revisiting.

Response: our search cut-off was 31st July 2024, RATE-AF RCT wearable sub-study was e-published in July 2024, given known delays between online publication and indexing in bibliographic databases, it is possible that some publications were not captured.

6. The authors should include in their methodology how missing data, if any, were handled.

Response: thank you for this helpful suggestion. We agree clarification on how missing data were handled strengthens the transparency of the methodology. As this was a scoping review aimed at descriptively mapping the available evidence, no imputation or statistical estimation of missing data was performed. We have added a sentence to the Methods data analysis section as follows:

Where data items were not reported in the included studies, these were recorded as “not reported” within the data extraction template and excluded from the relevant summaries where appropriate.

For specific variables such as gender and ethnicity, where reporting was inconsistent across studies, we adjusted the denominator to reflect only those studies that provided the relevant data. This ensured that reported proportions accurately represent the subset of studies with available information. We have added a sentence to the results characteristics of studies section as follows:

Gender and ethnicity were not reported by all studies, in the following calculations the denominator is adjusted to exclude those.

7. The supplementary table showing the MeSH and keyword search terms, list of included studies, and data extraction template were not available during the review. The author or the journal should make this available for any subsequent review.

Response: database search strategy and results are now included in Supplementary file 2.

Minor comments

1. While ethics is typically not required for a review of published data, the authors may wish to include a brief statement confirming this.

Response: We have now added an Ethical approval statement to our manuscript as follows:

Ethical approval and consent to participate

Not applicable. This scoping review analyses data from previously published literature. As no primary data were collected and no individual-level identifiable information was used, ethical approval was not necessary.

2. A brief section in the Methods section listing the primary descriptive outcomes would improve clarity.

Response: We have added the following sentence to the Methods data analysis section:

The primary outcomes of interest were descriptive in nature and included study characteristics (geographical distribution, sample size and study design), participant demographics (gender and ethnicity), clinical populations, types and applications of wearable devices, and data collection and analytical approaches.

This review addresses an important and fast-developing area and has the potential to be a valuable resource. However, clarifying important aspects of the manuscript, such as the robustness of the article screening process and sharpening the conclusions from the available data, will substantially improve the manuscript's clarity and credibility.

Response: thank you for taking the time to provide a detailed review of our manuscript and helping us to improve the clarity of our methods. We have now provided the database search criteria in additional file 2, errors in the PRISMA Flow diagram and results section have been corrected allowing the reader to follow the screening process accurately. Conclusions have been sharpened to avoid misinterpretation of the results.

Reviewer: 2

Dr. Ahmad Raeesi, Mashhad University of Medical Sciences

Comments to the Author:

Thank you for the opportunity to review your manuscript. The manuscript provides a valuable overview of a highly relevant topic; however, there are some areas where the manuscript could be improved:

Response: thank you for the positive comments on our scoping review and giving us the opportunity to improve our manuscript.

1- The review narrows its focus to "clinical" studies but does not provide an operational definition of this term. Wearable devices are used across many dimensions of health, like wellness, fitness, and public health. The authors must clearly define what constitutes a "clinical study" for their purposes.

Response: we have given the (PubMed) full definition of a “clinical study” in our manuscript and referenced it in our Methods study selection section as follows:

Study Selection

Clinical research studies in which wearable devices were used to collect data from participants were included in the review. The process for selecting sources of evidence was carried out by two reviewers according to the following eligibility criteria.

Inclusion criteria are outlined below:

1. Type of study: Clinical study. Defined as a work that reports on the results of a research study to evaluate interventions or exposures on biomedical or health-related outcomes[1]. The two main types of clinical research studies are interventional studies (clinical trials) and observational studies[1]. In a clinical trial participants may receive diagnostic, therapeutic, or other types of interventions, assignments are determined by the protocol. An observational study is defined as a work that reports on the results of a clinical study in which participants may receive diagnostic, therapeutic, or other types of interventions, but the investigator does not assign participants to specific interventions (as in an interventional study[1]).

1.National Institute of Health, National Library of Medicine. Publication Characteristics (Publication Types) with Scope. Available at: https://www.nlm.nih.gov/mesh/pubtypes.html (accessed November 2024).

Our review excluded healthy populations as we are looking at wearables from a clinical population trial methodology perspective rather than health and fitness industry. Please see exclusion criteria 3 as follows:

Population Exclusions: Healthy populations (either without a diagnosed condition or at risk of developing a condition)

2- The full search strategy, which is mentioned to be in "Supplementary Table 1," has not been provided. Without it, it is impossible to verify the reliability and comprehensiveness of the search.

Response: We have included the search strategies for databases in Supplementary File 2 uploaded to the journal portal and referenced in our manuscript.

3- The search strategy identified 8,953 articles, from which only 48 were selected. This very low inclusion rate raises questions about the specificity and effectiveness of the search strategy. Furthermore, the manual screening of 1,405 titles and abstracts by the team introduces a high risk of selection bias and reviewer fatigue bias, potentially compromising the selection process.

Response: please see response to 3,4,5 & 6 below

4- There are significant inconsistencies between the numbers reported in the PRISMA 2020 flow diagram (Figure 1, page 20) and the description of the screening process in the main text (Results of screening, page 9). The authors must thoroughly revise the PRISMA flow diagram and the accompanying text to ensure they are perfectly aligned and provide a clear, accurate, and auditable trail of the study selection process. Please double-check all numbers at each stage of the PRISMA flow.

5- The methods section (page 7) states the search was narrowed to "January 2020 to July 2024". However, the flow diagram confusingly reports a removal of 4,004 records because the "Search narrowed to trials 2000-2004." This is a critical contradiction that undermines the reader's understanding and the reproducibility of the search methodology.

6- The methods state a search period from January 2020 to July 2024. This is a relatively short timeframe. The authors should provide a clear justification for selecting this specific and limited period.

Response: In light of the rapid evolvement of wearable technologies in recent years and the existence of an earlier broader review Huhn et al up to 2020 a decision was taken to narrow our search to January 2020 to July 2024 inclusive. This decision reduced the number of articles by 4004 making the scoping review a viable piece of work for the review team. Manual screening of 1405 titles and abstracts was implementable given the detailed inclusion and exclusion criteria which facilitated the focus of the review.

Thank you for pointing out errors in the PRISMA Flow diagram and results section. Both have been corrected allowing the reader to follow the screening process accurately as follows:

Results of screening:

A total of 8953 studies were identified through our database search as depicted in Figure 1 PRISMA flowchart. A Covidence export of 6352 articles were subsequently opened in Endnote reference management tool where a further 405 duplicates, 24 non-English, 14 laboratories

only studies, 34 survey studies, 149 qualitative only studies, 280 reviews, 26 cross-sectional studies and 11 perspectives were manually eliminated. 1405 studies went through title and abstract screening; 201 studies were included for full text review resulting in 153 studies being excluded based on the criteria listed in the PRISMA flow diagram Figure 1. Finally, 48 studies were included in the scoping review[23-70] (Supplementary file 3 – Tables S1 to S4).

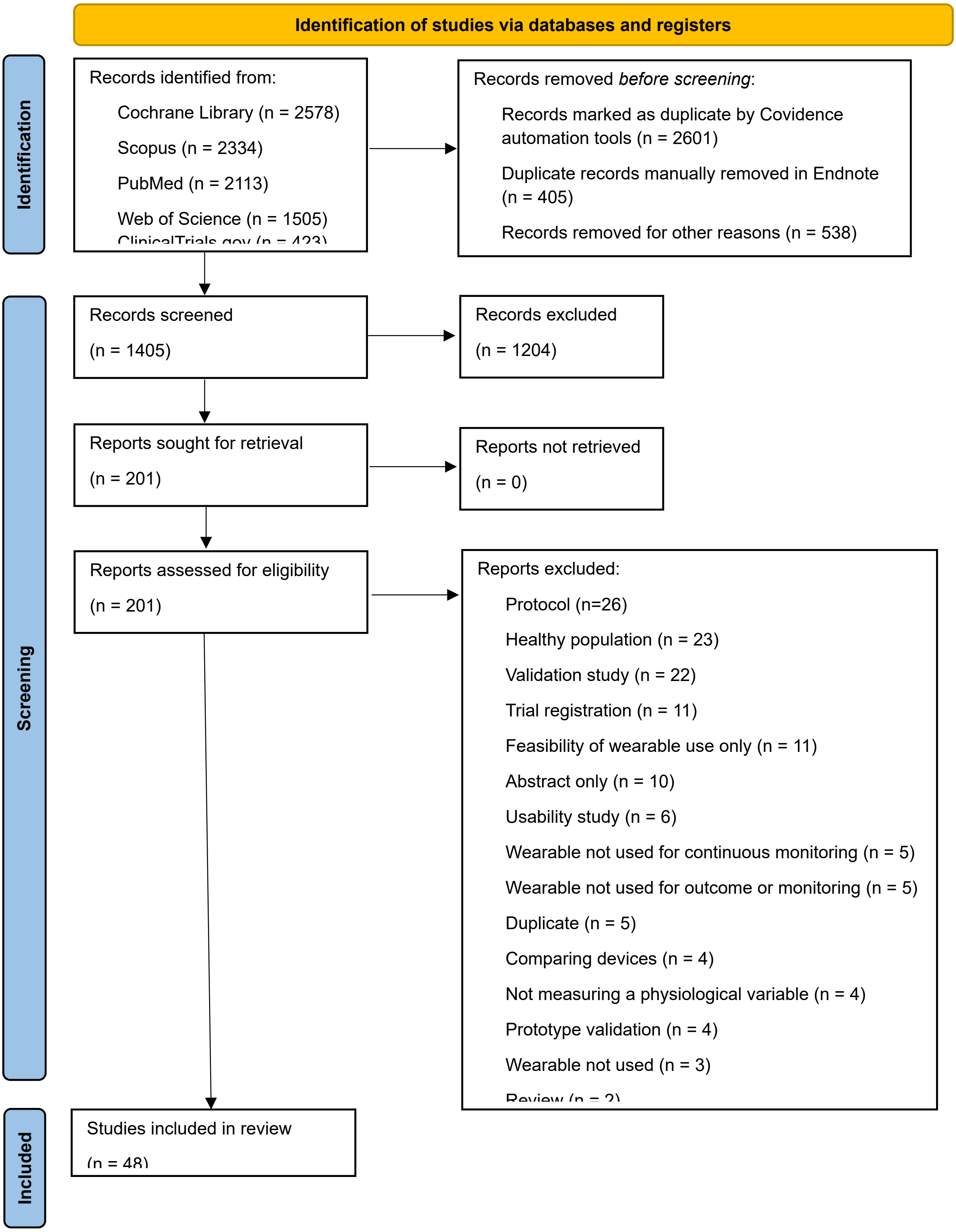

Source: Page MJ, et al. BMJ 2021;372:n71. doi: 10.1136/bmj.n71.This work is licensed under CC BY 4.0. To view a copy of this license, visit https://creativecommons.org/licenses/by/4.0/

Figure 1. PRISMA 2020 flow diagram for new reviews which included searches of databases and registers only

7- The results section is written in a descriptive style, but it rarely cites which of the 48 included studies support its statements. To improve traceability, please consider citing the specific included studies when summarizing key findings.

Response: All 48 studies have been referenced 23-70 and cited throughout the results section where possible. Data extraction tables for studies are included in Supplementary file 3 tables S1-S4.

8- According to the Figure on page 21, an interesting finding is the relatively low proportion of studies focused on heart disease, a leading cause of mortality. The Discussion section would benefit from addressing this discrepancy. Why might the research landscape for wearables not align with global mortality statistics?

Response: Thank you for pointing out the relatively low portion of studies focused on heart disease. However there are still 6 out of 48 studies which makes it the 3rd most common disease studied. We have added a section in the discussion as follows:

Although cardiovascular disease causes the most deaths globally, cardiology was only the third most studied area at 12.5%. This may reflect the suitability of wearables to monitor conditions characterised by chronic disability and fluctuating symptoms more typical of neurological conditions along with the already established clinically validated continuous monitoring tools (Holter monitors and implantable devices) for real-world monitoring of heart conditions.

9- The limitations section on page 17 is concise. I suggest considering the whole limitation of the study.

Response: Thank you for the suggestion. We have now amended the limitations in the discussion section as follows:

Limitations

Only studies published in English were included, which may have introduced language bias. The search was restricted to records from January 2020 to July 2024 to ensure relevance to rapidly evolving health technologies, although this may have excluded earlier foundational studies, which have been captured in broader reviews such as Huhn et al 2022 . As this is a scoping review undertaken to map the available evidence, a quality appraisal or risk of bias of studies was not performed nor was a meta-analysis of study results. Given the descriptive nature of this review the focus is on how wearables and their data was used in clinical research studies rather than how effective, relevant or clinically useful they are.

10- The manuscript would benefit from careful number double-checking in the whole text, as well as language editing to improve clarity, grammar, and consistency in terminology.

Response: thank you for taking the time to provide a detailed review of our manuscript and helping us to improve the clarity of our results. We have checked all numbers in our tables and made appropriate adjustments.

Reviewer: 1

If you have selected 'Yes' above, please provide details of any competing interests.: NA

Reviewer: 2

If you have selected 'Yes' above, please provide details of any competing interests.: Not applicable

---

VERSION 2 - REVIEW

**Reviewer**	**2**
**Name**	**Raeesi, Ahmad**
**Affiliation**	**Mashhad University of Medical Sciences**
**Date**	**17-May-2026**
**COI**	

Thank you for the opportunity to review your manuscript.
