# Supplementary material for: Scoping review of wearable device data evaluated in clinical studies
Source: BMJ Open. 2026 Nov 2;16(11):e111027. doi: 10.1136/bmjopen-2025-111027 (PMC13735340; doi:10.1136/bmjopen-2025-111027)
Supplement: Supplementary file 3 [file bmjopen-16-11-s002.docx]

**Database search strategy and results**

| Cochrane Library | | |
| --- | --- | --- |
| 2769 | ((wearable NEXT device*)):ti,ab,kw OR ((wearable NEXT electronic device*) OR (wearable NEXT sensor*) OR (wearable NEXT technolog*) OR (wearable NEXT system*) OR (head-mounted NEXT displa*)):ti,ab,kw OR ((body NEXT sensor*) OR (portable NEXT device*) OR (wearable NEXT solution*) OR (wearable NEXT equipment*) OR (wearable NEXT computer*)):ti,ab,kw OR ((wearable NEXT comput*) OR (smart NEXT wearable) OR (wearable NEXT activity tracker) OR (wrist-worn)):ti,ab,kw (Word variations have been searched) | #1 |
| 2602 | With Cochrane Library publication date from Jul 2014 to Jul 2024 |  |
| 8 | 8 Cochrane Reviews matching "#2 - ((wearable NEXT device*)):ti,ab,kw OR ((wearable NEXT electronic device*) OR (wearable NEXT sensor*) OR (wearable NEXT technolog*) OR (wearable NEXT system*) OR (head-mounted NEXT displa*)):ti,ab,kw OR ((body NEXT sensor*) OR (portable NEXT device*) OR (wearable NEXT solution*) OR (wearable NEXT equipment*) OR (wearable NEXT computer*)):ti,ab,kw OR ((wearable NEXT comput*) OR (smart NEXT wearable) OR (wearable NEXT activity tracker) OR (wrist-worn)):ti,ab,kw (Word variations have been searched)" with Cochrane Library publication date Between Jul 2014 and Jul 2024 |  |
| 2593 | 2593 Trials matching |  |
| 1 | 1 Editorial matching |  |
| Limit to English 2578 | | |

| PubMed | | |
| --- | --- | --- |
| 506,39 | (((((((((((((("wearable device*"[Title/Abstract]) OR ("wearable electronic device*"[Title/Abstract])) OR ("wearable sensor*"[Title/Abstract])) OR ("wearable technolog*"[Title/Abstract])) OR ("wearable system*"[Title/Abstract])) OR ("head-mounted displa*"[Title/Abstract])) OR (" body sensor*"[Title/Abstract])) OR ("portable device*"[Title/Abstract])) OR ("wearable solution*"[Title/Abstract])) OR ("wearable equipment*"[Title/Abstract])) OR ("wearable computer*"[Title/Abstract])) OR ("wearable comput*"[Title/Abstract])) OR ("smart wearable"[Title/Abstract])) OR ("wearable activity tracker"[Title/Abstract])) OR ("wrist-worn"[Title/Abstract]) OR "Wearable Electronic Devices"[Mesh] OR “Smart Glasses"[Mesh] OR “Smartglasses” OR "Head Up Display*" OR "Head Worn Display*" OR "Google Glass*" OR "Fitness Trackers"[Mesh] OR “fitness tracker*” OR “Activity Tracker*” | #1 |
| 1,012,737 | (((((("Clinical stud*"[Title/Abstract]) OR ("Clinical Trial*"[Title/Abstract])) OR ("randomized controlled trial*"[Title/Abstract])) OR ("randomized clinical trial*"[Title/Abstract])) OR ("Non-Randomized Controlled Trial*"[Title/Abstract])) OR ("Interventional stud*"[Title/Abstract])) OR ("experimental stud*"[Title/Abstract]) | #2 |
| 2,113 | #1AND #2 |  |
| Filters activated: Filters applied: Full text, in the last 10 years, English | | |

**((((((((((((((("wearable device*"[Title/Abstract]) OR ("wearable electronic device*"[Title/Abstract])) OR ("wearable sensor*"[Title/Abstract])) OR ("wearable technolog*"[Title/Abstract])) OR ("wearable system*"[Title/Abstract])) OR ("head-mounted displa*"[Title/Abstract])) OR (" body sensor*"[Title/Abstract])) OR ("portable device*"[Title/Abstract])) OR ("wearable solution*"[Title/Abstract])) OR ("wearable equipment*"[Title/Abstract])) OR ("wearable computer*"[Title/Abstract])) OR ("wearable comput*"[Title/Abstract])) OR ("smart wearable"[Title/Abstract])) OR ("wearable activity tracker"[Title/Abstract])) OR ("wrist-worn"[Title/Abstract]) OR "Wearable Electronic Devices"[Mesh] OR "Smart Glasses"[Mesh] OR "Smartglasses" OR "Head Up Display*" OR "Head Worn Display*" OR "Google Glass*" OR "Fitness Trackers"[Mesh] OR "fitness tracker*" OR "Activity Tracker*") AND ((((((("Clinical stud*"[Title/Abstract]) OR ("Clinical Trial*"[Title/Abstract])) OR ("randomized controlled trial*"[Title/Abstract])) OR ("randomized clinical trial*"[Title/Abstract])) OR ("Non-Randomized Controlled Trial*"[Title/Abstract])) OR ("Interventional stud*"[Title/Abstract])) OR ("experimental stud*"[Title/Abstract]))**

| Web of Science | | |
| --- | --- | --- |
| 54,783 | "wearable device*" (Topic) OR "wearable electronic device*" (Topic) OR "wearable sensor*" (Topic) OR "wearable technolog*" (Topic) OR "wearable system*" (Topic) OR “head-mounted displa*” (Topic) OR ” body sensor*” (Topic) OR “portable device*” (Topic) OR “wearable solution*” (Topic) OR “wearable equipment*” (Topic) OR “wearable computer*” (Topic) OR “wearable comput*” (Topic) OR “smart wearable” (Topic) OR "wearable activity tracker" (Topic) OR “wrist-worn" (Topic) | #1 |
| 746,443 | “Clinical stud*” (Topic) OR “Clinical Trial*” (Topic) OR “randomized controlled trial*” (Topic) OR “randomized clinical trial*” (Topic) OR “Non-Randomized Controlled Trial*” (Topic) OR “Interventional stud*” (Topic) OR “experimental stud*” (Topic) | #2 |
| 1,505 | #1AND #2 |  |
| Refine results for #1 AND #2 and 2024 or 2023 or 2022 or 2021 or 2020 or 2019 or 2018 or 2017 or 2016 or 2015 or 2014 (Publication Years) and Article or Proceeding Paper or Review Article (Document Types) and English (Languages) | | |

| Scopus | | |
| --- | --- | --- |
| 115,267 | (TITLE-ABS-KEY("wearable device*") OR TITLE-ABS-KEY("wearable electronic device*") OR TITLE-ABS-KEY("wearable sensor*") OR TITLE-ABS-KEY("wearable technolog*") OR TITLE-ABS-KEY("wearable system*") OR TITLE-ABS-KEY("head-mounted displa*") OR TITLE-ABS-KEY("body sensor*") OR TITLE-ABS-KEY("portable device*") OR TITLE-ABS-KEY("wearable solution*") OR TITLE-ABS-KEY("wearable equipment*") OR TITLE-ABS-KEY("wearable computer*") OR TITLE-ABS-KEY("wearable comput*") OR TITLE-ABS-KEY("smart wearable") OR TITLE-ABS-KEY("wearable activity tracker") OR TITLE-ABS-KEY(wrist-worn") OR “Smartglasses” OR "Head Up Display*" OR "Head Worn Display*" OR "Google Glass*" OR “fitness tracker*” OR “Activity Tracker*” | #1 |
| 6,605,144 | ( TITLE-ABS-KEY ( "clinical stud*" ) OR TITLE-ABS-KEY ( "clinical trial*" ) OR TITLE-ABS-KEY ( "randomized controlled trial*" ) OR TITLE-ABS-KEY ( "randomized clinical trial*" ) OR TITLE-ABS-KEY ( "non-randomized controlled trial*" ) OR TITLE-ABS-KEY ( "interventional stud*" ) OR TITLE-ABS-KEY ( "experimental stud*" ) ) | #2 |
| 2,334 | #1AND #2 |  |
| Refine results for #1 AND #2 AND PUBYEAR > 2013 AND PUBYEAR < 2025 AND  AND ( LIMIT-TO ( SUBJAREA , "medi" ) ) AND ( LIMIT-TO ( LANGUAGE , "english" ) ) | | |
