# Supplementary material for: Scoping review of wearable device data evaluated in clinical studies
Source: BMJ Open. 2026 Nov 2;16(11):e111027. doi: 10.1136/bmjopen-2025-111027 (PMC13735340; doi:10.1136/bmjopen-2025-111027)
Supplement: Supplementary file 4 [file bmjopen-16-11-s003.docx]

**Table S1. Study characteristics of included studies**

| **Study** | **Year** | **Country** | **Continent** | **Disease/Condition** | **Aim of study** | **Study design** | **N** | **Age** | **Female (%)** | **Main finding** |
| --- | --- | --- | --- | --- | --- | --- | --- | --- | --- | --- |
| Andellini et al. | 2024 | Italy | Europe | Type 1 diabetes | To identify correlations between ECG morphology variations and blood glucose in paediatric T1D | Prospective observational cohort | 36 | 9–18 years; mean 14.03 | 30.5 | ECG signals showed potential as a non-invasive adjunct for hypoglycaemia monitoring when integrated with CGM |
| Antikainen et al. | 2022 | Germany | Europe | Rheumatology | To assess feasibility of continuous physiological monitoring for fatigue and sleep disturbances | Observational pilot study | 136 | 21–82 years; mean 51.6 | 63.2 | Objective physiological measures correlated with fatigue and sleep PROs; HR recovery differed significantly across groups |
| Berry et al. | 2023 | United States | North America | Inflammatory bowel disease | To evaluate a proactive opioid-sparing analgesic protocol in hospitalised adults with IBD | Randomised controlled trial | 32 | 28–55 years; mean 36.9 | 62.5 | Protocol reduced opioid use without worsening pain; no differences in sleep or step outcomes |
| Chen et al. | 2023 | United States | North America | Lewy body dementia | To assess whether digital measures detect treatment effects in a phase 2 trial | Randomised controlled trial | 70 | 64–78 years; mean 71.44 | 14.3 | Digital measurements detected significant treatment effects at week 6, persisting to week 12 |
| Creagh et al. | 2024 | UK | Europe | Rheumatoid arthritis | To assess whether wearables and smartphones can augment PROs to determine RA status and severity | Observational study | 56 | 45–68 years; mean 58.6 | 89.0 | Daily wearable outcomes robustly distinguished RA from healthy controls |
| Deschler-Baier et al. | 2024 | Germany | Europe | Oncology | Case report of smartwatch data during selpercatinib treatment | Interventional case report | 1 | 56 years | 0 | Smartwatch data suggested early treatment efficacy before standard markers and CT imaging |
| Figueiro et al. | 2020 | United States | North America | Dementia | To evaluate circadian-effective light intervention on sleep, mood and behaviour | Interventional study | 47 | 78–92 years; mean 85.9 | 57.4 | Light intervention improved sleep efficiency and related actigraphy-derived sleep outcomes |
| Friesner et al. | 2024 | United States | North America | Oncology | To develop ML models using step count to predict hospitalisation during chemoradiotherapy | Observational secondary analysis | 214 | 53–68 years; median 61 | 47.2 | Activity-only ML approaches achieved good predictive ability for hospitalisation |
| Garcia et al. | 2023 | France | Europe | Cardiovascular disease | To test whether changes in nocturnal heart rate predict short-term cardiovascular events | Observational study | 1013 | 53–68 years; mean/median 61 | 16.0 | Dynamic nocturnal heart rate monitoring may identify impending cardiovascular events |
| Garland et al. | 2023 | United States | North America | Chronic pain | To assess mindfulness intervention triggered by wearable sensors | Randomised controlled trial | 63 | mean 53.6 | 41.3 | Just-in-time mindfulness practice was associated with increases in HRV |
| Gashi et al. | 2024 | Switzerland | Europe | Multiple sclerosis | To investigate wearable/mobile features that distinguish MS, disability and fatigue | Observational study | 79 | 25–45 years; mean 34 | 60.7 | Features from mobile and wearable sensors were feasible for MS monitoring |
| Gepner et al. | 2022 | Israel | Asia | COVID-19 | To objectively assess physiological changes after BNT162b2 vaccination | Prospective observational study | 160 | 21–78 years; mean/median 40 | 56.3 | Continuous physiological changes were detected after vaccination, especially at night and after dose 2 |
| Golbus et al. | 2021 | United States | North America | General medicine/community cohort | To collect wearable and BP data to inform clinical trial design and interpretation | Prospective community observational study | 6454 | 23–73 years; median 52 | 54.0 | BP, resting HR and activity varied by age, sex, race and ethnicity |
| Golbus et al. | 2023 | United States | North America | Heart failure | To characterise relationships between smartwatch-measured activity and patient-reported outcomes | Randomised controlled trial | 425 | 50–77 years; mean 63.5 | 44.5 | Daily step count was nonlinearly associated with health status at baseline and over time |
| Gupta et al. | 2023 | United States | North America | Amyotrophic lateral sclerosis | To quantify motor function and progression in ALS using home wearable sensors | Observational study | 596 | 21–79 years; median 57 | 34.0 | Wearable-derived scores progressed faster than ALSFRS-R and could reduce sample sizes in trials |
| Hauguel-Moreau et al. | 2020 | France | Europe | Cardiovascular disease | To measure recovery of physical activity after cardiac surgery | Prospective observational study | 100 | 58–71 years; mean 63.8 | 15.0 | Patients reached ~6000 daily steps by 2 months; 85% of this was achieved within 1 month |
| Hill et al. | 2020 | Australia | Australia | COPD | To assess the effect of a wheeled walker on physical activity and sedentary time | Randomised cross-over trial | 17 | 64–82 years; mean 73 | 41.0 | Wheeled walkers increased daily step count in selected patients with COPD |
| Jaiswal et al. | 2021 | United States | North America | General medicine / delirium | To examine whether actigraphy-based sleep fragmentation predicts delirium | Randomised controlled trial analysis | 70 | 74–89 years; mean 81 | 65.7 | Delirium was associated with increased sleep fragmentation and actigraphy may support delirium prediction |
| Kanczok et al. | 2024 | Germany | Europe | Depression | To investigate imagery rescripting and cognitive restructuring using tracker-derived activity as an assessment tool | Controlled pilot RCT | 33 | 18–76 years; mean 42 | 63.6 | Findings supported imagery-based interventions and suggested fitness trackers may aid psychopathology assessment |
| Kimura et al. | 2023 | Japan | Asia | Neurology / amyloid positivity | To predict amyloid PET positivity using wearable-derived lifestyle factors | Observational study | 122 | 71–80 years; mean 75 | 56.0 | Interpretable ML models predicted elevated amyloid burden using non-invasive variables |
| Kruizinga et al. | 2021 | Netherlands | Europe | Paediatric respiratory disease | To quantify post-discharge recovery using questionnaires and smartwatch biomarkers | Observational study | 51 | 2–9 years; mean 5 | 31.4 | Physical activity and HR digital biomarkers showed promise for quantifying recovery |
| Lönfeldt et al. | 2023 | Denmark | Europe | OCD | To capture OCD events using a wrist-worn biosensor and ML | Observational feasibility study | 8 | 10–16 years; mean 12 | 50.0 | Generalised temporal models outperformed personalised single-patient models for OCD event detection |
| Levi et al. | 2024 | Israel | Asia | COVID-19 / influenza vaccination | To predict and detect severity of vaccine side effects using smartwatch and smartphone data | Observational study | 2111 | 19–87 years; mean 49 | 50.8 | Wearables supported objective, continuous prediction and monitoring of moderate-to-severe side effects |
| Li et al. | 2021 | China | Asia | Psychiatry / COVID-19 lockdown effects | To track vital signs during COVID-19 outbreak and examine associations with anxiety/depression | Retrospective observational study | 19,960 | 24–47 years; mean 36 | 10.1 | Lockdown was associated with reduced activity, lower HR, poorer sleep quality and longer sleep duration |
| Lim et al. | 2023 | Australia | Australia | Neurology / sedative burden | To quantify impact of anticholinergic or sedative load on 24-hour activity composition | Prospective trial analysis | 183 | 78–94 years; mean 85 | 65.0 | Greater medication load was associated with increased sedentary time |
| Lin et al. | 2024 | China | Asia | Depression/anxiety | To assess whether HRV predicts response to group CBT | Randomised controlled study | 33 | 17–24 years; mean 20 | 66.7 | Higher HRV was associated with greater improvement in depressive and anxious symptoms |
| Lipner et al. | 2023 | United States | North America | Chronic pain / pachyonychia congenita | To examine associations between plantar pain and activity levels | Observational study | 24 | 36–58 years; mean 47 | 83.3 | Pain was inversely correlated with activity, suggesting utility for treatment evaluation |
| Lubitz et al. | 2022 | United States | North America | Atrial fibrillation | To evaluate a PPG-based algorithm for detecting undiagnosed AF using Fitbit | Interventional study | 455,699 | 22–75 years; mean 52 | 71.0 | Fitbit algorithm showed high PPV for concurrent AF and identified people likely to have AF on ECG monitoring |
| Maurice-Szamburski et al. | 2022 | France | Europe | Orthopaedics | To compare continuous nerve block with single-injection analgesia after ambulatory surgery | Randomised multicentre trial | 267 | 29–61 years; mean 45 | 33.4 | Continuous analgesia group had higher activity tracking values postoperatively |
| McCausland et al. | 2023 | UK | Europe | Cardiovascular disease / haemodialysis | To test safety and tolerability of spironolactone with optional cardiac monitoring | Randomised double-blind parallel trial | 57 | 43–67 years; mean 55 | 38.6 | Post hoc analysis suggested higher bradycardia and conduction block frequency with spironolactone |
| Padron et al. | 2022 | United States | North America | Oncology | To examine CBT for insomnia and pain on sleep in women with gynecologic cancer | Randomised controlled trial | 35 | 48–71 years; mean 59 | 100 | All participants demonstrated improved sleep efficiency |
| Patel et al. | 2023 | United States | North America | General medicine / readmission | To test whether post-discharge remotely monitored activity improves 30-day readmission prediction | Randomised trial | 500 | 33–60 years; mean 46 | 64.0 | Adding wearable activity data and nonparametric ML improved readmission prediction |
| Richards et al. | 2020 | New Zealand | Australia/Oceania | Oncology | To assess whether low pre-operative step count predicts adverse colorectal surgery outcomes | Observational study | 85 | 72–81 years; mean 76 | 50.6 | Low pre-operative step count predicted increased postoperative morbidity |
| Risch et al. | 2022 | Switzerland | Europe | COVID-19 | To investigate presymptomatic COVID-19 detection using wearable physiology and ML | Prospective cohort | 1163 | 38–49.65 years; mean 44 | 57.0 | RNN identified 68% of positive cases 2 days before symptom onset |
| Rutkove et al. | 2020 | United States | North America | ALS | To test whether frequent at-home self-assessment improves ALS trial efficiency | Interventional proof-of-concept study | 61 | 50–70 years; mean 60 | 29.5 | Daily measurements improved precision of progression estimates and reduced hypothetical sample size |
| Rykov et al. | 2024 | Singapore | Asia | Mild cognitive impairment | To predict cognitive scores from wearable-derived physiological features | Interventional study | 17 | 55–65 years; mean 60 | 52.9 | HRV-based digital physiological features showed potential for continuous cognitive assessment |
| Schalkamp et al. | 2023 | UK | Europe | Parkinson’s disease | To test whether accelerometry identifies prodromal Parkinson’s disease before diagnosis | Observational study | 103,712 | 40–69 years; mean 57 | 55.0 | Accelerometry may be a low-cost screening tool for identifying risk years before diagnosis |
| Sindorf et al. | 2024 | United States | North America | Stroke / sleep apnoea | To evaluate wearables and ML for detecting sleep apnoea in hospitalised stroke patients | Interventional study | 76 | 44–76 years; mean 60 | 48.7 | Wearable sensors and ML accurately detected moderate-severe sleep apnoea early after stroke |
| Temple et al. | 2023 | United States | North America | Influenza | To detect presymptomatic and asymptomatic influenza using wearable sensors | Interventional study | 20 | 18–55 years | NR | Wearable algorithm identified 16 of 17 positive individuals before symptom onset |
| Umbricht et al. | 2020 | United States | North America | Schizophrenia | To develop deep learning-based human activity recognition for negative symptoms | Randomised controlled trial | 33 | 29–43 years; mean 37 | 9.0 | Wrist-worn activity and gesture monitoring supported digital outcomes in schizophrenia trials |
| Van den Eynde et al. | 2023 | United States | North America | Barth syndrome | To identify responders to elamipretide using time-series clustering of wearable physiology | Randomised controlled trial | 12 | age range unclear; mean 19.5 | 0 | Physiological measurements from wearables predicted functional status and treatment response |
| Wang et al. | 2021 | United States | North America | Lewy body dementia | To test whether digital biomarkers detect treatment effects on cognition and motor function | Randomised controlled trial | 238 | 40–85 years; mean 73 | 16.0 | Digital metrics detected statistically significant treatment effects |
| Woelfle et al. | 2023 | Switzerland | Europe | Multiple sclerosis | To evaluate whether sleep and HR complement step count in explaining MS severity | Observational study | 54 | mean 43 | 66.7 | Activity, deep sleep and HR measures provided clinically meaningful information beyond step count |
| Wood et al. | 2023 | United States | North America | Oncology | To assess feasibility of combining wearable and patient-reported data for performance status assessment | Observational pilot multicentre study | 44 | mean 53 | 66.0 | Wearable activity and HR combined with symptom burden predicted physical function |
| Xu et al. | 2024 | United States | North America | Mild cognitive impairment | To predict MCI status from longitudinal fitness tracker data | Observational pilot study | 20 | 64–79 years; mean 71 | 55.0 | ML models separated MCI from controls; important features included HR zones and sleep variables |
| Yamagami et al. | 2021 | Japan | Asia | COVID-19 | To test whether estimated oxygen variation predicts COVID-19 symptom exacerbation | Observational pilot study | 23 | mean 51 | 70.0 | Estimated oxygen variation detected early symptom exacerbation |
| Yang et al. | 2021 | Taiwan | Asia | End-stage cancer | To predict survival outcomes using wearable actigraphy and deep learning | Observational study | 60 | 45–94 years; mean 73 | 38.3 | LSTM-based actigraphy model showed potential for predicting end-of-life admission outcomes |
| Yang et al. | 2024 | United States | North America | Cardiovascular disease | To build a cloud-based ML platform using physical activity data after discharge | Observational clinical trial | 52 | NR | NR | ML platform showed robust prediction of health outcomes during home recovery |
| Soman, P. et al. | 2025 | United States | North America | Heart failure | To investigate comparison of in-clinic assessment of 6MWT by conventional method and using wearable sensors for patients with ATTR-CM | Observational study | 12 | ATTR-CM participants in phase 3 ATTRibute-CM trial | NR | 12 enrolled at five centres; 11 evaluable. Wearable and conventional 6MWD correlation r=0.998; concordance correlation 0.997. |
| Kilic-Berkmen, G. et al. | 2024 | United States | North America | Clinical population (see study title) | To investigate an Exploratory, Randomized, Double-Blind Clinical Trial of Dipraglurant for Blepharospasm | Randomised controlled trial | 15 | NR | NR | Wearable sensor was one of multiple objective/clinical outcome measures used to assess dipraglurant response. |
| Carrillo, B. J. P. et al. | 2024 | United Kingdom | Europe | Type 2 diabetes | To investigate morning exercise and pre-breakfast metformin interact to reduce glycaemia in people with type 2 diabetes: a randomized crossover trial | Randomised controlled trial | 18 | 45–75 years (eligibility); mean 61 (8.2) | 50 | 18 completers with T2D on metformin; wearable PA variables explicitly analysed. |
| Hamm-Faber, T. E. et al. | 2025 | Netherlands | Europe | Chronic pain | To investigate is There a Correlation Between Objective Measurement Tools and Self-Reporting Questionnaires To Evaluate Physical Activity and Health Status in Patients With Persistent Spinal Pain Syndrome Type 2 Before and After Spinal Cord Stimulation? Outcomes of a Feasibility Study | Observational study | 20 | NR; mean NR; median NR | NR | 20 enrolled; 17 completed 3-month follow-up. Wearable-derived steps, distance and strenuous activity were explicitly analysed. |
| Kwasnicki, R. M. et al. | 2024 | United Kingdom | Europe | Postoperative/surgical patients | To investigate quantifying postoperative recovery using wearable activity monitors following abdominal wall surgery: The AbTech trial | Observational study | 20 | mean 47.3 (13.0) | NR | Wearable monitoring pre-op, up to 14 days post-op and at >6 months. |
| Vicenzi, M. et al. | 2026 | Italy | Europe | Interstitial lung disease | To investigate inspiratory muscle training improves exercise performance and breathing pattern during 6-min walking test in systemic sclerosis-associated interstitial lung disease | Randomised controlled trial | 24 | Adults with SSc-ILD | 83.3 | Five-week inspiratory muscle training at 60% MIP vs sham 5% MIP. |
| Ibañez-Micó, S. et al. | 2024 | Spain | Europe | Epilepsy | To investigate effect of a physical exercise program supported by wearable technology in children with drug-resistant epilepsy. A randomized controlled trial | Randomised controlled trial | 29 | Children; exact range NR in accessible source; mean NR; median NR | NR | 29 children randomized (exercise n=14, control n=15). Wearable activity was reported weekly and PA was analysed; exact wristband model not stated in accessible article text. |
| Martinique, V. B. et al. | 2026 | United Kingdom / Denmark collaboration | Europe | Knee arthroplasty / osteoarthritis | To investigate equivocal physical activity outcomes 10 Years after patellofemoral vs total knee replacement: Follow-up of a previously reported randomized controlled trial in isolated patellofemoral joint osteoarthritis | Randomised controlled trial | 30 | NR | NR | 17 PFR and 13 TKR participants wore GENEActiv for 7 days at 10-year follow-up. |
| Stein, M. et al. | 2026 | Germany | Europe | Myasthenia gravis | To investigate prediction of Myasthenia Gravis Worsening: A Machine Learning Algorithm Using Wearables and Patient-Reported Measures | Observational study | 30 | Adults with myasthenia gravis | NR | 12-week randomized study dataset; report-level objective is predictive modelling rather than treatment comparison. Best multimodal model AUROC 0.85 (95% CI 0.77–0.91). |
| Jackson, J. L. et al. | 2025 | United States | North America | Congenital heart disease | To investigate congenital Heart Disease Physical Activity Lifestyle Intervention (CHD-PAL): A Randomized Clinical Trial Among Young Adults With Congenital Heart Disease | Randomised controlled trial | 33 | 18–25 years; mean 22.2 (2.1) | 58 | 36 randomized; 33 remained eligible after post-randomization screening failures, and 30 completed assessment procedures. Primary outcome MVPA measured by ActiGraph. |
| Zhang, P. et al. | 2026 | China | Asia | Depression | To investigate real-time feedback-based personalized moderate-intensity aerobic brisk walking intervention using wrist-worn photoplethysmography (PPG) on mild-to-moderate depressive | Randomised controlled trial | 33 | Mild-to-moderate depression; young/middle-aged adults | NR | 33 completed; personalized intensity 40–59% HR reserve. Primary outcome PHQ-9; HRV secondary. |
| Adasuriya, G. et al. | 2024 | United Kingdom | Europe | Atrial fibrillation | To investigate remote monitoring of atrial fibrillation recurrence using mHealth technology (REMOTE-AF) | Observational study | 35 | mean 70.3 (6.8); median NR | NR | Prospectively designed sub-study of CASA-AF RCT; report-level analysis is observational/diagnostic. |
| Wu, C. Y. et al. | 2025 | United States | North America | Alzheimer disease / cognitive impairment | To investigate cognitive and Alzheimer's disease biomarker effects of oral nicotinamide riboside (NR) supplementation in older adults with subjective cognitive decline and mild cognitive impairment | Randomised controlled trial | 36 | Older adults with subjective cognitive decline or MCI | NR | Fitbit data available for 36 participants. Step counts significantly increased during NR vs placebo (p=0.04). Primary efficacy outcome was RBANS cognition. |
| Li, H. et al. | 2024 | China | Asia | Breast cancer | To investigate improving physical and mental health in women with breast cancer undergoing anthracycline-based chemotherapy through wearable device-based aerobic exercise: a randomized controlled trial | Randomised controlled trial | 40 | Adults | 100 | 21 exercise, 19 control; wearable-guided exercise at 50–90% HRmax during anthracycline chemotherapy. |
| Brakenridge, C. J. et al. | 2026 | Australia | Oceania | Haematological malignancy / stem-cell transplant | To investigate stem Cell Transplant Patients' Activity Decline, Outpatient Resumption, and Intervention Effectiveness Identified by Wearable Activity Monitoring | Randomised controlled trial | 41 | mean UC 53 (16); intervention 50 (15) | 34.1 | Exploratory ALLO-Active RCT analysis; 41 participants had sufficient Fitbit data. |
| Moon, H. M. et al. | 2026 | South Korea | Asia | Stroke | To investigate comparable Cardiovascular, Functional, and Lipid Profile Outcomes of High-Intensity Telerehabilitation Versus Face-to-Face Rehabilitation in Chronic Stroke: A Randomized Controlled Trial | Randomised controlled trial | 44 | Chronic stroke adults | NR | 45 enrolled, 44 analysed. Primary outcome VO2max; Apple Watch enabled remote high-intensity interval training. |
| Li, G. et al. | 2025 | China | Asia | Lung cancer | To investigate digital Therapeutics-Based Cardio-Oncology Rehabilitation for Lung Cancer Survivors: Randomized Controlled Trial | Randomised controlled trial | 47 | 18–70 years | NR | 40/47 completed. Wearable supported home cardiac telerehabilitation and quantified exercise adherence. |
| Schrauben, S. J. et al. | 2024 | United States | North America | Hypertension | To investigate supporting Self-Management of Healthy Behaviors in Chronic Kidney Disease and Hypertension : The Supporting Self-Management of Healthy Behaviors Pilot Randomized Trial | Randomised controlled trial | 47 | ≥18 years; mean NR; median 63 (52–71) | 49 | Stage 3–4 CKD plus hypertension. Step counts transmitted from Fitbit app; mean prestudy 3075 steps/day increased to 6372 in week 1 and remained >6000/day. |
| Glavas, C. et al. | 2024 | Australia | Oceania | Type 2 diabetes | To investigate exploring the Feasibility of Digital Voice Assistants for Delivery of a Home-Based Exercise Intervention in Older Adults With Obesity and Type 2 Diabetes Mellitus: Randomized Controlled Trial | Randomised controlled trial | 50 | 50–75 years; mean 66 (5); median NR | 42 | Primary outcomes were feasibility/retention and completion of prescribed advice; ActiGraph-derived physical activity was explicitly prespecified and analysed as a secondary measure. |
| Gunturkun, F. et al. | 2025 | United States | North America | Long COVID | To investigate digital Biometric Measures in Long COVID A Secondary Analysis of the STOP-PASC Randomized Clinical Trial | Randomised controlled trial | 50 | NR; mean 42.7 (13.2) | 58 | 94 enrolled in wearable substudy; 50 met wear/data completeness criteria. Randomized treatment-arm comparisons retained, so classified RCT secondary/exploratory substudy. |
| Nishio, R. et al. | 2025 | Japan | Asia | Coronary/ischemic heart disease | To investigate wearable Devices in Remote Cardiac Rehabilitation With and Without Weekly Online Coaching for Patients With Coronary Artery Disease: randomized Controlled Trial | Randomised controlled trial | 50 | Patients with coronary artery disease | NR | Both groups received Fitbit Sense with Recoval real-time monitoring; primary aim was effect of weekly online coaching on exercise capacity. |
| Gill, S. K. et al. | 2024 | United Kingdom | Europe | Clinical population (see study title) | To investigate consumer wearable devices for evaluation of heart rate control using digoxin versus beta-blockers: the RATE-AF randomized trial | Randomised controlled trial | 53 | 61–90 years; mean 75.6 (8.4); median NR | 40 | RATE-AF wearables substudy. 53 participants provided ~143.4M HR and 23.7M PA intervals over ~20 weeks; 41 had sufficient windows for CNN analysis. |
| Sunaga, A. et al. | 2026 | Japan | Asia | Atrial fibrillation | To investigate apple Watch-Guided Anticoagulation After Atrial Fibrillation Ablation　- The Up to AF Trial | Non-randomised interventional study | 54 | Post-AF-ablation patients; exact range NR | NR | Not an RCT: 54 enrolled across 9 sites; 4 had AF in first 30 days, leaving 50 in Apple Watch-guided antithrombotic strategy. Corrected prior classification. |
| Jo, Y. et al. | 2025 | United States | North America | Lung cancer | To investigate augmenting Prognostication: Utilizing Activity Trackers to Enhance Survival Prediction in Metastatic Non-Small Cell Lung Cancer | Observational study | 55 | NR; mean NR; median NR | NR | Prospective single-centre observational feasibility study in metastatic NSCLC. Fitbit metrics, especially steps, HAM and eSHR, were tested as predictors of overall survival. |
| Hartung, V. et al. | 2025 | Germany | Europe | Multiple sclerosis | To investigate internet-based exercise and physical activity promotion for persons with multiple sclerosis: a randomized controlled trial | Randomised controlled trial | 56 | NR; mean NR; median NR | NR | 62 enrolled, 56 randomized. ActiGraph objective PA outcomes were directly analysed; consumer trackers were intervention self-monitoring tools. |
| Kaur, H. et al. | 2026 | United States | North America | Cancer survivors | To investigate website Use and Associations With Behavior Change and Weight Loss in Cancer Survivors and Their Partners: Secondary Analysis of a Randomized Controlled Trial | Observational study | 56 | mean 58 (12.5) | 78.6 | 28 survivor-partner dyads. Report-level design observational secondary analysis; accelerometer MVPA directly analysed. |
| Devasahayam, A. J. et al. | 2025 | Canada | North America | Sub-acute stroke following inpatient/outpatient rehabilitation | To investigate cardiorespiratory exercise and self-management early after stroke to increase daily physical activity: results from a stepped-wedge cluster randomised trial | Randomised controlled trial | 57 | NR; mean 60.8 (group SDs: GCE 13.6; PROPEL 13.5); median NR | 45.6 | Stepped-wedge cluster superiority RCT across five Ontario rehabilitation hospitals. 172 referred to exercise groups; 59 consented; 2 withdrew before data collection; 57 analysed (GCE n=28, PROPEL n=29). Baseline age: GCE 59.9 (13.6), PROPEL 61.7 (13.5); women 14/28 and 12/29. Wearable assessment for 7 consecutive days at 1, 4 and 6 months post-discharge. Fitbit displays were disabled to prevent feedback. Primary PA guideline outcome used ≥6000 steps/day and/or HR-reserve thresholds (>40% HRR for ≥150 min/week or >60% HRR for ≥75 min/week). PROPEL participants spent more time in moderate-vigorous (p=0.034) and vigorous (p=0.011) HR ranges; steps/day did not differ (p=0.97). |
| Pinto, B. M. et al. | 2025 | United States | North America | Breast cancer | To investigate effects of web platform delivery of a physical activity program for breast cancer survivors: a randomized controlled trial | Randomised controlled trial | 61 | mean 58.10 (8.55) | 100 | Primary outcome accelerometer-measured bouted MVPA. Fitbit used for behavioral tracking/coaching. |
| Li, H. et al. | 2024 | China | Asia | Breast cancer | To investigate improving physical and mental health in women with breast cancer undergoing anthracycline-based chemotherapy through wearable device-based aerobic exercise: a randomized controlled trial | Randomised controlled trial | 40 | Adults | 100 | 21 exercise, 19 control; wearable-guided exercise at 50–90% HRmax during anthracycline chemotherapy. |
| Han, X. et al. | 2026 | China | Asia | Clinical population (see study title) | To investigate effects of dexmedetomidine nasal spray combined with thoracic paravertebral block on sleep and pain in herpes zoster neuralgia: a randomized controlled trial | Randomised controlled trial | 70 | NR; mean NR; median NR | NR | 35 DEX and 35 control. Wearable-measured total sleep time was the primary outcome; other sleep-stage measures were secondary outcomes. |
| Rillamas-Sun, E. et al. | 2026 | United States | North America | Breast cancer | To investigate comparisons of Physical Activity and Sedentary Behavior Measurements From the ActiGraph, International Physical Activity Questionnaire, and Fitbit in Women With History of Breast Cancer | Observational study | 73 | Adult women with early-stage breast cancer; exact range NR | 100 | Baseline ActiGraph/IPAQ n=73; 6-month follow-up n=50; ActiGraph-vs-Fitbit comparison n=30. Report-level design is observational measurement comparison nested within a lifestyle intervention trial. |
| Simpson, L. A. et al. | 2025 | Canada | North America | Stroke | To investigate a Randomized Control Trial of a Virtually Delivered Program for Increasing Upper Limb Activity After Stroke | Randomised controlled trial | 73 | Community-living, <1 year post stroke | NR | V-ABC n=36 vs waitlist n=37. Primary outcome was 3-day average daily reach-to-grasp counts measured by TENZR. |
| Hoffman, R. M. et al. | 2025 | United States | North America | Post-total hip arthroplasty | To investigate improvements in Physical Capacity via a Wearable Sensor May Not Indicate Reductions in Real-World Sedentary Activity: a Longitudinal Post-Total Hip Arthroplasty Study | Observational study | 76 | 50–85 years (parent RCT eligibility); mean 63.9 (7.0) | 35.5 | All 76 improved 6MWT; 45 (59.2%) also reduced sedentary time. Report-level analysis classified observational/secondary because randomized treatment effects were not the focus. |
| Montagne, B. et al. | 2026 | France | Europe | Alzheimer disease / cognitive impairment | To investigate night-to-night rapid eye movement sleep variability: A relevant marker of early amyloid-β deposition | Observational study | 76 | mean 69.1 (3.4) | 63.2 | Cognitively unimpaired older adults; multi-night wearable sleep averaged 4.5 nights. Note population eligibility may need review if disease-population criterion excludes asymptomatic amyloid-positive/negative cohorts. |
| Liu, C. et al. | 2026 | China | Asia | Shoulder arthroscopy | To investigate intraoperative esketamine infusion and postoperative sleep quality after shoulder arthroscopy: a randomized controlled trial | Randomised controlled trial | 80 | Adults with preoperative sleep disturbance | NR | 40 esketamine and 40 placebo. AIS-defined sleep disturbance was primary; wearable objective sleep metrics secondary/exploratory. |
| Hardcastle, S. J. et al. | 2024 | Australia | Oceania | Cancer survivors | To investigate a randomized controlled trial of Promoting Physical Activity in Regional and Remote Cancer Survivors (PPARCS) | Randomised controlled trial | 87 | NR; mean 63.1 (11.1) | 85.1 | Breast and colorectal cancer survivors from five Australian states. Objective ActiGraph PA was the efficacy outcome; Fitbit Charge 2 supported intervention self-monitoring. |
| Rantula, O. A. et al. | 2025 | Finland | Europe | Atrial fibrillation | To investigate photoplethysmography in recent-onset atrial fibrillation: automatic detection of rhythm change and burden | Observational study | 90 | Recent-onset AF <48 h | NR | Strong diagnostic-validation study; 10-, 20-, 30- and 60-min detection windows. |
| Pu, J. et al. | 2025 | China | Asia | Postoperative/surgical patients | To investigate effect of General Anesthesia Combined with Transversus Abdominis Plane Block on Postoperative Sleep Disorders in Elderly Patients Undergoing Gastrointestinal Tumor Surgery: A Prospective, Randomized Controlled Trial | Randomised controlled trial | 94 | 65–80 years | NR | Objective wearable sleep measures complemented PSQI-defined postoperative sleep disorder. |
| Holdom, C. J. et al. | 2025 | Australia | Oceania | Motor neuron disease | To investigate identification of passive wrist-worn accelerometry outcomes for improved disease monitoring and trial design in motor neuron disease | Observational study | 95 | NR; mean 59.95 (9.32) | 23 | Repeated 8-day wrist accelerometry every 3–4 months. Peak 6-min activity was highlighted as a promising functional disease-progression measure. |
| Stein, R. G. et al. | 2025 | Canada | North America | Stroke | To investigate the interaction effect of physical activity and sleep on cognitive function in stroke | Observational study | 97 | ≥55 years; community-dwelling chronic stroke; mean 71 (9) | 39.2 | Baseline data from a 6-month RCT; report-level design observational. Objective wrist accelerometry captured both activity and sleep. |
| Alnes, S. R. et al. | 2026 | Norway | Europe | Parkinson disease | To investigate post-rehabilitation self-management support on physical activity and nutrition, including mHealth, improves physical capacity, physical activity, and health related quality of life in people with Parkinson’s– results from a randomised controlled trial | Randomised controlled trial | 100 | mean 67.5 (9.5) | 40 | Primary outcome 6MWT; wearable-derived PA was secondary. Control group did not wear tracker. |
| Lachant, D. J. et al. | 2026 | United States / multicentre | North America | Hypertension | To investigate rodatristat ethyl decreases activity in pulmonary arterial hypertension: results from the ELEVATE-2 trial | Randomised controlled trial | 100 | NR | NR | 108 randomized in ELEVATE-2; 100 included in actigraphy analysis. |
| Schäfer, L. et al. | 2026 | Austria | Europe | Postoperative/surgical patients | To investigate early detection of postoperative infections using continuous temperature monitoring: A prospective clinical trial | Observational study | 103 | Surgical inpatients; exact range NR in extracted source | NR | Primary outcome fever detection rate. 103 patients; fever in 33, patch detected 31/33 vs infrared 12/33. |
| Werner, C. et al. | 2026 | Germany | Europe | Clinical population (see study title) | To investigate effects of a home-based exercise and physical activity intervention after inpatient rehabilitation on real-world mobility in older adults with cognitive impairment: a secondary analysis of a randomised controlled trial | Randomised controlled trial | 104 | Older adults recently discharged from geriatric rehabilitation; mean 82.3 (6.0) | 75 | Home exercise + PA promotion vs nonspecific placebo motor activity; cognitive impairment mean MMSE 23.2 (2.4). |
| Murray, R. M. et al. | 2025 | Canada | North America | Breast cancer | To investigate connecting women who are diagnosed and treated for breast cancer to engage in physical activity: a two-arm randomized controlled trial | Randomised controlled trial | 108 | Women treated for breast cancer | 100 | 54 MatchQEP and 54 peer-only Match. Device-measured MVPA was explicitly secondary. |
| Mohapatra, S. et al. | 2025 | United States | North America | Heart failure | To investigate increasing adherence and collecting symptom-specific biometric signals in remote monitoring of heart failure patients: a randomized controlled trial | Randomised controlled trial | 111 | Adults with heart failure | NR | Financial-incentive arm improved tracker adherence; daily steps correlated with daily HF symptom severity. |
| Greysen, S. R. et al. | 2022 | United States | North America | Older adults after hospitalisation | To investigate exploring Wearables to Focus on the "Sweet Spot" of Physical Activity and Sleep After Hospitalization: secondary Analysis | Observational study | 120 | ≥18 years; mean Full 120: NR; 7–9 h sleep subgroup: less-active 41.7 (13.5), active 29.8 (10.9) | NR | 232 in parent MOVE IT RCT; 120 had device sleep/step plus week-13 PRO data. Report-level analysis is observational/secondary, not randomized treatment-effect analysis. |
| Li, G. et al. | 2025 | China | Asia | Lung cancer | To investigate digital Therapeutics-Based Cardio-Oncology Rehabilitation for Lung Cancer Survivors: Randomized Controlled Trial | Randomised controlled trial | 47 | 18–70 years | NR | 40/47 completed. Wearable supported home cardiac telerehabilitation and quantified exercise adherence. |
| Dickerson, S. S. et al. | 2026 | United States | North America | Lung cancer | To investigate nurse-delivered brief behavioral treatment for insomnia in cancer survivors: a randomized controlled trial | Randomised controlled trial | 132 | mean 63.7 (SD NR in abstract) | 55 | Primary outcome ISI; wrist-actigraphy sleep metrics were prespecified secondary outcomes. |
| Wang, H. et al. | 2025 | China | Asia | Atrial fibrillation | To investigate photoplethysmography-Based Machine Learning Approaches for Atrial Fibrillation Burden: Algorithm Development and Validation | Observational study | 145 | Paroxysmal AF patients; mean 63.28 (14.23) | 33.8 | Prospective pilot validation Jan 2024–Jan 2025; smartwatch PPG sampled once/minute. |
| Blondeel, A. et al. | 2025 | Belgium | Europe | COPD | To investigate a Long-Term Physical Activity Coaching Program for Patients with Chronic Obstructive Pulmonary Disease: A Randomized Controlled Trial | Randomised controlled trial | 150 | >40 years (eligibility) | NR | 150 randomized (77 full coaching, 73 light coaching). Primary objective was change in physical activity; DynaPort MoveMonitor used for accelerometry-based outcome assessment, Fitbit for ongoing feedback/step tracking. |
| van Vliet, M. et al. | 2025 | Netherlands | Europe | Atrial fibrillation | To investigate ambulatory atrial fibrillation detection and quantification by wristworn AI device compared to standard holter monitoring | Observational study | 150 | mean 64 (12) | 40.7 | PPG and ECG algorithms achieved ≥95% sensitivity and ≥98% specificity; AF-burden correlation 99% vs Holter. |
| Zhu, B. et al. | 2025 | China | Asia | Clinical population (see study title) | To investigate application of the wireless wearable monitoring system improves the turnaround time in gastrointestinal endoscopy center | Randomised controlled trial | 166 | ≥18 years; elective painless gastroendoscopy | NR | Primary outcome was turnaround time, not a wearable variable. Wireless sensors recorded HR/SpO₂ once per second and automated NBP at set intervals. |
| Vetsch, T. et al. | 2025 | Switzerland | Europe | Postoperative/surgical patients | To investigate effect of multimodal home-based prehabilitation on objectively measured physical activity in patients undergoing elective cardiac or non-cardiac major surgery: secondary outcomes from a randomised controlled trial | Randomised controlled trial | 167 | ≥65 years; mean Cardiac 73.8 (5.3); non-cardiac 76.0 (6.0) | NR | 200 enrolled; 167 had complete PA data. High-risk elective cardiac/non-cardiac surgical patients with CPET-defined fitness deficit. |
| Ahluwalia, N. et al. | 2026 | United Kingdom | Europe | Atrial fibrillation following catheter ablation | To investigate patient-Led Smartwatch ECG Monitoring After AF Ablation: A Randomized Trial | Randomised controlled trial | 168 | NR; mean 60.5 (9.9); median NR | 31 | 449 screened; 168 randomized (85 active, 83 control). Apple Watch Series 5 active arm: daily plus symptom-/alert-triggered single-lead ECGs. Primary endpoint was time to first ECG-recorded AF recurrence after a 90-day blanking period. AF detected in 45/85 (52.9%) vs 29/83 (34.9%); HR 1.83 (95% CI 1.15–2.93), p=0.010. Median 170 (IQR 93–380) smartwatch ECGs per active participant. |
| Dong, Y. et al. | 2024 | China | Asia | Breast cancer | To investigate effect of dexmedetomidine infusion on postoperative sleep disturbances in women with breast cancer: A monocentric randomized-controlled double-blind trial | Randomised controlled trial | 188 | NR; mean NR; median NR | 100 | 240 randomized; 188 women analysed by modified intention-to-treat. Primary outcome was AIS-defined postoperative sleep disturbance; Fitbit Charge 2 sleep structure was secondary. |
| Flick, M. et al. | 2026 | Germany | Europe | Postoperative/surgical patients | To investigate abnormal vital signs during recovery from noncardiac surgery on general wards: A blinded prospective observational study | Observational study | 190 | Adults; exact range NR in accessible full-text/abstract source; mean NR; median NR | NR | Primary outcome: percentage of monitoring time with any abnormal vital sign. Device model and detailed demographics were not defensibly available in accessible source, so left NR. |
| Küng, P. et al. | 2025 | Switzerland | Europe | Obesity/overweight | To investigate health-related social control in overweight romantic couples: daily associations with physical activity and affect for targets and agents | Observational study | 198 | Adults with overweight | NR | 99 overweight romantic couples; report-level observational secondary analysis of an RCT follow-up period. |
| Vetrovsky, T. et al. | 2024 | Czech Republic | Europe | Heart failure | To investigate lifestyle Walking Intervention for Patients With Heart Failure With Reduced Ejection Fraction: The WATCHFUL Trial | Randomised controlled trial | 202 | ≥18 years; mean 65 (SD NR in abstract) | 22.8 | WATCHFUL primary report. Garmin vívofit supported self-monitoring; objective PA outcomes were measured by ActiGraph. |
| Docherty, K. F. et al. | 2024 | Multicountry | Multicontinental | Heart failure | To investigate effect of Dapagliflozin on Accelerometer-Based Measures of Physical Activity in Patients With Heart Failure: an Analysis of the DETERMINE Trials | Randomised controlled trial | 211 | mean 71.3 (8.9) | 32.2 | 211/817 randomized participants had adequate baseline and week-16 accelerometer data; 107 dapagliflozin and 104 placebo. Change in accelerometer measures at week 16 treated as primary outcome in this exploratory analysis. |
| Wagnild, J. M. et al. | 2025 | United Kingdom | Europe | Postoperative/surgical patients | To investigate accelerometer-Measured Physical Activity After Mitral Valve Surgery: An Analysis of the UK Mini Mitral Randomized Controlled Trial | Randomised controlled trial | 230 | Mitral valve repair patients | NR | 230 with valid accelerometry, 115 per arm. Randomized sternotomy vs minimally invasive minithoracotomy. |
| Wheeler, A. P. et al. | 2025 | 33 countries | Multicontinental | Haemophilia | To investigate real-World Unmet Needs of Patients With Haemophilia A and Haemophilia B With or Without Inhibitors: End-of-Study Results From the explorer6 Non-Interventional Study | Observational study | 231 | ≥12 years | NR | explorer6; severe HA, severe/moderate HB or haemophilia with inhibitors; up to 115 weeks follow-up. |
| Kumar, A. et al. | 2026 | India | Asia | Cardiovascular disease | To investigate impact of a multicomponent digital health and telemonitoring intervention on hypertension and diabetes control in cardiac patients: a randomized controlled trial | Randomised controlled trial | 240 | mean 57 (12) among 228 completers | 38 | 228/240 completed follow-up; co-primary outcomes were SBP and HbA1c, not wearable measures. |
| Saito, T. et al. | 2025 | Japan | Asia | Atrial fibrillation | To investigate an efficient approach for detecting atrial fibrillation in ischemic stroke patients using a wearable device: a prospective multicenter substudy of the STABLED trial | Observational study | 241 | NR; mean NR; median 71.0 (62.5–78.0) | 33.2 | Prospective 7-site substudy of STABLED. Primary outcome was AF detection while wearing Duranta for 7 days. Report-level design observational despite parent randomized trial. |
| Shakya, S. et al. | 2026 | United States | North America | Cardiovascular disease | To investigate social Isolation and Daily Steps Are Associated With Fatigue in Older Women With Cardiovascular Disease: A Secondary Analysis of the MindMoves Trial | Observational study | 246 | ≥65 years | 100 | Baseline data from MindMoves trial. Each additional 1000 steps/day associated with lower odds of fatigue in adjusted models. Report-level design observational. |
| Alcoceba-Herrero, I. et al. | 2024 | Spain | Europe | Clinical population (see study title) | To investigate randomized Controlled Trial to Assess the Feasibility of a Novel Clinical Decision Support System Based on the Automatic Generation of Alerts through Remote Patient Monitoring | Randomised controlled trial | 247 | <30; 30–45; 46–65; >65 (categorical only) | 57.1 | 342 randomized; 247 completed per protocol. Wearable-derived HR/SpO₂/RR/body-temperature alerts were directly analysed. |
| Solk, P. et al. | 2025 | United States | North America | Breast cancer | To investigate feasibility and acceptability of the Fit2Thrive mHealth physical activity promotion intervention components in breast cancer survivors | Randomised controlled trial | 269 | Adult inactive breast cancer survivors; mean 52.5 (9.9) | 100 | 269 randomized; 264 received intervention; retention 94%; Fitbit worn on 93% of study days. This paper focuses feasibility/acceptability rather than the parent efficacy MVPA analysis. |
| Barua, S. et al. | 2025 | United States | North America | Coronary/ischemic heart disease | To investigate adherence to Accelerometer Use in Older Adults Undergoing mHealth Cardiac Rehabilitation: Secondary Analysis of a Randomized Clinical Trial | Observational study | 271 | ≥65 years (eligibility); mean 71 (8) | 27 | Secondary analysis of intervention arm of RESILIENT RCT; report-level design observational because randomized treatment comparison was not the analysis of interest. |
| Bruna, C. L. et al. | 2026 | Multicountry (6 European countries) | Europe | Pancreatic surgery | To investigate activity and Physiological Stress Within 90 Days After Minimally Invasive and Open Pancreatoduodenectomy: A Predefined Analysis of the DIPLOMA-2 Randomized Clinical Trial | Randomised controlled trial | 288 | mean 68.3 (9.9) | 41.7 | Predefined DIPLOMA-2 RCT analysis. 288 included; Fitbit data available for 236 (82%). Main outcome was postoperative physical activity during first 90 days. |
| Sun, V. et al. | 2025 | United States | North America | Cancer surgery | To investigate comparative effectiveness of remote perioperative telemonitoring in cancer surgery: a randomized trial | Randomised controlled trial | 293 | Adults undergoing GI, GU or gynecologic cancer surgery | NR | Both arms wore wristband accelerometer and used app; RPM arm received nurse triage for threshold deviations. N=293. |
| Rethorst, C. D. et al. | 2024 | United States | North America | Breast cancer | To investigate the physical activity in cancer survivors (PACES) trial: a factorial randomized trial to optimize intervention for breast cancer survivors | Randomised controlled trial | 337 | ≥18 years; 3 months–5 years post breast-cancer treatment | 100 | 337 breast cancer survivors randomized. Objective PA measured with hip-worn ActiGraph GT3X+ at baseline, 3 and 6 months. Fitbit Alta HR was the self-monitoring intervention component, not the primary measurement device. |
| Abdelhamid, K. et al. | 2025 | Switzerland | Europe | Atrial fibrillation | To investigate fully Automated Photoplethysmography-Based Wearable Atrial Fibrillation Screening in a Hospital Setting | Observational study | 346 | 32–96; mean 72.0 (9.8); median 73 (NR) | 50.6 | Prospective single-centre clinical trial; no randomized intervention. |
| Rees-Punia, E. et al. | 2025 | United States | North America | Cancer survivors | To investigate digital intervention for increasing physical activity and reducing sedentary behaviour in cancer survivors: A trial within the Cancer Prevention Study-3 cohort | Randomised controlled trial | 415 | mean 63 (12) | 94.2 | Accelerometer data collected at baseline, 3, 6 and 12 months. |
| van Steijn, N. J. et al. | 2026 | Netherlands | Europe | Atrial fibrillation | To investigate enhanced Detection and Prompt Diagnosis of Atrial Fibrillation Using Apple Watch: a Randomized Controlled Trial | Randomised controlled trial | 437 | ≥65 years; median 75 years (IQR NR in accessible article text) | 46.7 | EQUAL trial: 219 Apple Watch vs 218 standard care. Primary outcome confirmed new-onset AF ≥30 s. New AF 9.6% vs 2.3%; HR 4.40. |
| Lim, J. S. et al. | 2025 | South Korea | Asia | Stroke | To investigate oxiracetam and physical activity in preventing cognitive decline after stroke: a multicenter, randomized controlled trial | Randomised controlled trial | 457 | mean 68.9 (8.7) | 23.9 | 500 enrolled; 457 in modified ITT. Coprimary outcomes were MMSE and CDR-SB; wearable PA was exploratory/secondary. |
| Chen, Y. et al. | 2025 | China | Asia | COPD | To investigate smartwatch-based ventilatory assessment for COPD screening: a diagnostic accuracy study | Observational study | 594 | 18–84 years across analysed cohorts; mean Training disease 54.42 (14.77), healthy 35.30 (12.35); validation COPD 65.53 (7.62), healthy 45.51 (12.15) | 45.3 | 624 enrolled; 594 analysed (476 training + 118 validation). |
| Kókai, L. L. et al. | 2025 | Netherlands | Europe | Cardiovascular disease | To investigate app-Based Physical Activity Intervention Among Women With Prior Hypertensive Pregnancy Disorder: A Randomized Clinical Trial | Randomised controlled trial | 619 | Women with prior hypertensive pregnancy disorder | 100 | Objective wearable MVPA was the main behavioral endpoint. |
| Lödding, P. et al. | 2024 | Germany | Europe | Atrial fibrillation | To investigate adherence to long-term telemonitoring-supported physical activity in patients with chronic heart failure | Randomised controlled trial | 699 | Adults with chronic heart failure | NR | Daily tracker steps remained stable in exercise arm; multivariable models examined barriers/predictors of PA progression. |
| Engdal, M. et al. | 2024 | Norway and Germany | Europe | Hip fracture | To investigate real-world mobility recovery after hip fracture: secondary analyses of digital mobility outcomes from four randomized controlled trials | Observational study | 717 | ≥65 years (source trials); mean 83.4 (6.1); median NR | 75.3 | 5909 observation days from 717 hip-fracture patients. Report-level design is observational secondary analysis rather than RCT because randomized treatment effects were not the focus. |
| Zwiers, L. C. et al. | 2025 | Netherlands / international consortium | Europe | Clinical population (see study title) | To investigate remote early detection of SARS-CoV-2 infections using a wearable-based algorithm: results from the COVID-RED study, a prospective randomised single-blinded crossover trial | Randomised controlled trial | 17825 | Adult COVID-RED participants | NR | 17,825 randomized. Wearable algorithm alerted median 0 vs 7 days before positive test; high sensitivity but low specificity. |
| Yarnall, A. J. et al. | 2026 | Multicountry Europe | Europe | Parkinson disease | To investigate the future of clinical trials? Monitoring mobility as an outcome measure in Parkinson's disease: The mobilise-D study | Observational study | 601 Parkinson disease + 232 matched controls | Parkinson disease across Hoehn & Yahr I–III | NR | Usable DMO data: 531 PD and 221 controls. Significant group differences in 20/24 DMOs. |
| Jones, T. et al. | 2026 | Australia | Oceania | Non-cystic fibrosis bronchiectasis in children | To investigate short and long-term effects of a play-based therapeutic exercise programme in children with non-cystic fibrosis bronchiectasis: A randomised controlled trial | Randomised controlled trial | 47 | 4–13 years; mean 7.4 (2.4); median NR | 53.2 | Multi-site observer-blinded parallel-group RCT at three Australian tertiary paediatric hospitals (2021–2023); 47 randomized (23 intervention, 24 control). ActiGraph GT3X+ worn on non-dominant wrist for 7 days at baseline, post-intervention, 6 and 12 months. Valid day: ≥10 h wake wear and ≥14 h total wear; inclusion required ≥3 valid days including one weekend day. Device-measured MVPA increased immediately post-intervention by net 16.8 min/day (95% CI 3.9–29.8; p=0.011) but was not sustained at 6 or 12 months. Polar OH1 HR was collected during ~25% of supervised exercise sessions for fidelity/intensity monitoring. |
| Khairat, S. et al. | 2026 | United States | North America | Cancer survivors across a variety of cancer types/stages | To investigate efficacy of Telehealth-Based Coaching to Improve Physical Activity and Overall Experience for Cancer Survivors: Secondary, Mixed Methods Analysis of a Randomized Controlled Trial | RCT – secondary mixed-methods analysis | 32 | 23–76 years; mean 58.7 (12.5); median NR | 69 | Data collected May 2020–March 2022. Parent trial randomized 46 participants; 14 were excluded from this secondary analysis because of missing or abnormal data, leaving N=32 (intervention n=20, control n=12). The paper's analytical outcomes were daily step count/daily walking distance plus intervention-participant perceptions. Fitbit data were automatically transferred through UNC CHAI Core. Intervention participants had higher average daily steps (3660 vs 3408; P=.001) and walking distance (2.6 vs 2.4 miles; P<.001); moving-average trends also increased more rapidly in the intervention arm. Eleven intervention participants contributed qualitative exit interviews. |
| Khetrapal, P. et al. | 2024 | United Kingdom | Europe | Nonmetastatic bladder cancer undergoing radical cystectomy | To investigate digital Tracking of Patients Undergoing Radical Cystectomy for Bladder Cancer: Daily Step Counts Before and After Surgery Within the iROC Randomised Controlled Trial | Observational – secondary analysis within RCT | 284 | 30–80+ years; mean NR overall; male 69.2 (8.09), female 67.12 (9.02); median NR | 20.1 | iROC recruited at nine UK centres (March 2017–March 2020). Of 308 participants eligible for activity tracking after surgery, 284 (92.2%) returned digital activity data in ≥1 time window. Misfit Shine was worn for 7-day periods at baseline and postoperatively from day 5, then weeks 5, 12 and 26. Baseline mean daily steps were 6378 (SD 3024); activity fell to 28% of baseline immediately postoperatively, recovered to 71% at week 5 and 95% at week 12. Lower postoperative step counts were associated with longer hospital stay and later medical help/readmission; baseline steps were not associated with postoperative recovery or death. Ethnicity was NR in the main paper. |
| Kosik-Rose, E. et al. | 2026 | NR (multisite phase 2 trial; paper affiliations include USA) | NR | Narcolepsy type 1 (NT1) | To investigate methods for Wearable-Derived Pulse Rate Measures and Their Application to Modeling the Relationship between Pulse Rate and Motor Activity in Narcolepsy Type 1 | Observational – baseline secondary analysis within RCT | 110 | NR; mean 34.0 (11.5); median NR | 51.8 | 112 participants were enrolled; 110 contributed baseline wearable data and formed the analytic sample. EmbracePlus was worn continuously on the nondominant wrist during the 14-day baseline, with ≥1 h charging every other day. Vendor data were supplied in nonoverlapping 1-min epochs. Approximately 23.5% of epoch-level pulse-rate observations were invalid from nonwear, missingness or artifacts. With the ≥70% valid-data threshold, 1,155/1,680 participant-days (68.8%) from 104 participants were retained, averaging 10.3 days/person. Mean pulse rates were 72.8, 83.3 and 66.2 bpm for 24-h/daytime/nighttime; mean motor activity was 40.6, 61.5 and 10.3 activity counts. Pulse rate was positively associated with motor activity in all windows (slopes 0.39, 0.30 and 0.41; all p<0.001), with conditional R² 0.76–0.80. |
| Krell, V. et al. | 2024 | Germany | Europe | Cancer survivors after completion of acute curative treatment; mixed cancer diagnoses | To investigate telemedicine-based exercise intervention in cancer survivors: a non-randomized controlled trial | Interventional – non-randomized controlled trial | 92 | 18–80 years; mean 52.3 (16.5); median 55 (range 18–80) | 40.2 | 92 participants started intervention (TE 61; RG 31); 68 completed 6-month V1 and 53 completed V2. Cancer diagnoses included prostate 24 (26.1%), breast 21 (22.8%), lymphoma 9 (9.8%), colorectal 7 (7.6%), leukemia/testicular/bladder/thyroid 5 each, and other 11. Fitbit was provided to TE participants; usable wearable data in Table 1 were available for 34 participants. During the intervention these participants averaged 10,723.9 (SD 4,304.4) steps/day and 2,258.5 (663.2) active min/week, comprising 1,836.3 light, 212.5 moderate and 231.2 vigorous min/week; ≥moderate active time 443.7 (187.0) min/week. Overall Fitbit wearing behaviour was ~80%. Ethnicity/race was NR. |
| Lee, J. et al. | 2024 | South Korea | Asia | Non–small cell lung cancer (NSCLC) undergoing curative lung cancer surgery | To investigate wearable Device–Based Intervention for Promoting Patient Physical Activity After Lung Cancer Surgery | Interventional – non-randomized clinical trial with historical control | 194 | NR; mean Intervention: 60.4 (8.7); Control: 60.2 (8.7); median NR | 50.5 | Single-centre Samsung Comprehensive Cancer Center, Seoul. Intervention n=74 and historical control n=120. Intervention group: 31 male/43 female; control: 65 male/55 female. Combined sex counts calculated directly from reported group counts (96 male, 98 female). Ethnicity/race NR. Fitbit Versa v1 monitored steps, intensity, time, frequency and exercise HR throughout the intervention. Fitbit Flex v1 was the standardized assessment device at baseline and 6 months for both groups and at 2 weeks for controls; intervention participants used Versa data at 2 weeks. Flex was worn for 7 consecutive days and quantified steps/day and MVPA by intensity. Wearable compliance was 95%. At 6 months intervention participants had 12,321 vs 10,118 daily steps (P=.007); vigorous PA 33.6 vs 18.5 min (P=.003). |
| Leps, C. et al. | 2025 | Germany | Europe | Post-surgery cancer survivors: breast, prostate and colorectal cancer after curative (R0) surgery | To investigate association between continuous device-based physical activity monitoring over six months and cardiorespiratory, metabolic and body composition outcomes in post-surgery cancer survivors - An observational cohort analysis within a randomized controlled trial (CRBP-TS Study) | Observational – secondary cohort analysis within an RCT | 111 | 18–75 years; mean 54 (11); median NR | 60.4 | Secondary cohort analysis of CRBP-TS. Original trial randomized 148 participants; 122 completed, and 111 with sufficient wearable data were included in this observational analysis. Cancer distribution: breast 65 (58.6%), prostate 33 (29.7%), colorectal 12 (10.8%); the published table totals 110 across these three categories despite N=111, so values are retained exactly as reported rather than reconciled. Garmin vívoactive 4/4s worn continuously over six months. Valid day >10 h awake-period data; valid week ≥4 valid days; ≥13 valid weeks required; mean 22 valid weeks (SD 3.6, range 13–26). Steps derived from accelerometer; MVPA from heart-rate sensor/algorithm. Mean activity: MPA 108 (88) min/week, VPA 41 (36) min/week, 8,498 (2,490) steps/day. Higher MVPA was associated with VO2max, peak power output and cardiac output; more steps were associated with VO2max, cardiac output, lower fat mass, insulin and leptin. Ethnicity/race NR. |
| Li, G. et al. | 2025 | China | Asia | Lung cancer | To investigate digital Therapeutics-Based Cardio-Oncology Rehabilitation for Lung Cancer Survivors: Randomized Controlled Trial | Randomised controlled trial | 47 | 18–70 years | NR | 40/47 completed. Wearable supported home cardiac telerehabilitation and quantified exercise adherence. |
| Liu, C. et al. | 2026 | China | Asia | Shoulder arthroscopy | To investigate intraoperative esketamine infusion and postoperative sleep quality after shoulder arthroscopy: a randomized controlled trial | Randomised controlled trial | 80 | Adults with preoperative sleep disturbance | NR | 40 esketamine and 40 placebo. AIS-defined sleep disturbance was primary; wearable objective sleep metrics secondary/exploratory. |
| Liu, D. et al. | 2024 | Australia | Oceania | Adult elective non-cardiac surgical patients admitted postoperatively to a high-dependency unit (HDU) | To investigate low-dose clonidine infusion to improve sleep in postoperative patients in the high-dependency unit. A randomised placebo-controlled single-centre trial | RCT – double-blind, placebo-controlled, parallel-group pilot effectiveness trial | 80 | ≥18 years; mean NR; median Clonidine 61 (49–70); placebo 59 (52–67) | 51.3 | CLONES trial, single academic hospital in Brisbane, Australia. 83 patients were randomized; 3 had no sleep data due to protocol errors, leaving N=80 in the modified ITT analysis (39 clonidine, 41 placebo). Baseline sex: clonidine 16M/23F; placebo 23M/18F, giving 39M/41F overall. Median ages were 61 (IQR 49–70) and 59 (52–67); an overall age mean/median was NR, so it has not been inferred. Fitbit Alta HR was worn on the wrist and measured sleep between 20:00 and 06:00. Primary result: mean total sleep time 497.2 min with clonidine vs 396.4 min with placebo; mean difference 100.8 min (95% CI 38.2–163.4), p=0.002. The paper explicitly notes that Fitbit sleep staging combines actigraphy and PPG/heart rate, but detailed HR data were not recorded, so HR is not listed as an analysed wearable variable. Trial stopped early at 83/120 randomized because funding was exhausted and COVID-19 reduced recruitment. |
| Mura, M. et al. | 2025 | France | Europe | Asymptomatic carotid atherosclerotic plaque with carotid stenosis ≥50% NASCET and no indication for carotid endarterectomy | To investigate influence of a six-month home-based individualized physical activity intervention on carotid plaque instability measured by magnetic resonance imaging: a randomized controlled clinical trial | RCT – randomized controlled, single-centre, parallel-group clinical trial | 52 | 53–89 years; mean 71 (8); median NR | 36.5 | PACAPh trial (NCT04053166), University Hospital of Lyon. 56 patients were randomized; 4 withdrew consent before first examination, leaving N=52 analysed (26/arm). Overall included cohort was reported as 22 women, mean age 71±8 years, range 53–89; among the 52 analysed patients, Table 1 gives 10F/16M in the physical-activity arm and 9F/17M in control = 19F/33M. Intervention participants wore Fitbit Alta HR throughout 6 months; controls wore it for the first and last 14 days to reduce device-induced activity/contamination. Intervention target was 7,000 steps/day or a 30% increase from the first 2 weeks, with individualized goals every 14 days. Daily steps increased significantly in the intervention arm versus control at 6 months (adjusted estimate difference 1,843±758 steps/day, 95% CI 313–3,373; p=0.02). Ethnicity was explicitly not recorded because of legal restrictions. Primary trial endpoint was change in MRI intraplaque haemorrhage score, not the wearable measure. |
| Page, L. L. et al. | 2024 | United States | North America | Breast cancer survivors; first primary stage I–IIIa breast cancer, 3–24 months after completing surgery, chemotherapy, or radiotherapy | To investigate heart Rate Monitoring Among Breast Cancer Survivors: Quantitative Study of Device Agreement in a Community-Based Exercise Program | Observational – device agreement/validation analysis nested within the intervention arm of an RCT | 14 | 38–72 years; mean 63.07 (8.66); median NR | 100 | SPARCC parent trial randomized 30 women to 12-week aerobic exercise (n=15) or usual care (n=15), but this report analysed only exercise-arm participants with valid concurrent Fitbit and Polar HR data (N=14); therefore report-level design is observational/device validation. Participants were postmenopausal women aged ≥21, stage I–IIIa breast cancer, physically inactive at entry. Fitbit Inspire HR was worn on the nondominant wrist; Polar H10 was worn on the distal sternum during supervised sessions. HR was recorded concurrently at five stages. Agreement was highest postexercise rest rc=0.89 (95% CI 0.86–0.92), then pre-exercise rest rc=0.76, cool-down rc=0.68, exercise rc=0.63, and warm-up rc=0.39. Fitbit generally overestimated HR except at midpoint exercise, where it underestimated HR. Relative error ranged −3.91% to 3.09%. The paper concludes Fitbit may estimate exercise HR but cautions against relying on it for precise exercise-prescription compliance/safety. |
| Ptomey, L. T. et al. | 2026 | United States | North America | Mild to moderate Alzheimer’s disease and related dementias (ADRD); intervention delivered to dyads of individuals with ADRD and their primary caregivers | To investigate effectiveness of a Remote Physical Activity Intervention in Individuals with Alzheimer’s Disease and their Caregivers: Results from a Randomized Trial | RCT – randomized, parallel-group, assessor-blinded dyadic trial | 99 dyads (198 individuals: 99 with ADRD + 99 caregivers) | ADRD ≥55 years; caregivers ≥18 years; mean ADRD 73.5; caregivers 69.1; median NR | ADRD 40%; caregivers 70% | NCT04102514. Trial conducted July 2020–August 2024. Dyads were randomized 1:1 to remote group video (RGV) or enhanced usual care (EUC). Although 106 dyads were randomized, 99 completed baseline testing and enrolled (RGV 57, EUC 42); 89 dyads completed the 6-month intervention (90% retention). Individuals with ADRD had mean age 73.5 years, 40% female and 9% race other than White; caregivers mean age 69.1 years, 70% female and 10% race other than White. Both groups received Fitbit Versa Lite trackers throughout the 6-month intervention; the Fitbit was worn on the nondominant wrist and used for self-monitoring/coach feedback. Objective MVPA and sedentary time were assessed at baseline, 3 and 6 months with ActiGraph wGT3X-BT worn over the nondominant hip for 7 days. At baseline 98 ADRD participants returned ActiGraph data and at 6 months 84 did; only 62 met valid wear criteria at both baseline and 6 months. No significant arm, time or arm×time effect was found for MVPA or sedentary time. The paper reports functional-fitness improvements despite no significant change in accelerometer-derived MVPA. |
| Torres-Lopez, L. V. et al. | 2024 | Spain | Europe | Children with overweight/obesity | To investigate effects of exercise on sleep in children with overweight/obesity: a randomized clinical trial | RCT – secondary outcome analysis of the ActiveBrains parallel-group randomized clinical trial | 109 randomized; main per-protocol analysis N=87 (maximum N varied by sleep outcome); baseline descriptive table N=99 | 8–11 years; mean 10.1 (1.1) years (baseline per-protocol descriptive sample, n=99); median NR | 41.4% (baseline per-protocol descriptive sample, n=99) | ActiveBrains trial, NCT02295072; data collected November 2014–June 2016 in Granada, Spain. 109 children were randomized 1:1 (control n=52; exercise n=57) to usual lifestyle or a 20-week aerobic + resistance exercise program, 3–5 sessions/week. Main efficacy analyses were per protocol (attendance ≥70%); 87 children met per-protocol criteria, while the baseline descriptive table reports n=99 (58 boys, 41 girls; mean age 10.1±1.1 years). Sleep was measured with ActiGraph GT3X+ on the nondominant wrist for 7 consecutive days, 24 h/day, at baseline, week 10 and postintervention. Sampling was 100 Hz and processing used GGIR v1.5.12 with the Sadeh sleep/wake algorithm. A valid week required ≥4 days with ≥16 h/day, including ≥3 weekdays and 1 weekend day; valid days required wear equivalent to two-thirds of night and waking hours. Sleep outcomes were total sleep time, total time in bed, sleep efficiency and WASO. The exercise intervention significantly reduced WASO by 10.796 min/day versus control (95% CI −21.085 to −0.508; p=0.040) in the per-protocol analysis; other chronic sleep outcomes were not significant. Acute attendance-versus-nonattendance night effects were nonsignificant. POLAR RS300X monitors were worn during every exercise session to track intervention intensity (average ~70% maximum HR; ~38 min/session above 80% maximum HR). Ethnicity was NR in the article. |
| Zimniak, L. et al. | 2026 | Germany | Europe | Patients undergoing elective colorectal resection for benign or malignant colorectal disease | To investigate improvement of mobility and motivation in patients with elective colorectal resection using tracking devices and utilizing self-awareness (IMPETUS): a randomized controlled trial in a traditional non-ERAS clinical setting | RCT – prospective randomized two-arm clinical trial | 62 randomized and analysed (31 intervention; 31 control) | NR; mean NR; median 66.5 [56.0–78.5] years | 38.7% | IMPETUS trial, DRKS00039731. Single-centre recruitment October 2023–April 2024; follow-up ended at hospital discharge. 70 assessed, 62 randomized 1:1 and all 62 analysed. Overall median age 66.5 years [IQR 56.0–78.5]; 38 male (61.3%) and 24 female (38.7%). Ethnicity was NR. Indications included malignant disease in 43/62 (69.4%) and benign disease in 19/62 (30.6%). All patients wore a Samsung Galaxy Watch Active 2 continuously day and night, except approximately 1 hour nightly for charging. The intervention group received hourly acoustic and vibration prompts to mobilize from 08:00–18:00; controls had passive monitoring and could view their steps. Step-count data were available for all participants. Median cumulative steps POD 0–5 were 6001 [1136–12917] in the intervention group versus 1251 [677–7081.5] in controls (p=0.039). The exploratory intervention×surgical-approach interaction was significant (p<0.001); laparoscopic subgroup IRR 2.83 (95% CI 1.63–4.89). No significant differences were found in overall complications or length of stay. |
| Rosenfeldt, A. B. et al. | 2026 | United States | North America | Idiopathic Parkinson’s disease, Hoehn and Yahr stages I–III | To investigate a Structured Aerobic Exercise Program Increases Physical Activity in People With Parkinson’s Disease: A Secondary Analysis of the CYCLE-II Trial | RCT – secondary analysis of multisite randomized controlled clinical trial | 237 analysed (118 aerobic exercise; 119 usual and customary care); 256 enrolled/randomized | NR; mean AE 62.5 (8.1); UCC 65.3 (8.2) years; median NR | 32.5% of analysed sample (40 UCC + 37 AE) | CYCLE-II, NCT04000360; multisite trial at Cleveland Clinic and University of Utah, 2019–2023. Of 256 randomized participants, 237 had sufficient activity-monitor data for this secondary analysis: AE n=118 and UCC n=119. Garmin vívofit 4 was worn around the ankle of the less affected leg during waking hours except bathing; data were synchronized weekly. Median fitted activity over 12 months was 6024 steps/day in AE versus 5062 in UCC, a 19% higher daily step count (95% CI 4%–36%, p=.010). Within AE, median activity was 8555 steps/day on exercise days versus 4636 on nonexercise days (~85% higher). Both groups showed an overall decline in activity over 12 months. Activity-monitor compliance was 91% of enrollment days in AE and 94% in UCC. Exercise adherence in AE was 94% (mean 141/150 sessions). |
| Seifert, M. et al. | 2024 | Germany | Europe | Paroxysmal or persistent atrial fibrillation undergoing first pulmonary vein isolation | To investigate impact of Physical Activity in Patients With Atrial Fibrillation Undergoing Catheter Ablation: The Multicenter Randomized BE-ACTION Trial | RCT – prospective open-label multicenter randomized controlled trial | 200 randomized and analysed by intention-to-treat (ACTION n=99; NO-ACTION n=101); per-protocol AF recurrence analysis n=173 (89/84) after excluding those without transmitted step counts | 50–77 years (eligibility criterion); mean NR; median 66 (61–71) years overall; ACTION 68.0 (63.0–72.0), NO-ACTION 65.0 (60.5–70.0) | 33.5% | BE-ACTION (DRKS00012914), 4 centres in Brandenburg, Germany; recruitment Jan 2018–Apr 2020; 12-month follow-up. Overall median age 66 (IQR 61–71); 67/200 women (33.5%) and 133/200 men (66.5%); ethnicity NR. All participants received a POLAR A370 at discharge. Steps/day increased overall from 5566 (2816–7809) at 2 weeks to 8748 (6166–11228) at 6 months and 8897 (6058–11432) at 12 months (P<0.001). Change in steps did not differ significantly between ACTION [+3205 (597–4944)] and NO-ACTION [+2423 (17–4284)], P=0.325. Walking distance/week also increased. Primary composite endpoint occurred in 27.3% ACTION vs 32.7% NO-ACTION (P=0.405). The tracker itself was present in both arms; the randomized additional intervention was remote physiotherapist review plus motivational calls in ACTION. |
| Vetrovsky, T. et al. | 2026 | Czech Republic | Europe | Heart failure | To investigate sustained Increases in Physical Activity After a Walking Intervention in Heart Failure:12-Month WATCHFUL Trial Results | Randomised controlled trial | WATCHFUL randomized cohort; 202 in parent trial | ≥18 years; mean Parent cohort mean 65 years | Parent cohort 22.8 | Companion report to #138; same unique WATCHFUL trial. Keep as separate eligible report but do not double-count trial participants. |

**Table S2. Wearable device characteristics and wear protocol**

| **Study** | **Wearable device** | **Sensor(s)** | **Type of device** | **Application of wearable** | **Study duration** | **Wear duration category** | **Wear duration / protocol** |
| --- | --- | --- | --- | --- | --- | --- | --- |
| Andellini et al. 2024 | Medtronic SEEQ Zephyr BioPatch | ECG | ECG patch, Medical-grade | Outcome assessment | 3 days | Continuous | 3 days |
| Antikainen et al. 2022 | VitalPatch | Single-lead ECG, tri-axial accelerometer, thermistor | ECG patch, Medical-grade | Outcome assessment | 1–21 days | Continuous | 24 hours |
| Berry et al. 2023 | Fitbit Charge 2 | Accelerometer, PPG | Smartwatch, Consumer-grade | Outcome assessment | Day 1 to discharge or max 7 days | Continuous | 24 hours |
| Chen et al. 2023 | Verily Study Watch | Accelerometer, gyroscope, electrodermal activity, photoplethysmography | Research-grade activity monitor | Outcome assessment | 16 weeks | Continuous | 23 hours/day |
| Creagh et al. 2024 | Apple Watch | Accelerometer, PPG | Smartwatch, Consumer-grade | Disease monitoring / objective measurement of daily-life impact, disease status and severity | 14 days | Continuous | 24 hours |
| Deschler-Baier et al. 2024 | Apple Watch Series 3 | Accelerometer, PPG | Smartwatch, Consumer-grade | Outcome assessment | NR | Continuous | 24 hours |
| Figueiro et al. 2020 | Actiwatch 2 | Accelerometer | Research-grade activity monitor | Outcome assessment | 25 weeks | Continuous | NR |
| Friesner et al. 2024 | Garmin | Accelerometer | Smartwatch, Consumer-grade | Outcome prediction | 1 week before and 4 weeks after CRT | Continuous | NR |
| Garcia et al. 2023 | LifeVest | Shirt-based HR sensing | Wearable cardioverter-defibrillator  Medical-grade | Disease monitoring | 90 days | Specific periods | Night-time only (midnight to 7 am) |
| Garland et al. 2023 | Garmin Vivosmart | Accelerometer, PPG | Smartwatch, Consumer-grade | Outcome assessment | 90 days | Continuous | 24 hours |
| Gashi et al. 2024 | Biovotion Everion | Accelerometer, barometer, electrodermal activity, temperature, photoplethysmography | Medical-grade biometric monitor | Disease monitoring | 489 days | Continuous | 24 hours |
| Gepner et al. 2022 | Biobeat chest patch sensor | Photoplethysmography | Chest patch Medical-grade | Outcome assessment | 15 days | Specific periods | 4 days |
| Golbus et al. 2021 | Apple Watch Series 3 or 4 + Omron BP monitor | Smartwatch sensors not fully specified; BP cuff for blood pressure | Smartwatch, Consumer-grade + wireless BP monitor | Disease monitoring | 90 days | Continuous | 12 hours/day |
| Golbus et al. 2023 | Fitbit Versa 2 | Accelerometer | Smartwatch, Consumer-grade | Outcome assessment | 12 weeks | Continuous | 24 hours |
| Gupta et al. 2023 | ActiGraph GT3X | Accelerometer | Research-grade activity monitor | Disease monitoring | 3 years | Continuous / repeated wear periods | Continuous for 1 week each month plus during limb-based exercises and as much as possible during the week |
| Hauguel-Moreau et al. 2020 | Withings Go | Accelerometer | Smartwatch, Consumer-grade | Outcome assessment | 60 days | Continuous | 24 hours |
| Hill et al. 2020 | StepWatch Activity Monitor and activPAL | Accelerometer | Medical-grade  Activity monitor and  Research-grade step posture/activity monitor | Outcome assessment | Minimum 3 days | Continuous | Minimum 10 hours/day |
| Jaiswal et al. 2021 | Actiwatch Spectrum Plus | Accelerometer, integrated light sensor, RGB colour light sensor | Research-grade activity monitor and sleep/circadian research | Outcome assessment | Mean hospital stay 3.7 ± 2.5 days | Continuous | 24 hours |
| Kanczok et al. 2024 | Fitbit Charge 3 | Accelerometer, PPG | Smartwatch, Consumer-grade | Outcome assessment | 21 days | Specific periods | Monday to Friday, 7 am to 9 pm |
| Kimura et al. 2023 | Silmee W20 | Accelerometer, pulse sensor, UV sensor, temperature sensor, conversation monitoring | Research-grade biometric monitor | Disease monitoring | 3 years | Continuous | 24 hours except when bathing |
| Kruizinga et al. 2021 | Withings Steel HR Smartwatch | Accelerometer, PPG, altimeter, ambient light sensor | Smartwatch, Consumer-grade | Outcome assessment | 40 days | Continuous | 24 hours |
| Lönfeldt et al. 2023 | Empatica E4 | Accelerometer, PPG, electrodermal activity sensor, temperature sensor, gyroscope | Research-grade biometric monitor | Disease monitoring | 8 weeks | Specific periods | Daytime wear |
| Levi et al. 2024 | Garmin Vivosmart 4 | Accelerometer, PPG | Smartwatch, Consumer-grade | Disease monitoring | 9 months | Continuous | 14 days monitoring window |
| Li et al. 2021 | Huawei Watch GT / Honor Watch / Honor Band | Accelerometer, PPG | Smartwatch / fitness band, Consumer-grade | Disease monitoring | 2 months | Continuous | 24 hours |
| Lim et al. 2023 | GENEActiv | Accelerometer, light sensor, temperature sensor | Research-grade activity monitor | Outcome assessment | 12 months | Specific periods | 24 hours for 1 week at baseline, 6 months and 12 months |
| Lin et al. 2024 | Huawei Band 6 | Accelerometer, PPG | Smartwatch, Consumer-grade | Outcome assessment | 28 days | Specific periods | 7 am to 9 pm for 28 days |
| Lipner et al. 2023 | Withings Activité Pop Tracker v2.0 | Accelerometer | Smartwatch, Consumer-grade | Disease monitoring | 1 year (112 days of wearable data) | Specific periods | 24 hours |
| Lubitz et al. 2022 | Fitbit + ECG patch | Accelerometer, photoplethysmography | Activity tracker Consumer-grade plus ECG patch | Disease monitoring | 5 months | Continuous | 24 hours |
| Maurice-Szamburski et al. 2022 | Fitbit | Accelerometer | Activity tracker, Consumer-grade | Outcome assessment | 90 days | Specific periods | 3 days |
| McCausland et al. 2023 | Medtronic SEEQ Mobile Cardiac Telemetry System | ECG | Medical-grade heart monitor | Disease monitoring | 36 weeks | Specific periods | 7 days of 24-hour wear before randomisation, at week 6, and during weeks 32–36 |
| Padron et al. 2022 | Actiwatch-L | Accelerometer, ambient light sensor | Research-grade activity monitor | Outcome assessment | 18 weeks | Continuous | 24 hours/day except bathing or swimming |
| Patel et al. 2023 | Withings Steel | Accelerometer | Smartwatch, Consumer-grade | Disease monitoring | 6 months | Continuous | 24 hours |
| Richards et al. 2020 | Garmin vivofit 3 | Accelerometer | Smartwatch, Consumer-grade | Outcome prediction | 44 days | Continuous | 24 hours |
| Risch et al. 2022 | Ava-bracelet | Accelerometer, PPG, skin temperature, breathing rate, perfusion sensor | Medical grade -fertility aid repurposed for monitoring | Disease monitoring | 9 months | Continuous | 24 hours |
| Rutkove et al. 2020 | Mi Band | Accelerometer, PPG | Activity tracker, Consumer-grade | Outcome assessment | 9 months | Continuous | 24 hours |
| Rykov et al. 2024 | Empatica E4 | Accelerometer, PPG, electrodermal activity, temperature | Research-grade biometric monitor | Disease monitoring | 10 weeks | Specific periods | Wear as much as possible |
| Schalkamp et al. 2023 | UK Biobank accelerometer Axivity AX3 | Accelerometer | Research-grade activity monitor | Disease monitoring | Data collected 2013–2015 | Continuous | 24 hours |
| Sindorf et al. 2024 | ActiWatch Spectrum watch | Accelerometer | Research-grade activity monitor | Outcome assessment | Inpatient days | Specific periods | Minimum 1 night |
| Temple et al. 2023 | Bittium Faros 180 | Accelerometer, ECG | ECG patch, medical-grade | Outcome assessment | 17 days | Continuous | Removed only for showering |
| Umbricht et al. 2020 | GENEActiv actigraphy device | Accelerometer | Research-grade activity monitor | Disease monitoring | 15 weeks | Continuous | 24 hours |
| Van den Eynde et al. 2023 | AVIVO mobile patient management system (Medtronic) | ECG electrodes, accelerometer, impedance sensor, temperature sensor | ECG patch, medical-grade | Disease monitoring | 12 weeks | Specific periods | 7 consecutive days after screening, baseline 1, baseline 2, End 1 and End 2 |
| Wang et al. 2021 | AX3 Axivity | Accelerometer, temperature sensor, ambient light sensor | Research-grade biomteric monitor | Disease monitoring | 12 weeks | Specific periods | Three 2-week periods: pre-, during, and post-intervention |
| Woelfle et al. 2023 | Fitbit Versa 2 | Accelerometer, PPG, SpO2 sensor, altimeter, ambient light sensor, vibration motor | Smartwatch, Consumer-grade | Outcome assessment | 6 weeks | Continuous | 24 hours |
| Wood et al. 2023 | Fitbit Charge HR | Accelerometer, PPG, altimeter | Smartwatch, Consumer-grade | Outcome assessment | 6 weeks | Continuous | At least 8 hours/day |
| Xu et al. 2024 | Fitbit Charge 4 | Accelerometer, PPG, SpO2 sensor, altimeter, ambient light sensor, vibration motor, GPS | Smartwatch, Consumer-grade | Disease monitoring | 1 month | Continuous | 24 hours |
| Yamagami et al. 2021 | Fitbit Charge 3 | Accelerometer, PPG, altimeter, SpO2 sensor | Smartwatch, Consumer-grade | Disease monitoring | 30 days | Continuous | 24 hours |
| Yang et al. 2021 | Actigraphy XB40ACT | Accelerometer | Research-grade activity monitor | Outcome assessment | 1 year | Continuous | During hospital stay |
| Yang et al. 2024 | Rockband | Accelerometer | Activity tracker, Consumer-grade | Disease monitoring | 28 days | Continuous | 24 hours |
| Soman, P. et al. 2025 | APDM wearable sensors / Opal system (feet and lower back) | 6-minute walk distance (6MWD) | Research grade | Disease monitoring | single 6-minute walk test | Specific period | Wearable sensors were attached during the concurrent 6MWT; wearable measurement lasted the 6-minute test. |
| Kilic-Berkmen, G. et al. 2024 | Custom wearable sensor; | Blink/eyelid movement-derived blepharospasm measures | Research grade | Disease monitoring | NR | Specific period | Wearable sensor recorded standardized tasks lasting <5 minutes at baseline and post-dose assessment epochs. |
| Carrillo, B. J. P. et al. 2024 | Garmin vívosmart 4; FreeStyle Libre 2 CGM | Steps/day; heart rate; sleep quality; continuous glucose | Consumer grade (Garmin); medical glucose sensor (CGM) | Secondary Endpoint | 16 weeks | Continuous | 2-week baseline; two 6-week exercise arms with washout within a 16-week crossover trial |
| Hamm-Faber, T. E. et al. 2025 | Fitbit | Steps/day; distance/day; strenuous physical activity; body position/mobility | Consumer/research grade unclear — verify device model | Disease monitoring | 3 months | NR | Objective activity measures assessed longitudinally from baseline to 3 months |
| Kwasnicki, R. M. et al. 2024 | Axivity AX3 | Physical activity / functional mobility relative to preoperative baseline | Research grade | Primary Endpoint | 14 days postoperative (plus later assessment) | NR | Monitoring pre-operatively and up to 14 days post-operatively, with later >6-month assessment |
| Vicenzi, M. et al. 2026 | Combined wearable metabolic system | Minute ventilation; respiratory frequency; tidal volume; ventilatory equivalent for CO2; oxygen uptake during 6MWT | Research/clinical grade | Secondary Endpoint | 5 weeks | NR | Wearable-derived exercise measures collected across the 5-week inspiratory muscle training study |
| Ibañez-Micó, S. et al. 2024 | FitBit Flex 2 | Physical activity / weekly exercise adherence | Consumer grade / exact model NR | Secondary Endpoint | NR | NR | NR |
| Martinique, V. B. et al. 2026 | GENEActiv | Inactivity; light/moderate/vigorous activity; total PA; step count; ENMO acceleration | Research grade | Primary Endpoint | 7 days | NR | GENEActiv worn for 7 days at the 10-year trial follow-up |
| Stein, M. et al. 2026 | Garmin Vivosmart 5 | Wearable activity/physiological signals combined with PROMs and patient-performed measures | Consumer/research grade | Disease monitoring | 12 weeks | NR | Wearable data collected alongside PROMs during 12-week study |
| Jackson, J. L. et al. 2025 | ActiGraph wGT3X-BT (outcome); Fitbit model NR | Moderate-to-vigorous physical activity (MVPA), min/day | Research grade outcome device + consumer grade intervention device | Primary Endpoint | 12 weeks | NR | CHD-PAL randomized intervention followed participants through 12 weeks with ActiGraph-based PA outcomes. |
| Zhang, P. et al. 2026 | Wrist-worn PPG device (exact model NR in accessible article text) | Heart rate; HRV including RMSSD; exercise intensity / heart-rate reserve | Research/consumer grade unclear | Secondary Endpoint | 6 weeks | NR | N-of-1 randomized crossover conducted over 6 weeks |
| Adasuriya, G. et al. 2024 | Fitbit Charge 2 | ECG/rhythm/AF burden | Consumer grade | Disease monitoring | 6 months | NR | REMOTE-AF remotely monitored AF recurrence with mHealth/wearable technology over 6-month follow-up. |
| Wu, C. Y. et al. 2025 | Fitbit Charge 3 (n=8) or Fitbit Charge 4 (n=28) | Daily total step count; average daily steps over final 14 days of each block; wear adherence; percent change in steps from baseline | Consumer grade | Disease monitoring | Two 10-week treatment blocks | Specific periods | Daily steps; primary wearable summary used the final 14 days of each treatment block |
| Li, H. et al. 2024 | NR in accessible source | Exercise heart rate/intensity; physical activity energy expenditure (PAEE); exercise adherence | Consumer/clinical grade unclear | Disease monitoring | 12 weeks | NR | Wearable-guided aerobic exercise monitoring across the 12-week anthracycline chemotherapy intervention |
| Brakenridge, C. J. et al. 2026 | Fitbit Ionic or Fitbit Versa 2; ActiGraph GT3X and activPAL3 comparators | Steps; sedentary time; light PA; MVPA; heart-rate-derived MVPA | Consumer grade (primary continuous wearable); research-grade comparators | Secondary Endpoint | 28 days | NR | Activity trajectory monitored over approximately four weeks around stem-cell transplantation |
| Moon, H. M. et al. 2026 | Apple Watch | Exercise heart rate / maximum HR / walking HR | Consumer grade | Secondary Endpoint | 6 weeks | NR | Exercise heart-rate monitoring during a 6-week telerehabilitation/rehabilitation programme |
| Li, G. et al. 2025 | NR in accessible source | Exercise duration; effective exercise duration; exercise prescription adherence / activity | Consumer/clinical grade unclear | Primary Endpoint | 84 days | Continuous | Fitbit Charge continuously measured steps and sleep for 84 days |
| Schrauben, S. J. et al. 2024 | Fitbit (exact model not stated in article) | Daily step counts; step-count adherence | Consumer grade | Primary Endpoint | 12 weeks | NR | 12-week randomized crossover pilot using wearable-supported self-management. |
| Glavas, C. et al. 2024 | ActiGraph GT9X Link | Sedentary time; light PA; moderate PA; vigorous PA; very vigorous PA; MVPA; steps/day | Research grade | Secondary Endpoint | 12 weeks | NR | 12-week home-based intervention delivered and monitored by digital voice assistants |
| Gunturkun, F. et al. 2025 | Apple Watch Series 4 or newer | Physical activity; steps; heart rate; resting/walking HR; HRV; SpO₂; respiratory rate; ECG; blood pressure platform data | Consumer grade | Secondary Endpoint | ~15 weeks | Continuous | Continuous smartwatch digital-biometric data analysed over approximately 15 weeks |
| Nishio, R. et al. 2025 | Fitbit Sense | Heart rate; exercise/physical activity metrics; remote exercise adherence | Consumer grade | Secondary Endpoint | 12 weeks | NR | Wearable-derived daily activity assessed from baseline through the 12-week remote cardiac rehabilitation intervention |
| Gill, S. K. et al. 2024 | Fitbit Charge 2 | Heart rate; step count/physical activity | Consumer grade | Primary Endpoint | ~20 weeks | NR | Wearable HR and physical-activity data were collected over approximately 20 weeks. |
| Sunaga, A. et al. 2026 | Apple Watch | AF/rhythm recurrence detected by Apple Watch; ECG/rhythm notifications | Consumer grade | Disease monitoring | 12 months | Continuous | Apple Watch-guided AF recurrence monitoring across 12-month follow-up |
| Jo, Y. et al. 2025 | Fitbit (exact model NR in accessible full text) | Steps; distance; heart rate; activity intensity; derived HAM and excessive sedentary heart rate (eSHR) | Consumer grade | Disease monitoring | NR | NR | NR |
| Hartung, V. et al. 2025 | ActiGraph wGT3X-BT (outcome); Garmin vívofit 4 or Fitbit Inspire (intervention/self-monitoring) | Steps/day; device-measured MVPA minutes | Research grade outcome device + consumer grade intervention devices | Primary Endpoint | 12 weeks | NR | Consumer activity-monitor outcomes collected over the 12-week intervention |
| Kaur, H. et al. 2026 | Accelerometer model NR in accessible source | Moderate-to-vigorous physical activity (MVPA) | Research grade / model NR | Secondary Endpoint | 7 days per assessment | NR | ActiGraph worn for 7 days; valid wear ≥4 days with ≥600 min/day |
| Devasahayam, A. J. et al. 2025 | Fitbit Charge HR; Fitbit One for participants using a standard or wheeled walker | Step count; heart rate; resting heart rate; time in moderate-to-vigorous HR range; time in vigorous HR range; adherence to physical activity guidelines | Consumer grade | Primary Endpoint | 7 days at 1, 4 and 6 months | Specific periods | Seven-day wearable assessments at months 1, 4 and 6 |
| Pinto, B. M. et al. 2025 | ActiGraph GT3X; Fitbit (exact model NR) | Bouted MVPA; total MVPA | Research grade outcome device + consumer grade intervention tracker | Primary Endpoint | 12 weeks | NR | Wearable physical-activity outcomes compared from baseline to 12 weeks |
| Li, H. et al. 2024 | Fitbit (exact model NR) | Total sleep time; WASO; number of awakenings; time in bed; sleep efficiency | Consumer grade | Disease monitoring | 7 days | NR | Fitbit and sleep-diary measures collected concurrently across a 7-day assessment |
| Han, X. et al. 2026 | HUAWEI Band 9 | Total sleep time; deep sleep time; light sleep time; REM sleep time; number of awakenings | Consumer grade | Primary Endpoint | NR | NR | NR |
| Rillamas-Sun, E. et al. 2026 | ActiGraph (model NR in current article evidence); Fitbit Inspire | Vigorous PA; MVPA; sedentary time; Fitbit physical activity | Research grade + consumer grade | Disease monitoring | 7-day ActiGraph assessments; Fitbit longitudinal | Specific periods | ActiGraph assessment windows were 7 days; Fitbit comparison included longitudinal intervention follow-up |
| Simpson, L. A. et al. 2025 | TENZR | Daily reach-to-grasp counts of affected upper limb | Research/rehabilitation grade | Primary Endpoint | 3-week intervention; 3-day outcome windows | NR | Wearable outcome captured in 3-day windows around the 3-week virtually delivered programme |
| Hoffman, R. M. et al. 2025 | ActiGraph wGT3X-BT | Percentage of active wear time in sedentary activity | Research grade | Secondary Endpoint | NR | NR | NR |
| Montagne, B. et al. 2026 | Somno-Art | Total sleep time; REM sleep duration; night-to-night REM variability; other sleep architecture measures | Research/clinical-grade sleep monitor | Disease monitoring | 4.5 nights mean | NR | Somno-Art assessment over mean 4.5 (SD 0.8) nights |
| Liu, C. et al. 2026 | Wrist-worn wearable; exact model NR in accessible full text | Total sleep time; sleep efficiency; wake after sleep onset; deep/light/REM sleep | Consumer grade / model NR | Primary Endpoint | NR | NR | NR |
| Hardcastle, S. J. et al. 2024 | ActiGraph GT9X (outcome); Fitbit Charge 2 (intervention) | MVPA; moderate PA; light PA; sedentary time; bouted MVPA; wear time | Research grade outcome device + consumer grade intervention device | Primary Endpoint | 12 weeks | NR | Fitbit-supported PA promotion with objective ActiGraph outcomes over 12 weeks |
| Rantula, O. A. et al. 2025 | Three-channel PPG armband (exact commercial model NR) | PPG rhythm; AF detection; AF burden; AF-to-sinus-rhythm change | Research/clinical validation device | Disease monitoring | NR | NR | NR |
| Pu, J. et al. 2025 | Honor 7 smart band | Deep/light/REM sleep proportions; awakenings; sleep continuity; sleep respiratory quality | Consumer grade | Secondary Endpoint | NR | NR | NR |
| Holdom, C. J. et al. 2025 | ActiGraph GT9X Link | Accelerometry-derived activity outcomes including peak 6-min activity and longitudinal activity measures | Research grade | Disease monitoring | 8 days every 3–4 months | NR | Eight-day wrist accelerometry assessments repeated approximately every 3–4 months |
| Stein, R. G. et al. 2025 | Wrist accelerometer; exact model NR in abstract evidence reviewed | MVPA; light PA; sleep efficiency; sleep latency; sleep duration | Research grade | Disease monitoring | 7 days | NR | Baseline accelerometer-derived physical activity and sleep obtained over a 7-day wearable assessment |
| Alnes, S. R. et al. 2026 | Garmin vívosmart 4 | Daily steps; heart rate; weekly intensity minutes | Consumer grade | Secondary Endpoint | 6 months | NR | Device-derived physical activity followed longitudinally through 6 months |
| Lachant, D. J. et al. 2026 | ActiGraph (exact model NR) | Peak 5-min steps; daily steps; moderate activity time; vigorous activity time | Research grade | Secondary Endpoint | 24 weeks | NR | Wearable activity outcome followed across the 24-week randomized treatment period |
| Schäfer, L. et al. 2026 | SteadyTemp® patch | Continuous axillary body temperature; fever ≥38.0°C; fever timing/duration | Medical/clinical grade | Primary Endpoint | 7 postoperative days | Continuous | Continuous wearable temperature monitoring during the first postoperative week |
| Werner, C. et al. 2026 | Single body-fixed sensor; exact model in full methods | Walking amount; walking-bout pattern; walking speed; stride length; 90th-percentile walking speed and other digital mobility outcomes | Research grade | Secondary Endpoint | NR | NR | NR |
| Murray, R. M. et al. 2025 | Fitbit Inspire 2 | Device-measured MVPA; light PA | Consumer grade | Secondary Endpoint | NR | NR | NR |
| Mohapatra, S. et al. 2025 | Fitbit activity tracker (exact model NR) | Daily steps; tracker adherence | Consumer grade | Primary Endpoint | 180 days | NR | Prospective 180-day monitoring study |
| Greysen, S. R. et al. 2022 | Withings Activité | Daily steps; sleep duration | Consumer grade | Disease monitoring | NR | NR | NR |
| Li, G. et al. 2025 | Fitbit (exact model NR) | Daily physical activity time; daily awake sedentary time; sitting/walking habits | Consumer grade | Primary Endpoint | 84 days | Continuous | Fitbit Charge continuously measured steps and sleep for 84 days |
| Dickerson, S. S. et al. 2026 | Philips Respironics Actiwatch Spectrum Plus | Sleep onset latency; wake after sleep onset; total sleep time; sleep efficiency | Research grade | Secondary Endpoint | NR | NR | NR |
| Wang, H. et al. 2025 | Huawei Watch GT 2 Pro | Minute-level PPG; AF episode duration/frequency; AF density; AF variability; rapid ventricular rate; AF burden | Consumer grade | Disease monitoring | Prospective pilot Jan 2024–Jan 2025; individual wear duration NR | NR | Smartwatch PPG sampled once per minute; exact participant-level wear span NR in current extraction |
| Blondeel, A. et al. 2025 | DynaPort MoveMonitor for outcome accelerometry; Fitbit activity tracker for coaching/feedback | Steps/day; objectively measured physical activity; Fitbit daily step count | Research grade (DynaPort outcome device) + consumer grade (Fitbit intervention) | Primary Endpoint | 12 months | NR | Fitbit used for ongoing coaching/step feedback; DynaPort objectively measured PA at outcome assessments including 6 and 12 months |
| van Vliet, M. et al. 2025 | Corsano CardioWatch 287-2 (CW2) | PPG rhythm; single-lead ECG; AF detection; AF prevalence; AF burden | Medical/clinical validation device | Disease monitoring | 27-day extended monitoring after 24-h Holter validation | NR | Head-to-head validation against 24-h Holter followed by 27 days of extended wrist-worn monitoring |
| Zhu, B. et al. 2025 | CareClever® Wireless Multi-Parameter Patient Monitoring System, MC10 Series (Raiing Medical, China) | ECG; heart rate; SpO₂; non-invasive blood pressure; physiological alarms | Medical/clinical grade | Secondary Endpoint | Per gastrointestinal endoscopy episode | NR | Wearable monitoring during gastrointestinal endoscopy workflow; no longer-term wear protocol reported |
| Vetsch, T. et al. 2025 | Research accelerometer; exact model reported in article methods | Steps/day; raw acceleration metrics; physical activity | Research grade | Secondary Endpoint | NR | NR | NR |
| Ahluwalia, N. et al. 2026 | Apple Watch Series 5 | PPG-based heart rate/rhythm monitoring; single-lead ECG; AF recurrence/rhythm detection | Consumer grade | Primary Endpoint | 12 months | Continuous | Apple Watch ECG monitoring after catheter ablation across 12-month follow-up |
| Dong, Y. et al. 2024 | Fitbit Charge 2 | Sleep structure: nocturnal wakefulness, deep sleep and other sleep-duration/stage measures | Consumer grade | Secondary Endpoint | Preoperative through postoperative day 3 | Specific periods | Sleep assessed preoperatively and on postoperative days 1 and 3 |
| Flick, M. et al. 2026 | NR in accessible source | SpO₂; respiratory rate; systolic blood pressure; pulse/heart rate | Unclear — verify from full text/device documentation | Primary Endpoint | Postoperative ward recovery period | NR | Blinded wearable vital-sign monitoring during recovery on general wards; exact device/model and total hours NR in accessible source |
| Küng, P. et al. 2025 | Accelerometer model NR in accessible source | Daily physical activity | Research grade | Primary Endpoint | 14 days | NR | Secondary analysis of a 14-day follow-up using activity trackers and daily questionnaires |
| Vetrovsky, T. et al. 2024 | Garmin vívofit; ActiGraph wGT3X-BT | Daily step count; moderate-to-vigorous physical activity (MVPA) | Consumer grade + research grade | Secondary Endpoint | NR | NR | NR |
| Docherty, K. F. et al. 2024 | Waist-worn triaxial accelerometer; brand/model NR waist-worn, tri-axial seismic accelerometer device with a sampling frequency of 100 Hz (Dynaport MoveMonitor, McRoberts, the Hague, Netherlands | Steps; light-to-vigorous PA; MVPA; walking movement intensity; vector magnitude units; total activity counts | Research grade | Primary Endpoint | 7 days at 3 trial time points | Specific periods | Accelerometer ideally 24 h/day for 7 days at baseline, week 8 and weeks 14–16 |
| Wagnild, J. M. et al. 2025 | Wrist-worn triaxial accelerometer (exact model in full methods) | Total activity counts; MVPA; light PA; sedentary time; sleep duration; sleep efficiency | Research grade | Primary Endpoint | 52 weeks | NR | NR |
| Wheeler, A. P. et al. 2025 | Physical activity tracker (exact model reported in article methods/supplement if available) | Physical activity level / activity-tracker data | Consumer/research grade unclear | Disease monitoring | up to 115 weeks | NR | Physical activity assessed from enrolment up to maximum 115 weeks |
| Kumar, A. et al. 2026 | NR | Step count / physical activity | Consumer grade / model NR | Secondary Endpoint | NR | NR | NR |
| Saito, T. et al. 2025 | Duranta wearable non-invasive wireless patch ECG system | Continuous ECG; atrial fibrillation detection; AF event number and duration | Medical/clinical grade | Primary Endpoint | 7 days | NR | Primary AF-detection outcome assessed while wearing Duranta for 7 days |
| Shakya, S. et al. 2026 | Device model NR in accessible article text | Sedentary behavior; light PA; MVPA; daily step count | Research grade | Disease monitoring | NR | NR | NR |
| Alcoceba-Herrero, I. et al. 2024 | Bakeey E66 smartwatch + pulse oximeter | Heart rate; SpO₂; respiratory rate; body temperature; alert burden | Consumer grade / clinical monitoring combination | Disease monitoring | NR | NR | NR |
| Solk, P. et al. 2025 | Fitbit (core intervention; exact model NR in this paper) | Fitbit wear/adherence; MVPA goal adherence; physical activity engagement | Consumer grade | Disease monitoring | 12 weeks | NR | Fitbit-based intervention lasted 12 weeks |
| Barua, S. et al. 2025 | Fitbit Inspire | Wear adherence/valid days; daily steps; accelerometer-derived activity patterns | Consumer grade | Secondary Endpoint | 3 months | NR | Participants instructed to wear Fitbit for the 3-month study; average wear 76 days |
| Bruna, C. L. et al. 2026 | Fitbit Inspire 2 | Step count; active minutes; heart rate variability (HRV) | Consumer grade | Primary Endpoint | 90 days | NR | Postoperative physical activity assessed during first 90 days |
| Sun, V. et al. 2025 | Wristband accelerometer; exact model NR in accessible article extract | Daily step counts / physical activity and functional recovery signals | Research/clinical monitoring grade | Disease monitoring | NR | NR | NR |
| Rethorst, C. D. et al. 2024 | ActiGraph GT3X+; Fitbit Alta HR | Moderate-to-vigorous physical activity (MVPA); light-intensity physical activity (LPA) | Research grade outcome device + consumer grade intervention device | Primary Endpoint | NR | NR | NR |
| Abdelhamid, K. et al. 2025 | Corsano Health CardioWatch 287 Bracelet (CardioWatch 287-1B) | ECG/rhythm/AF burden | Medical/clinical grade — verify regulatory status | Disease monitoring | NR | NR | NR |
| Rees-Punia, E. et al. 2025 | Research accelerometer; exact model NR in accessible indexed text | MVPA; sedentary time | Research grade | Primary Endpoint | NR | NR | NR |
| van Steijn, N. J. et al. 2026 | Apple Watch | Photoplethysmography irregular-rhythm screening; single-lead ECG; confirmed new-onset atrial fibrillation | Consumer grade | Primary Endpoint | NR | NR | NR |
| Lim, J. S. et al. 2025 | FITMETER, 32 Hz triaxial accelerometer | MVPA duration; physical activity adherence | Research grade | Secondary Endpoint | NR | NR | NR |
| Chen, Y. et al. 2025 | Huawei smartwatch; exact model NR | HRV; respiratory rate; SpO₂; cough-derived audio features | Consumer grade | Disease monitoring | NR | NR | NR |
| Kókai, L. L. et al. 2025 | Fitbit Inspire 2 | Moderate-to-vigorous physical activity (MVPA) | Consumer grade | Primary Endpoint | NR | NR | NR |
| Lödding, P. et al. 2024 | Consumer activity tracker; | Daily step count; activity-tracker adherence | Consumer grade | Secondary Endpoint | 12 months | NR | Daily step tracking during 12-month telemonitoring-supported PA intervention |
| Engdal, M. et al. 2024 | activPAL (model NR) | Total walking duration; maximum steps within a walking bout; sit-to-stand-to-walk transfers | Research grade | Primary Endpoint | NR | NR | NR |
| Zwiers, L. C. et al. 2025 | Ava bracelet | Wearable physiological parameters used by infection algorithm (multisensor physiology) plus symptom data; infection alerts | Medical device | Primary Endpoint | NR | NR | NR |
| Yarnall, A. J. et al. 2026 | Mobilise-D single wearable sensor | 24 validated digital mobility outcomes: walking duration; steps; cadence; walking-bout number/pattern; gait/mobility performance | Research grade | Primary Endpoint | NR | NR | NR |
| Jones, T. et al. 2026 | ActiGraph GT3X+; Polar OH1 | Raw triaxial acceleration (30 Hz); sedentary time; light physical activity; walking; running; moderate-to-vigorous physical activity (MVPA); ENMO used to classify walking intensity; continuous heart rate during ~25% of supervised sessions | Research grade (ActiGraph); consumer/research exercise HR sensor (Polar OH1) | Secondary Endpoint | 7 days at 4 time points | Specific periods | ActiGraph worn for 7 days at baseline, post-intervention, 6 and 12 months |
| Khairat, S. et al. 2026 | Fitbit activity tracker; exact model NR | Heart rate; step count; active minutes; calories burned. Analytical wearable outcomes in this report: daily step count and daily walking distance, including 7-day moving averages. | Consumer grade | Secondary Endpoint | NR / parent intervention longitudinal | Continuous | Daily Fitbit data with 7-day moving averages; fixed overall wear window not clearly reported |
| Khetrapal, P. et al. 2024 | Misfit Shine (Misfit; Fossil Group Inc.) | Step counts: mean/average daily steps, maximum steps in a single day, and total weekly steps (for participants with 7 consecutive days of data) | Consumer grade | Secondary Endpoint | 7-day periods at multiple time points | Specific periods | 7-day wear periods at baseline and postoperative day 5, weeks 5, 12 and 26 |
| Kosik-Rose, E. et al. 2026 | Empatica EmbracePlus | PPG-derived pulse rate; accelerometer-derived motor activity/activity counts; device wear percentage. Daily derived measures: 24-h, daytime (09:00–21:00), and nighttime (01:00–06:00) average pulse rate and motor activity. | Research/clinical grade | Disease monitoring | 2 weeks | Specific periods | Empatica smartwatch data collected for 2 weeks at baseline |
| Krell, V. et al. 2024 | Fitbit Charge 2 | Steps; total active minutes; light-, moderate- and vigorous-intensity active minutes; resting heart rate; total and active calories burned; exercise sessions/heart-rate ranges were also recorded for intervention monitoring | Consumer grade | Secondary Endpoint | NR | Specific periods | NR |
| Lee, J. et al. 2024 | Fitbit Versa, version 1 (intervention/device A); Fitbit Flex, version 1 (outcome measurement/device B) | Daily steps; physical-activity intensity; activity time; activity frequency; moderate-to-vigorous physical activity (MVPA) time; heart rate during exercise | Consumer grade | Primary Endpoint | 6 months | Specific periods | NR |
| Leps, C. et al. 2025 | Garmin vívoactive 4 and vívoactive 4s | Daily steps; moderate physical activity (MPA) minutes/week; vigorous physical activity (VPA) minutes/week; moderate-to-vigorous physical activity (MVPA); heart-rate-derived activity intensity | Consumer grade | Primary Endpoint | 6 months | Continuous | Continuous device-based physical activity monitoring over six months after surgery |
| Li, G. et al. 2025 | Fitbit Charge (generation not specified) | Daily step count (steps/day); sleep duration (minutes/night) | Consumer grade | Primary Endpoint | 84 days | Continuous | Fitbit Charge continuously measured steps and sleep for 84 days |
| Liu, C. et al. 2026 | XA-5 accelerometer | Objective sleep: total sleep time, sleep efficiency, awakening time/nocturnal wakefulness; daytime physical activity and physiological signals were also recorded | Research grade | Primary Endpoint | NR | Specific periods | NR |
| Liu, D. et al. 2024 | Fitbit Alta HR | Total sleep time; REM sleep time; light sleep time; deep sleep time; awake time; number of awakenings; sleep fragmentation index; sleep efficiency. The device combines wrist actigraphy with PPG/heart-rate sensing for its sleep algorithm, but detailed HR data were not recorded for analysis. | Consumer grade | Primary Endpoint | NR | Specific periods | NR |
| Mura, M. et al. 2025 | Fitbit Alta HR | Daily step count | Consumer grade | Primary Endpoint | 6 months intervention; controls 14 days ×2 | Specific periods | Intervention wore Fitbit throughout 6 months; controls first and last 14 days |
| Page, L. L. et al. 2024 | Fitbit Inspire HR; Polar H10 chest strap comparator | Heart rate (beats/min) measured concurrently at pre-exercise rest, warm-up, midpoint of moderate-intensity exercise, cool-down, and postexercise recovery | Consumer grade (Fitbit Inspire HR); Polar H10 used as validated criterion/comparator | Primary Endpoint | 12-week exercise programme | Specific periods | HR measured during 60-min supervised exercise sessions, 3 sessions/week across 12 weeks; 200 sessions analysed |
| Ptomey, L. T. et al. 2026 | ActiGraph wGT3X-BT; Fitbit Versa Lite | ActiGraph: moderate-to-vigorous physical activity (MVPA), sedentary time, light/moderate/vigorous activity from accelerometer counts. Fitbit: physical-activity self-monitoring/adherence (valid wear days) and activity feedback. COSMED K5 was additionally used in a volunteer subsample to quantify exercise-session METs/MVPA but was not the main free-living wearable outcome device. | Research grade (ActiGraph wGT3X-BT); consumer grade (Fitbit Versa Lite) | Primary Endpoint | 6 months Fitbit; ActiGraph 7 days at baseline/3/6 months | Continuous | Fitbit throughout intervention; ActiGraph 7-day objective assessments |
| Torres-Lopez, L. V. et al. 2024 | ActiGraph GT3X+; POLAR RS300X | ActiGraph-derived sleep timing and duration/quality: total sleep time, total time in bed, sleep efficiency, wake after sleep onset (WASO); raw acceleration/physical-activity pattern. POLAR RS300X: exercise heart rate/intensity during intervention sessions. | Research grade (ActiGraph GT3X+); consumer/research exercise HR monitor (POLAR RS300X) | Secondary Endpoint | 20 weeks | Specific periods | NR |
| Zimniak, L. et al. 2026 | Samsung Galaxy Watch Active 2, 44-mm graphite; paired with Xiaomi Redmi Note 3 smartphone and Samsung Health application | Daily step count; cumulative step count over postoperative days 0–5. The smartwatch was described as capable of monitoring other physiological/activity parameters, but step count was the wearable-derived variable analysed in this report. | Consumer grade | Primary Endpoint | Postoperative days 0–5 | Continuous | Cumulative wearable steps recorded from POD0 through POD5 |
| Rosenfeldt, A. B. et al. 2026 | Garmin vívofit 4 Fitness Activity Tracker; Garmin Connect mobile application. Peloton stationary cycle used for the aerobic exercise intervention. | Total physical activity expressed as steps/day. The Garmin captured ambulatory steps and cycling activity under a single physical-activity metric. Daily step count was the wearable-derived variable analysed. | Consumer grade | Secondary Endpoint | 12 months | Continuous | Garmin vívofit 4 worn throughout 12 months |
| Seifert, M. et al. 2024 | POLAR A370 fitness watch (Polar, Kempele, Finland) | Daily step count and walking distance. Tracker continuously recorded physical activity; study analysed steps/day at 2 weeks, 6 months and 12 months and walking distance/week. | Consumer grade | Primary Endpoint | 12 months | Continuous | POLAR A370 worn daily during daytime for 12 months |
| Vetrovsky, T. et al. 2026 | Garmin vívofit; ActiGraph wGT3X-BT | Daily steps; MVPA / sustained physical activity | Consumer grade + research grade | Disease monitoring | 12 months | Continuous | 12-month follow-up of WATCHFUL trial with device-measured physical activity |

**Table S3. Digital measures captured and their use in included studies**

| **Study** | **Activity** | **Heart rate / HRV** | **SpO₂** | **Sleep** | **Respiratory rate** | **Skin temperature** | **Other signals** | **Use of digital measures** |
| --- | --- | --- | --- | --- | --- | --- | --- | --- |
| Andellini 2024 | – | HRV (ECG-derived) | – | – | – | – | ECG morphology | Monitoring (hypoglycaemia detection) |
| Antikainen 2022 | Steps | HR, HRR, RR intervals | – | Sleep | RR | Skin temperature | – | Monitoring (fatigue, sleep) |
| Berry 2023 | Steps/activity | – | – | Sleep duration/quality | – | – | – | Secondary outcomes |
| Chen 2023 | Steps, ambulatory time | – | – | Sleep stages | – | – | Electrodermal activity | Outcome assessment |
| Creagh 2024 | Activity patterns | – | – | Sleep | – | – | – | Disease monitoring (RA severity) |
| Deschler-Baier 2024 | Steps | Resting HR | – | – | – | – | – | Outcome assessment (treatment response) |
| Figueiro 2020 | Rest-activity rhythms | – | – | Sleep metrics | – | – | – | Outcome assessment |
| Friesner 2024 | Step count decline | – | – | – | – | – | – | Outcome prediction (hospitalisation) |
| Garcia 2023 | – | Nocturnal HR | – | – | – | – | – | Disease monitoring (CV risk) |
| Garland 2023 | – | HRV | – | – | – | – | – | Outcome assessment |
| Gashi 2024 | Steps, activity intensity | HR, HRV | SpO₂ | Sleep | RR | Skin temperature | Electrodermal activity | Disease monitoring (MS) |
| Gepner 2022 | – | HR, HRV | SpO₂ | – | RR | Skin temperature | Blood pressure | Monitoring (vaccine response) |
| Golbus 2021 | Steps, distance | HR | – | – | – | – | Blood pressure | Descriptive monitoring |
| Golbus 2023 | Steps | – | – | – | – | – | – | Outcome assessment |
| Gupta 2023 | Limb movement | – | – | – | – | – | – | Disease monitoring (ALS progression) |
| Hauguel-Moreau 2020 | Steps | – | – | – | – | – | – | Outcome assessment |
| Hill 2020 | Steps | – | – | – | – | – | – | Outcome assessment |
| Jaiswal 2021 | – | – | – | Sleep fragmentation | – | – | – | Outcome Prediction (delirium) |
| Kanczok 2024 | Steps | – | – | – | – | – | – | Outcome assessment |
| Kimura 2023 | Activity levels | HR | – | Sleep | – | Temperature | – | Disease monitoring (amyloid prediction) |
| Kruizinga 2021 | Steps | HR | – | – | – | – | – | Monitoring (recovery) |
| Lönfeldt 2023 | – | HR | – | – | – | Skin temp | Electrodermal activity | Disease monitoring (OCD events) |
| Levi 2024 | – | HR, HRV | – | – | – | – | – | Monitoring (vaccine side effects) |
| Li 2021 | Steps | HR | SpO₂ | Sleep | – | – | – | Monitoring (lockdown effects) |
| Lim 2023 | Activity composition | – | – | Sleep | – | – | – | Outcome assessment |
| Lin 2024 | Steps | HR, HRV | – | – | – | – | – | Outcome prediction (CBT response) |
| Lipner 2023 | Steps | – | – | – | – | – | – | Disease monitoring (pain/activity) |
| Lubitz 2022 | – | HR, RR intervals | – | – | – | – | ECG rhythm detection | Disease monitoring (AF detection) |
| Maurice-Szamburski 2022 | Activity time | – | – | Sleep | – | – | – | Outcome assessment |
| McCausland 2023 | – | HR | – | – | – | – | ECG | Disease monitoring (arrhythmia) |
| Padron 2022 | – | – | – | Sleep efficiency | – | – | – | Outcome assessment |
| Patel 2023 | Steps | – | – | Sleep | – | – | – | Outcome prediction (readmission) |
| Richards 2020 | Steps | – | – | – | – | – | – | Outcome prediction |
| Risch 2022 | – | HR, HRV | – | – | RR | Skin temperature | – | Disease monitoring (COVID detection) |
| Rutkove 2020 | Activity | – | – | – | – | – | – | Outcome assessment |
| Rykov 2024 | – | HRV | – | – | – | Skin temp | Electrodermal activity | Outcome prediction |
| Schalkamp 2023 | Activity | – | – | Sleep | – | – | – | Disease monitoring (Parkinson’s) |
| Sindorf 2024 | Activity | – | – | Sleep | – | – | – | Outcome assessment |
| Temple 2023 | – | HR | SpO₂ | – | – | – | ECG | Outcome assessment (infection detection) |
| Umbricht 2020 | Activity states | – | – | – | – | – | – | Disease monitoring (schizophrenia) |
| Van den Eynde 2023 | Activity | HR | – | – | RR | – | – | Disease monitoring (treatment response) |
| Wang 2021 | Activity | – | – | Sleep | – | – | – | Outcome assessment |
| Woelfle 2023 | Steps | HR | – | Sleep (deep) | – | – | – | Outcome assessment |
| Wood 2023 | Steps, activity | HR | – | Sleep | – | – | – | Outcome assessment |
| Xu 2024 | Activity | HR | – | Sleep | – | – | – | Disease monitoring (MCI prediction) |
| Yamagami 2021 | – | HR | SpO₂ | – | – | – | – | Disease monitoring (COVID worsening) |
| Yang 2021 | Activity | – | – | – | – | – | – | Outcome prediction (survival) |
| Yang 2024 | Activity | – | – | – | – | – | – | Disease monitoring (CV recovery) |
| Soman, P. 2025 | 6-minute walk distance (6MWD) | – | – | – | – | – | – | Disease monitoring |
| Kilic-Berkmen, G. 2024 | Blink/eyelid movement-derived blepharospasm measures | – | – | – | – | – | Blink/eyelid movement-derived blepharospasm measures | Disease monitoring |
| Carrillo, B. J. P. 2024 | Steps/day; heart rate; sleep quality; continuous glucose | Steps/day; heart rate; sleep quality; continuous glucose | – | Steps/day; heart rate; sleep quality; continuous glucose | – | – | Steps/day; heart rate; sleep quality; continuous glucose | Secondary Endpoint |
| Hamm-Faber, T. E. 2025 | Steps/day; distance/day; strenuous physical activity; body position/mobility | – | – | – | – | – | – | Disease monitoring |
| Kwasnicki, R. M. 2024 | Physical activity / functional mobility relative to preoperative baseline | – | – | – | – | – | – | Primary Endpoint |
| Vicenzi, M. 2026 | – | – | Minute ventilation; respiratory frequency; tidal volume; ventilatory equivalent for CO2; oxygen uptake during 6MWT | – | Minute ventilation; respiratory frequency; tidal volume; ventilatory equivalent for CO2; oxygen uptake during 6MWT | – | Minute ventilation; respiratory frequency; tidal volume; ventilatory equivalent for CO2; oxygen uptake during 6MWT | Secondary Endpoint |
| Ibañez-Micó, S. 2024 | Physical activity / weekly exercise adherence | – | – | – | – | – | – | Secondary Endpoint |
| Martinique, V. B. 2026 | Inactivity; light/moderate/vigorous activity; total PA; step count; ENMO acceleration | – | – | – | – | – | – | Primary Endpoint |
| Stein, M. 2026 | Wearable activity/physiological signals combined with PROMs and patient-performed measures | – | – | – | – | – | – | Disease monitoring |
| Jackson, J. L. 2025 | Moderate-to-vigorous physical activity (MVPA), min/day | – | – | – | – | – | – | Primary Endpoint |
| Zhang, P. 2026 | – | Heart rate; HRV including RMSSD; exercise intensity / heart-rate reserve | – | – | – | – | – | Secondary Endpoint |
| Adasuriya, G. 2024 | – | – | – | – | – | – | ECG/rhythm/AF burden | Disease monitoring |
| Wu, C. Y. 2025 | Daily total step count; average daily steps over final 14 days of each block; wear adherence; percent change in steps from baseline | – | – | – | – | – | – | Disease monitoring |
| Li, H. 2024 | Exercise heart rate/intensity; physical activity energy expenditure (PAEE); exercise adherence | Exercise heart rate/intensity; physical activity energy expenditure (PAEE); exercise adherence | – | – | – | – | – | Disease monitoring |
| Brakenridge, C. J. 2026 | Steps; sedentary time; light PA; MVPA; heart-rate-derived MVPA | – | – | – | – | – | – | Secondary Endpoint |
| Moon, H. M. 2026 | Exercise heart rate / maximum HR / walking HR | Exercise heart rate / maximum HR / walking HR | – | – | – | – | – | Secondary Endpoint |
| Li, G. 2025 | Exercise duration; effective exercise duration; exercise prescription adherence / activity | – | – | – | – | – | – | Primary Endpoint |
| Schrauben, S. J. 2024 | Daily step counts; step-count adherence | – | – | – | – | – | – | Primary Endpoint |
| Glavas, C. 2024 | Sedentary time; light PA; moderate PA; vigorous PA; very vigorous PA; MVPA; steps/day | – | – | – | – | – | – | Secondary Endpoint |
| Gunturkun, F. 2025 | Physical activity; steps; heart rate; resting/walking HR; HRV; SpO₂; respiratory rate; ECG; blood pressure platform data | Physical activity; steps; heart rate; resting/walking HR; HRV; SpO₂; respiratory rate; ECG; blood pressure platform data | Physical activity; steps; heart rate; resting/walking HR; HRV; SpO₂; respiratory rate; ECG; blood pressure platform data | – | Physical activity; steps; heart rate; resting/walking HR; HRV; SpO₂; respiratory rate; ECG; blood pressure platform data | – | Physical activity; steps; heart rate; resting/walking HR; HRV; SpO₂; respiratory rate; ECG; blood pressure platform data | Secondary Endpoint |
| Nishio, R. 2025 | Heart rate; exercise/physical activity metrics; remote exercise adherence | Heart rate; exercise/physical activity metrics; remote exercise adherence | – | – | – | – | – | Secondary Endpoint |
| Gill, S. K. 2024 | Heart rate; step count/physical activity | Heart rate; step count/physical activity | – | – | – | – | – | Primary Endpoint |
| Sunaga, A. 2026 | – | – | – | – | – | – | AF/rhythm recurrence detected by Apple Watch; ECG/rhythm notifications | Disease monitoring |
| Jo, Y. 2025 | Steps; distance; heart rate; activity intensity; derived HAM and excessive sedentary heart rate (eSHR) | Steps; distance; heart rate; activity intensity; derived HAM and excessive sedentary heart rate (eSHR) | – | – | – | – | – | Disease monitoring |
| Hartung, V. 2025 | Steps/day; device-measured MVPA minutes | – | – | – | – | – | – | Primary Endpoint |
| Kaur, H. 2026 | Moderate-to-vigorous physical activity (MVPA) | – | – | – | – | – | – | Secondary Endpoint |
| Devasahayam, A. J. 2025 | Step count; heart rate; resting heart rate; time in moderate-to-vigorous HR range; time in vigorous HR range; adherence to physical activity guidelines | Step count; heart rate; resting heart rate; time in moderate-to-vigorous HR range; time in vigorous HR range; adherence to physical activity guidelines | – | – | – | – | – | Primary Endpoint |
| Pinto, B. M. 2025 | Bouted MVPA; total MVPA | – | – | – | – | – | – | Primary Endpoint |
| Li, H. 2024 | – | – | – | Total sleep time; WASO; number of awakenings; time in bed; sleep efficiency | – | – | – | Disease monitoring |
| Han, X. 2026 | – | – | – | Total sleep time; deep sleep time; light sleep time; REM sleep time; number of awakenings | – | – | – | Primary Endpoint |
| Rillamas-Sun, E. 2026 | Vigorous PA; MVPA; sedentary time; Fitbit physical activity | – | – | – | – | – | – | Disease monitoring |
| Simpson, L. A. 2025 | – | – | – | – | – | – | – | Primary Endpoint |
| Hoffman, R. M. 2025 | Percentage of active wear time in sedentary activity | – | – | – | – | – | – | Secondary Endpoint |
| Montagne, B. 2026 | – | – | – | Total sleep time; REM sleep duration; night-to-night REM variability; other sleep architecture measures | – | – | – | Disease monitoring |
| Liu, C. 2026 | – | – | – | Total sleep time; sleep efficiency; wake after sleep onset; deep/light/REM sleep | – | – | – | Primary Endpoint |
| Hardcastle, S. J. 2024 | MVPA; moderate PA; light PA; sedentary time; bouted MVPA; wear time | – | – | – | – | – | – | Primary Endpoint |
| Rantula, O. A. 2025 | – | – | – | – | – | – | PPG rhythm; AF detection; AF burden; AF-to-sinus-rhythm change | Disease monitoring |
| Pu, J. 2025 | – | – | – | Deep/light/REM sleep proportions; awakenings; sleep continuity; sleep respiratory quality | – | – | – | Secondary Endpoint |
| Holdom, C. J. 2025 | Accelerometry-derived activity outcomes including peak 6-min activity and longitudinal activity measures | – | – | – | – | – | – | Disease monitoring |
| Stein, R. G. 2025 | MVPA; light PA; sleep efficiency; sleep latency; sleep duration | – | – | MVPA; light PA; sleep efficiency; sleep latency; sleep duration | – | – | – | Disease monitoring |
| Alnes, S. R. 2026 | Daily steps; heart rate; weekly intensity minutes | Daily steps; heart rate; weekly intensity minutes | – | – | – | – | – | Secondary Endpoint |
| Lachant, D. J. 2026 | Peak 5-min steps; daily steps; moderate activity time; vigorous activity time | – | – | – | – | – | – | Secondary Endpoint |
| Schäfer, L. 2026 | – | – | – | – | – | Continuous axillary body temperature; fever ≥38.0°C; fever timing/duration | – | Primary Endpoint |
| Werner, C. 2026 | Walking amount; walking-bout pattern; walking speed; stride length; 90th-percentile walking speed and other digital mobility outcomes | – | – | – | – | – | – | Secondary Endpoint |
| Murray, R. M. 2025 | Device-measured MVPA; light PA | – | – | – | – | – | – | Secondary Endpoint |
| Mohapatra, S. 2025 | Daily steps; tracker adherence | – | – | – | – | – | – | Primary Endpoint |
| Greysen, S. R. 2022 | Daily steps; sleep duration | – | – | Daily steps; sleep duration | – | – | – | Disease monitoring |
| Li, G. 2025 | Daily physical activity time; daily awake sedentary time; sitting/walking habits | – | – | – | – | – | – | Primary Endpoint |
| Dickerson, S. S. 2026 | – | – | – | Sleep onset latency; wake after sleep onset; total sleep time; sleep efficiency | – | – | – | Secondary Endpoint |
| Wang, H. 2025 | – | – | – | – | – | – | – | Disease monitoring |
| Blondeel, A. 2025 | Steps/day; objectively measured physical activity; Fitbit daily step count | – | – | – | – | – | – | Primary Endpoint |
| van Vliet, M. 2025 | – | – | – | – | – | – | PPG rhythm; single-lead ECG; AF detection; AF prevalence; AF burden | Disease monitoring |
| Zhu, B. 2025 | – | ECG; heart rate; SpO₂; non-invasive blood pressure; physiological alarms | ECG; heart rate; SpO₂; non-invasive blood pressure; physiological alarms | – | – | – | ECG; heart rate; SpO₂; non-invasive blood pressure; physiological alarms | Secondary Endpoint |
| Vetsch, T. 2025 | Steps/day; raw acceleration metrics; physical activity | – | – | – | – | – | – | Secondary Endpoint |
| Ahluwalia, N. 2026 | – | PPG-based heart rate/rhythm monitoring; single-lead ECG; AF recurrence/rhythm detection | – | – | – | – | PPG-based heart rate/rhythm monitoring; single-lead ECG; AF recurrence/rhythm detection | Primary Endpoint |
| Dong, Y. 2024 | – | – | – | Sleep structure: nocturnal wakefulness, deep sleep and other sleep-duration/stage measures | – | – | – | Secondary Endpoint |
| Flick, M. 2026 | – | SpO₂; respiratory rate; systolic blood pressure; pulse/heart rate | SpO₂; respiratory rate; systolic blood pressure; pulse/heart rate | – | SpO₂; respiratory rate; systolic blood pressure; pulse/heart rate | – | SpO₂; respiratory rate; systolic blood pressure; pulse/heart rate | Primary Endpoint |
| Küng, P. 2025 | Daily physical activity | – | – | – | – | – | – | Primary Endpoint |
| Vetrovsky, T. 2024 | Daily step count; moderate-to-vigorous physical activity (MVPA) | – | – | – | – | – | – | Secondary Endpoint |
| Docherty, K. F. 2024 | Steps; light-to-vigorous PA; MVPA; walking movement intensity; vector magnitude units; total activity counts | – | – | – | – | – | – | Primary Endpoint |
| Wagnild, J. M. 2025 | Total activity counts; MVPA; light PA; sedentary time; sleep duration; sleep efficiency | – | – | Total activity counts; MVPA; light PA; sedentary time; sleep duration; sleep efficiency | – | – | – | Primary Endpoint |
| Wheeler, A. P. 2025 | Physical activity level / activity-tracker data | – | – | – | – | – | – | Disease monitoring |
| Kumar, A. 2026 | Step count / physical activity | – | – | – | – | – | – | Secondary Endpoint |
| Saito, T. 2025 | – | – | – | – | – | – | Continuous ECG; atrial fibrillation detection; AF event number and duration | Primary Endpoint |
| Shakya, S. 2026 | Sedentary behavior; light PA; MVPA; daily step count | – | – | – | – | – | – | Disease monitoring |
| Alcoceba-Herrero, I. 2024 | – | Heart rate; SpO₂; respiratory rate; body temperature; alert burden | Heart rate; SpO₂; respiratory rate; body temperature; alert burden | – | Heart rate; SpO₂; respiratory rate; body temperature; alert burden | Heart rate; SpO₂; respiratory rate; body temperature; alert burden | – | Disease monitoring |
| Solk, P. 2025 | Fitbit wear/adherence; MVPA goal adherence; physical activity engagement | – | – | – | – | – | – | Disease monitoring |
| Barua, S. 2025 | Wear adherence/valid days; daily steps; accelerometer-derived activity patterns | – | – | – | – | – | – | Secondary Endpoint |
| Bruna, C. L. 2026 | Step count; active minutes; heart rate variability (HRV) | Step count; active minutes; heart rate variability (HRV) | – | – | – | – | – | Primary Endpoint |
| Sun, V. 2025 | Daily step counts / physical activity and functional recovery signals | – | – | – | – | – | – | Disease monitoring |
| Rethorst, C. D. 2024 | Moderate-to-vigorous physical activity (MVPA); light-intensity physical activity (LPA) | – | – | – | – | – | – | Primary Endpoint |
| Abdelhamid, K. 2025 | – | – | – | – | – | – | ECG/rhythm/AF burden | Disease monitoring |
| Rees-Punia, E. 2025 | MVPA; sedentary time | – | – | – | – | – | – | Primary Endpoint |
| van Steijn, N. J. 2026 | – | Photoplethysmography irregular-rhythm screening; single-lead ECG; confirmed new-onset atrial fibrillation | – | – | – | – | Photoplethysmography irregular-rhythm screening; single-lead ECG; confirmed new-onset atrial fibrillation | Primary Endpoint |
| Lim, J. S. 2025 | MVPA duration; physical activity adherence | – | – | – | – | – | – | Secondary Endpoint |
| Chen, Y. 2025 | – | HRV; respiratory rate; SpO₂; cough-derived audio features | HRV; respiratory rate; SpO₂; cough-derived audio features | – | HRV; respiratory rate; SpO₂; cough-derived audio features | – | HRV; respiratory rate; SpO₂; cough-derived audio features | Disease monitoring |
| Kókai, L. L. 2025 | Moderate-to-vigorous physical activity (MVPA) | – | – | – | – | – | – | Primary Endpoint |
| Lödding, P. 2024 | Daily step count; activity-tracker adherence | – | – | – | – | – | – | Secondary Endpoint |
| Engdal, M. 2024 | Total walking duration; maximum steps within a walking bout; sit-to-stand-to-walk transfers | – | – | – | – | – | – | Primary Endpoint |
| Zwiers, L. C. 2025 | – | – | – | – | – | – | – | Primary Endpoint |
| Yarnall, A. J. 2026 | 24 validated digital mobility outcomes: walking duration; steps; cadence; walking-bout number/pattern; gait/mobility performance | – | – | – | – | – | – | Primary Endpoint |
| Jones, T. 2026 | Raw triaxial acceleration (30 Hz); sedentary time; light physical activity; walking; running; moderate-to-vigorous physical activity (MVPA); ENMO used to classify walking intensity; continuous heart rate during ~25% of supervised sessions | Raw triaxial acceleration (30 Hz); sedentary time; light physical activity; walking; running; moderate-to-vigorous physical activity (MVPA); ENMO used to classify walking intensity; continuous heart rate during ~25% of supervised sessions | – | – | – | – | – | Secondary Endpoint |
| Khairat, S. 2026 | Heart rate; step count; active minutes; calories burned. Analytical wearable outcomes in this report: daily step count and daily walking distance, including 7-day moving averages. | Heart rate; step count; active minutes; calories burned. Analytical wearable outcomes in this report: daily step count and daily walking distance, including 7-day moving averages. | – | – | – | – | – | Secondary Endpoint |
| Khetrapal, P. 2024 | Step counts: mean/average daily steps, maximum steps in a single day, and total weekly steps (for participants with 7 consecutive days of data) | – | – | – | – | – | – | Secondary Endpoint |
| Kosik-Rose, E. 2026 | PPG-derived pulse rate; accelerometer-derived motor activity/activity counts; device wear percentage. Daily derived measures: 24-h, daytime (09:00–21:00), and nighttime (01:00–06:00) average pulse rate and motor activity. | PPG-derived pulse rate; accelerometer-derived motor activity/activity counts; device wear percentage. Daily derived measures: 24-h, daytime (09:00–21:00), and nighttime (01:00–06:00) average pulse rate and motor activity. | – | – | – | – | – | Disease monitoring |
| Krell, V. 2024 | Steps; total active minutes; light-, moderate- and vigorous-intensity active minutes; resting heart rate; total and active calories burned; exercise sessions/heart-rate ranges were also recorded for intervention monitoring | Steps; total active minutes; light-, moderate- and vigorous-intensity active minutes; resting heart rate; total and active calories burned; exercise sessions/heart-rate ranges were also recorded for intervention monitoring | – | – | – | – | – | Secondary Endpoint |
| Lee, J. 2024 | Daily steps; physical-activity intensity; activity time; activity frequency; moderate-to-vigorous physical activity (MVPA) time; heart rate during exercise | Daily steps; physical-activity intensity; activity time; activity frequency; moderate-to-vigorous physical activity (MVPA) time; heart rate during exercise | – | – | – | – | – | Primary Endpoint |
| Leps, C. 2025 | Daily steps; moderate physical activity (MPA) minutes/week; vigorous physical activity (VPA) minutes/week; moderate-to-vigorous physical activity (MVPA); heart-rate-derived activity intensity | – | – | – | – | – | – | Primary Endpoint |
| Li, G. 2025 | Daily step count (steps/day); sleep duration (minutes/night) | – | – | Daily step count (steps/day); sleep duration (minutes/night) | – | – | – | Primary Endpoint |
| Liu, C. 2026 | Objective sleep: total sleep time, sleep efficiency, awakening time/nocturnal wakefulness; daytime physical activity and physiological signals were also recorded | – | – | Objective sleep: total sleep time, sleep efficiency, awakening time/nocturnal wakefulness; daytime physical activity and physiological signals were also recorded | – | – | – | Primary Endpoint |
| Liu, D. 2024 | – | – | – | Total sleep time; REM sleep time; light sleep time; deep sleep time; awake time; number of awakenings; sleep fragmentation index; sleep efficiency. The device combines wrist actigraphy with PPG/heart-rate sensing for its sleep algorithm, but detailed HR data were not recorded for analysis. | – | – | – | Primary Endpoint |
| Mura, M. 2025 | Daily step count | – | – | – | – | – | – | Primary Endpoint |
| Page, L. L. 2024 | – | Heart rate (beats/min) measured concurrently at pre-exercise rest, warm-up, midpoint of moderate-intensity exercise, cool-down, and postexercise recovery | – | – | – | – | – | Primary Endpoint |
| Ptomey, L. T. 2026 | ActiGraph: moderate-to-vigorous physical activity (MVPA), sedentary time, light/moderate/vigorous activity from accelerometer counts. Fitbit: physical-activity self-monitoring/adherence (valid wear days) and activity feedback. COSMED K5 was additionally used in a volunteer subsample to quantify exercise-session METs/MVPA but was not the main free-living wearable outcome device. | – | – | – | – | – | – | Primary Endpoint |
| Torres-Lopez, L. V. 2024 | ActiGraph-derived sleep timing and duration/quality: total sleep time, total time in bed, sleep efficiency, wake after sleep onset (WASO); raw acceleration/physical-activity pattern. POLAR RS300X: exercise heart rate/intensity during intervention sessions. | ActiGraph-derived sleep timing and duration/quality: total sleep time, total time in bed, sleep efficiency, wake after sleep onset (WASO); raw acceleration/physical-activity pattern. POLAR RS300X: exercise heart rate/intensity during intervention sessions. | – | ActiGraph-derived sleep timing and duration/quality: total sleep time, total time in bed, sleep efficiency, wake after sleep onset (WASO); raw acceleration/physical-activity pattern. POLAR RS300X: exercise heart rate/intensity during intervention sessions. | – | – | – | Secondary Endpoint |
| Zimniak, L. 2026 | Daily step count; cumulative step count over postoperative days 0–5. The smartwatch was described as capable of monitoring other physiological/activity parameters, but step count was the wearable-derived variable analysed in this report. | – | – | – | – | – | – | Primary Endpoint |
| Rosenfeldt, A. B. 2026 | Total physical activity expressed as steps/day. The Garmin captured ambulatory steps and cycling activity under a single physical-activity metric. Daily step count was the wearable-derived variable analysed. | – | – | – | – | – | – | Secondary Endpoint |
| Seifert, M. 2024 | Daily step count and walking distance. Tracker continuously recorded physical activity; study analysed steps/day at 2 weeks, 6 months and 12 months and walking distance/week. | – | – | – | – | – | – | Primary Endpoint |
| Vetrovsky, T. 2026 | Daily steps; MVPA / sustained physical activity | – | – | – | – | – | – | Disease monitoring |

**Table S4. Analytical methods used in included studies**

| **Study** | **Machine learning / AI** | **Regression methods** | **Statistical tests** | **Time-series / longitudinal methods** | **Other methods** |
| --- | --- | --- | --- | --- | --- |
| Andellini 2024 | – | – | Spearman correlation | – | – |
| Antikainen 2022 | – | ANCOVA | Post hoc (Tukey) | – | – |
| Berry 2023 | – | Mixed model regression | t-test, Spearman, Fisher’s exact | Autoregressive covariance structure | – |
| Chen 2023 | – | Mixed models (MMRM) | Correlation (FDR adjusted) | Repeated measures | Multiplicity correction (Benjamini–Hochberg) |
| Creagh 2024 | Deep learning (CNN), Random Forest, XGBoost | Logistic regression (elastic net, SG-lasso) | Mann–Whitney, Kruskal–Wallis | Hidden Markov model | ROC, ICC, F1 score |
| Deschler-Baier 2024 | – | – | – | – | Descriptive |
| Figueiro 2020 | – | Linear mixed-effects models | – | Longitudinal | – |
| Friesner 2024 | Neural network, Random Forest | Logistic Regression (Elastic net regression) | – | – | – |
| Garcia 2023 | – | Cox regression | – | Time-dependent modelling | Cubic splines |
| Garland 2023 | Random forest, decision tree | Logistic regression | – | – | – |
| Gashi 2024 | Machine learning classifiers/regressors, Random Forest, XGBoost and Fully Connected Neural Network | Logistic regression | Mann–Whitney, correlation | – | – |
| Gepner 2022 | – | – | t-test | Time-window comparison | Confidence intervals |
| Golbus 2021 | – | – | t-test, ANOVA | – | Descriptive statistics |
| Golbus 2023 | – | Multivariable regression | – | Longitudinal | – |
| Gupta 2023 | Machine learning models | Lasso Regression models | Mann–Whitney, correlation | Longitudinal modelling | ICC, effect size |
| Hauguel-Moreau 2020 | – | Linear regression | Chi-square | Non-linear modelling | Descriptive |
| Hill 2020 | – | Mixed models | t-test | – | – |
| Jaiswal 2021 | – | Linear regression | Mann–Whitney | – | Descriptive |
| Kanczok 2024 | – | – | Correlation, ANOVA | LOESS smoothing | Normality testing |
| Kimura 2023 | SVM, Elastic net, ML models | Logistic regression | t-test, Wilcoxon | – | – |
| Kruizinga 2021 | – | Mixed models | Correlation | Longitudinal | – |
| Lönfeldt 2023 | Neural networks, Random Forest | Logistic regression | – | Cross-validation | – |
| Levi 2024 | XGBoost, Random Forest, Multi-Layer Perceptron, K-Nearest Neighbours | – | – | Time-series smoothing | – |
| Li 2021 | – | Segmented regression | – | Interrupted time series | – |
| Lim 2023 | – | Mixed effects regression | – | Longitudinal | – |
| Lin 2024 | – | Regression | t-test, correlation | – | – |
| Lipner 2023 | – | Linear mixed models | – | Repeated measures | – |
| Lubitz 2022 | – | – | Descriptive (PPV, CI) | – | Diagnostic accuracy |
| Maurice-Szamburski 2022 | – | – | t-test, Mann–Whitney | – | Normality testing |
| McCausland 2023 | – | Generalised estimating equations | – | Longitudinal | Poisson models |
| Padron 2022 | – | Mixed linear models | Chi-square | Longitudinal | Model fit statistics |
| Patel 2023 | Machine learning models, Random Forest, Gradient Boosting | Logistic, lasso regression | – | – | AUC comparison |
| Richards 2020 | – | Logistic regression | Kruskal–Wallis, Chi-square | – | Model performance (C-index) |
| Risch 2022 | RNN (LSTM) | Mixed models | – | Time-series modelling | – |
| Rutkove 2020 | – | – | – | Longitudinal slopes | Sample size modelling |
| Rykov 2024 | Machine learning models, Elastic net regression, Random Forest ,XGBosst | Logistic regression (Elastic net) | – | Cross-validation | – |
| Schalkamp 2023 | Random Forest, Markov confusion matrix |  | – | Time-series feature extraction | – |
| Sindorf 2024 | Random Forest, XGBoost | Logistic regression | – | – | – |
| Temple 2023 | – | – | – | Multivariable process control | – |
| Umbricht 2020 | Neural network | – | Correlation | – | Classification models |
| Van den Eynde 2023 | Clustering (hierarchical) | – | – | Time-series feature extraction | – |
| Wang 2021 | – | Mixed models | Correlation | Longitudinal | – |
| Woelfle 2023 | – | Multivariable regression | Correlation | – | Feature selection |
| Wood 2023 | Clustering | Linear regression, mixed models | ANOVA | Longitudinal | – |
| Xu 2024 | Gaussian Naive Bayes, logistic regression, SVM, random forest, XGBoost, CatBoost; SHAP | Logistic regression | – | Cross-validation | SHAP analysis |
| Yamagami 2021 | – | – | Sensitivity/specificity metrics | – | Diagnostic metrics |
| Yang 2021 | Deep learning (LSTM) | – | – | Time-series modelling | – |
| Yang 2024 | XGBoost; logistic regression; naive Bayes; SVM; decision tree ensemble | Logistic regression | – | Time-series (moving averages) | Model performance metrics |
| Soman, P. 2025 | – | – | Pilot method-comparison study; mean difference; Pearson correlation; concordance correlation coefficient; Bland–Altman agreement analysis comparing sensor-derived vs observer-measured 6MWD. | – | – |
| Kilic-Berkmen, G. 2024 | – | – | – | – | Phase 2a randomized double-blind placebo-controlled exploratory analysis; between-group/within-subject outcome comparisons and power/sample-size analyses across candidate outcome measures. |
| Carrillo, B. J. P. 2024 | – | – | – | Randomized crossover comparisons of morning vs evening exercise; repeated within-participant analyses of glycaemia and wearable PA/sleep variables | – |
| Hamm-Faber, T. E. 2025 | – | – | Feasibility longitudinal pre/post analysis; correlations between objective activity measures and ODI; tests of change from baseline to 3 months. | Feasibility longitudinal pre/post analysis; correlations between objective activity measures and ODI; tests of change from baseline to 3 months. | – |
| Kwasnicki, R. M. 2024 | – | – | Prospective multicentre observational cohort; repeated pre/postoperative PA expressed relative to baseline; median/IQR trajectory and long-term paired comparison (p=0.046). | Prospective multicentre observational cohort; repeated pre/postoperative PA expressed relative to baseline; median/IQR trajectory and long-term paired comparison (p=0.046). | – |
| Vicenzi, M. 2026 | – | – | – | – | Randomized placebo-controlled trial (IMT n=12, sham n=12); baseline/post-intervention within- and between-group comparisons of inspiratory strength, 6MWD and wearable respiratory physiology. |
| Ibañez-Micó, S. 2024 | – | – | Randomized-group longitudinal analysis with ANOVA/post-hoc ANOVA; effect sizes (eta-squared); correlations between physical activity and seizure frequency. | Randomized-group longitudinal analysis with ANOVA/post-hoc ANOVA; effect sizes (eta-squared); correlations between physical activity and seizure frequency. | – |
| Martinique, V. B. 2026 | – | 10-year follow-up of randomized PFR vs TKR trial; t-tests or Wilcoxon tests comparing activity duration, total PA, steps and ENMO between groups. | 10-year follow-up of randomized PFR vs TKR trial; t-tests or Wilcoxon tests comparing activity duration, total PA, steps and ENMO between groups. | 10-year follow-up of randomized PFR vs TKR trial; t-tests or Wilcoxon tests comparing activity duration, total PA, steps and ENMO between groups. | – |
| Stein, M. 2026 | Linear ML classifier predicting MG deterioration (≥3-point QMG worsening, MG hospitalization or exacerbation); cross-validation; AUROC; evaluation across 4–10-day look-back windows and false-alarm rates. | – | – | – | – |
| Jackson, J. L. 2025 | – | – | Descriptive feasibility/acceptability statistics; group physical-activity change/effect-size estimation used to inform a larger efficacy trial. | – | – |
| Zhang, P. 2026 | – | N-of-1 randomized crossover design over 6 weeks; personalized PPG-feedback vs standardized walking periods; between-condition effect estimates with 95% CIs; HRV change and regression linking HRV improvement to subsequent PHQ-9 change. | – | – | – |
| Adasuriya, G. 2024 | – | – | – | – | PPG HR preprocessing/noise filtration; HR elevation Z-score thresholding; comparison with ILR recurrence; sensitivity, specificity, PPV, NPV and accuracy |
| Wu, C. Y. 2025 | – | – | – | – | Double-blind randomized placebo-controlled crossover trial; within-individual NR-vs-placebo comparisons; wearable step-count percent change from baseline used as exploratory outcome. |
| Li, H. 2024 | – | – | – | Two-arm randomized controlled pre/post analysis; conventional between-group comparisons of physical fitness, PAEE, symptoms, QOL and treatment adverse effects. | – |
| Brakenridge, C. J. 2026 | – | Longitudinal randomized-group comparisons and modeled activity trajectories; adjusted regression models; sensitivity analysis by Fitbit wear coverage | – | Longitudinal randomized-group comparisons and modeled activity trajectories; adjusted regression models; sensitivity analysis by Fitbit wear coverage | – |
| Moon, H. M. 2026 | – | – | Three-arm RCT; group×time repeated-measures analysis/ANOVA with F statistics and partial eta-squared; post-hoc group comparisons. | Three-arm RCT; group×time repeated-measures analysis/ANOVA with F statistics and partial eta-squared; post-hoc group comparisons. | – |
| Li, G. 2025 | – | – | Randomized controlled 5-month telerehabilitation trial; between-group pre/post comparisons of VO2peak, QOL and symptom outcomes; adherence summarized descriptively. | Randomized controlled 5-month telerehabilitation trial; between-group pre/post comparisons of VO2peak, QOL and symptom outcomes; adherence summarized descriptively. | – |
| Schrauben, S. J. 2024 | – | – | 12-week randomized crossover pilot; descriptive adoption/adherence/acceptability analyses; paired/longitudinal comparison of step counts and BP over study phases; mixed-methods end-of-study interviews. | 12-week randomized crossover pilot; descriptive adoption/adherence/acceptability analyses; paired/longitudinal comparison of step counts and BP over study phases; mixed-methods end-of-study interviews. | – |
| Glavas, C. 2024 | – | – | Pilot RCT feasibility analysis; descriptive feasibility outcomes and between-group/pre-post comparisons of secondary accelerometer-derived PA measures. | – | – |
| Gunturkun, F. 2025 | Mixed models for repeated measures for treatment-arm changes; latent class mixed models with random intercepts and natural cubic splines for longitudinal trajectory clustering; BIC/AIC model selection; absolute standardized differences; sensitivity analysis. | Mixed models for repeated measures for treatment-arm changes; latent class mixed models with random intercepts and natural cubic splines for longitudinal trajectory clustering; BIC/AIC model selection; absolute standardized differences; sensitivity analysis. | – | Mixed models for repeated measures for treatment-arm changes; latent class mixed models with random intercepts and natural cubic splines for longitudinal trajectory clustering; BIC/AIC model selection; absolute standardized differences; sensitivity analysis. | – |
| Nishio, R. 2025 | – | – | – | – | Randomized open-label pilot trial (25 online coaching, 25 wearable-only); between-group change in exercise capacity and remote rehabilitation measures. |
| Gill, S. K. 2024 | Intention-to-treat; t-test/Kruskal–Wallis; Spearman correlations; generalized linear models with random-effects for repeated HR measurements; adjusted subgroup analyses by activity; self-supervised convolutional neural network using HR/steps to predict NYHA class, evaluated with F1 score and AUROC. | – | Intention-to-treat; t-test/Kruskal–Wallis; Spearman correlations; generalized linear models with random-effects for repeated HR measurements; adjusted subgroup analyses by activity; self-supervised convolutional neural network using HR/steps to predict NYHA class, evaluated with F1 score and AUROC. | Intention-to-treat; t-test/Kruskal–Wallis; Spearman correlations; generalized linear models with random-effects for repeated HR measurements; adjusted subgroup analyses by activity; self-supervised convolutional neural network using HR/steps to predict NYHA class, evaluated with F1 score and AUROC. | – |
| Sunaga, A. 2026 | – | – | Prospective multicentre single-arm interventional trial; Kaplan–Meier estimates for death, bleeding, stroke and systemic thromboembolism; 12-month rates with Greenwood 95% CIs; chi-square/Fisher exact, t-test/Mann–Whitney/paired t-test as appropriate. | Prospective multicentre single-arm interventional trial; Kaplan–Meier estimates for death, bleeding, stroke and systemic thromboembolism; 12-month rates with Greenwood 95% CIs; chi-square/Fisher exact, t-test/Mann–Whitney/paired t-test as appropriate. | – |
| Jo, Y. 2025 | – | Multivariable Cox proportional hazards models adjusted for age, sex and treatment line; hazard ratios; Harrell concordance index (C-index) to compare prognostic models. | Multivariable Cox proportional hazards models adjusted for age, sex and treatment line; hazard ratios; Harrell concordance index (C-index) to compare prognostic models. | – | – |
| Hartung, V. 2025 | – | Intention-to-treat; Mann–Whitney U for between-group change; Wilcoxon signed-rank within groups/follow-up; Welch t-test, Fisher exact and Wilcoxon rank-sum for baseline comparisons; imputation plus available-case sensitivity analyses; effect sizes converted to Cohen d. | Intention-to-treat; Mann–Whitney U for between-group change; Wilcoxon signed-rank within groups/follow-up; Welch t-test, Fisher exact and Wilcoxon rank-sum for baseline comparisons; imputation plus available-case sensitivity analyses; effect sizes converted to Cohen d. | Intention-to-treat; Mann–Whitney U for between-group change; Wilcoxon signed-rank within groups/follow-up; Welch t-test, Fisher exact and Wilcoxon rank-sum for baseline comparisons; imputation plus available-case sensitivity analyses; effect sizes converted to Cohen d. | – |
| Kaur, H. 2026 | – | – | Secondary analysis of intervention arm; descriptive website-use statistics and Spearman partial correlations between website use and change in accelerometry-measured MVPA, diet and weight. | – | – |
| Devasahayam, A. J. 2025 | – | Multiple logistic regression with fixed effects of phase and time and random effect of site for odds of meeting PA guidelines; multiple linear regression for step count and HR-derived activity outcomes with site/time in models; multiple imputation for eligible missing PA data; Wilcoxon rank-sum for secondary questionnaire outcomes. | Multiple logistic regression with fixed effects of phase and time and random effect of site for odds of meeting PA guidelines; multiple linear regression for step count and HR-derived activity outcomes with site/time in models; multiple imputation for eligible missing PA data; Wilcoxon rank-sum for secondary questionnaire outcomes. | – | – |
| Pinto, B. M. 2025 | – | Mixed-effects regression models for between-group changes from baseline to 12 weeks; intention-to-treat RCT comparison. | – | – | – |
| Li, H. 2024 | – | Wilcoxon signed-rank tests comparing Fitbit vs Consensus Sleep Diary; Spearman rank correlations; Mann–Whitney tests by insomnia severity. | Wilcoxon signed-rank tests comparing Fitbit vs Consensus Sleep Diary; Spearman rank correlations; Mann–Whitney tests by insomnia severity. | – | – |
| Han, X. 2026 | – | Intention-to-treat; linear mixed-effects models with group, time and group×time fixed effects, participant random effect and baseline covariate; Bonferroni post-hoc tests; t-test/Mann–Whitney U; chi-square/Fisher exact. | Intention-to-treat; linear mixed-effects models with group, time and group×time fixed effects, participant random effect and baseline covariate; Bonferroni post-hoc tests; t-test/Mann–Whitney U; chi-square/Fisher exact. | – | – |
| Rillamas-Sun, E. 2026 | – | – | Pearson correlation coefficients; Bland–Altman plots; comparisons of prevalence meeting ≥150 min/week MVPA guidelines across ActiGraph, IPAQ and Fitbit. | – | – |
| Simpson, L. A. 2025 | – | – | – | Parallel-group assessor-blinded RCT; between-group comparison of average daily reach-to-grasp counts at 4 weeks with mean difference and 95% CI; secondary and within-subject tertiary analyses across post-treatment and 2-month follow-up. | – |
| Hoffman, R. M. 2025 | – | – | Secondary longitudinal analysis of parent double-blind RCT; modeled predicted change scores with simulations; participant classification by improvement in sedentary time/6MWT; independent t-tests and Fisher exact tests. | Secondary longitudinal analysis of parent double-blind RCT; modeled predicted change scores with simulations; participant classification by improvement in sedentary time/6MWT; independent t-tests and Fisher exact tests. | – |
| Montagne, B. 2026 | – | – | – | – | Cross-sectional observational comparisons by amyloid status; associations between wearable sleep characteristics and regional amyloid-PET burden plus cognitive/psychoaffective measures. |
| Liu, C. 2026 | – | Randomized double-blind controlled analysis; risk ratio for primary sleep-disturbance endpoint; between-group wearable sleep comparisons; exploratory reduced logistic regression adjusted for age and baseline PSQI. | – | – | – |
| Hardcastle, S. J. 2024 | – | Intention-to-treat randomized-group analysis of accelerometer PA outcomes; between-group change estimates with 95% CIs; maintenance report used linear mixed models. | – | – | – |
| Rantula, O. A. 2025 | – | – | – | – | Automated PPG algorithm evaluated against 3-lead ambulatory ECG; sensitivity, specificity, PPV, NPV; ICC for AF burden; rhythm-change detection sensitivity and delay across window lengths. |
| Pu, J. 2025 | – | – | – | – | Prospective randomized two-group trial; conventional between-group comparisons of perioperative and wearable sleep parameters across pre-op, POD1 and POD3. |
| Holdom, C. J. 2025 | – | – | Longitudinal joint models of ALSFRS-R and accelerometry with overall survival; partial correlations; principal component analysis; clinical-trial sample-size estimation. | Longitudinal joint models of ALSFRS-R and accelerometry with overall survival; partial correlations; principal component analysis; clinical-trial sample-size estimation. | – |
| Stein, R. G. 2025 | – | Cross-sectional baseline analysis; multivariable regression models with PA×sleep interaction terms predicting ADAS-Cog-13, repeated in sex-stratified analyses. | – | Cross-sectional baseline analysis; multivariable regression models with PA×sleep interaction terms predicting ADAS-Cog-13, repeated in sex-stratified analyses. | – |
| Alnes, S. R. 2026 | – | Linear mixed models for repeated measures; group × time effects; effect sizes and 95% CIs; within-intervention wearable activity comparisons | – | Linear mixed models for repeated measures; group × time effects; effect sizes and 95% CIs; within-intervention wearable activity comparisons | – |
| Lachant, D. J. 2026 | – | Linear mixed-effects repeated-measures models for treatment×time interaction; adjusted least-squares means; linear regression relating change in stroke volume index to activity parameters; sensitivity analysis. | – | Linear mixed-effects repeated-measures models for treatment×time interaction; adjusted least-squares means; linear regression relating change in stroke volume index to activity parameters; sensitivity analysis. | – |
| Schäfer, L. 2026 | – | – | Prospective single-centre comparison of continuous patch vs routine infrared thermometry; fever detection rates and timing; correlations with laboratory markers and frequency of clinical interventions; clinical-relevance scoring system. | – | – |
| Werner, C. 2026 | – | – | – | Secondary analysis of single-centre double-blind placebo-controlled RCT; between-group longitudinal comparisons of DMOs at baseline, 12 weeks and 12-week follow-up. | – |
| Murray, R. M. 2025 | – | – | – | Two-arm randomized trial; longitudinal group comparisons of MVPA plus analyses of social support/partner MVPA associations with participant MVPA. | – |
| Mohapatra, S. 2025 | – | – | Three-arm 180-day RCT; adherence proportions compared across arms; correlation analyses between daily step count and disease-specific symptom severity. | – | – |
| Greysen, S. R. 2022 | – | Secondary analysis; multiple imputation (mice; CART for sleep); multivariable linear regression of 13-week functional outcomes by activity category adjusted for age, gender, Charlson comorbidity index and trial arm. | – | – | – |
| Li, G. 2025 | – | Generalized linear mixed-effects models assessing change from intervention initiation to 27 weeks; immediate-vs-delayed randomized comparison and delayed-group post-intervention analysis. | – | – | – |
| Dickerson, S. S. 2026 | – | – | – | – | ANCOVA adjusted for treatment, cancer type, interaction and baseline; exact permutation tests with 10,000 Monte Carlo simulations; effect sizes/95% CIs |
| Wang, H. 2025 | – | – | Multiscale fusion/adaptive-length ML PPG AF algorithm validated against 24-h Holter; sensitivity, specificity, precision, accuracy, F1, AUC, mean absolute error and Spearman correlation for five AF-burden metrics. | – | – |
| Blondeel, A. 2025 | – | Between-group longitudinal comparisons of change in objectively measured PA at 6 and 12 months; responder analysis; mixed-model framework reported for trial analyses | – | Between-group longitudinal comparisons of change in objectively measured PA at 6 and 12 months; responder analysis; mixed-model framework reported for trial analyses | – |
| van Vliet, M. 2025 | – | – | Prospective head-to-head validation vs 24-h Holter plus 27-day extended monitoring; AI PPG/ECG algorithms assessed with sensitivity, specificity and AF-burden correlation/agreement; compliance and AF prevalence summarized. | – | – |
| Zhu, B. 2025 | – | – | – | – | Random allocation to wireless vs conventional monitoring; between-group comparisons of turnaround time, total procedure/discharge time, monitoring time, hypotension/bradycardia/hypoxia, staff ratings and effective-monitoring rate. |
| Vetsch, T. 2025 | – | – | Secondary outcome analysis of prospective two-arm parallel RCT; between-group preoperative PA comparisons and comparison/correlation of steps with raw acceleration metrics. | – | – |
| Ahluwalia, N. 2026 | – | Cox proportional hazards model (intention-to-treat); Kaplan–Meier analysis; sensitivity analysis; between-group comparisons | Cox proportional hazards model (intention-to-treat); Kaplan–Meier analysis; sensitivity analysis; between-group comparisons | Cox proportional hazards model (intention-to-treat); Kaplan–Meier analysis; sensitivity analysis; between-group comparisons | – |
| Dong, Y. 2024 | – | – | – | – | Four-group randomized, double-blind trial; modified intention-to-treat analysis; between-group comparisons of POSD and Fitbit-derived postoperative sleep structure across postoperative days. |
| Flick, M. 2026 | – | – | Prospective descriptive observational analysis of percentage and cumulative monitoring time outside prespecified vital-sign thresholds; medians/IQRs and proportions with sustained abnormal episodes. | – | – |
| Küng, P. 2025 | – | – | – | Secondary 14-day intensive-longitudinal analysis; separate multilevel models for targets and agents linking daily persuasion/pressure to accelerometer PA and affect. | – |
| Vetrovsky, T. 2024 | – | – | – | – | Multicentre parallel-group RCT; primary intention-to-treat analysis; between-group 6MWT comparison and secondary ActiGraph step/MVPA outcomes over 6 months. |
| Docherty, K. F. 2024 | – | – | – | – | ANCOVA adjusted for baseline outcome, LVEF, trial, age, sex, BMI, NT-proBNP, NYHA class and geographic region; treatment-effect estimates and 95% CIs; no imputation |
| Wagnild, J. M. 2025 | – | – | – | Secondary analysis of UK Mini Mitral RCT with a new accelerometry primary endpoint; repeated follow-up at baseline and 6, 12, 18, 24, 38 and 52 weeks; between-arm mean differences with 95% CIs; blinded accelerometry processing. | – |
| Wheeler, A. P. 2025 | – | – | Prospective non-interventional descriptive study; physical activity, annualised bleeding rates, target joints and HJHS summarized by haemophilia type, inhibitor status and treatment strategy. | – | – |
| Kumar, A. 2026 | – | – | Pragmatic RCT; adjusted between-group mean differences for continuous outcomes and proportions achieving risk-factor targets; physical activity/step counts assessed as secondary outcomes. | – | – |
| Saito, T. 2025 | – | AF detection rate with 95% CI; chi-square for categorical and Mann–Whitney for continuous comparisons; univariable and multivariable logistic regression for determinants of AF detection; Kaplan–Meier cumulative incidence of new AF. | AF detection rate with 95% CI; chi-square for categorical and Mann–Whitney for continuous comparisons; univariable and multivariable logistic regression for determinants of AF detection; Kaplan–Meier cumulative incidence of new AF. | AF detection rate with 95% CI; chi-square for categorical and Mann–Whitney for continuous comparisons; univariable and multivariable logistic regression for determinants of AF detection; Kaplan–Meier cumulative incidence of new AF. | – |
| Shakya, S. 2026 | – | Cross-sectional baseline analysis; two-sample t tests; bivariate and multivariable logistic regression relating social isolation, movement behaviors and biomarkers to fatigue, adjusted for age, race/ethnicity, marital status and education. | Cross-sectional baseline analysis; two-sample t tests; bivariate and multivariable logistic regression relating social isolation, movement behaviors and biomarkers to fatigue, adjusted for age, race/ethnicity, marital status and education. | – | – |
| Alcoceba-Herrero, I. 2024 | – | Descriptive/bivariate comparisons; chi-square/Cramér’s V; multivariable logistic regression for severe alerts; AI algorithms automatically classified/managed monitoring alerts | Descriptive/bivariate comparisons; chi-square/Cramér’s V; multivariable logistic regression for severe alerts; AI algorithms automatically classified/managed monitoring alerts | – | – |
| Solk, P. 2025 | – | – | MOST full-factorial randomization to five intervention components; descriptive accrual/retention/adherence/acceptability; component-specific effects on MVPA goal adherence and acceptability. | – | – |
| Barua, S. 2025 | k-means time-series clustering (k=2 and k=8); multivariable linear regression relating adherence phenotypes to change in 6MWD | k-means time-series clustering (k=2 and k=8); multivariable linear regression relating adherence phenotypes to change in 6MWD | – | k-means time-series clustering (k=2 and k=8); multivariable linear regression relating adherence phenotypes to change in 6MWD | – |
| Bruna, C. L. 2026 | – | – | – | Randomized-group longitudinal comparisons of postoperative activity and HRV over 90 days; between-group differences with 95% CIs and P values across postoperative time points | – |
| Sun, V. 2025 | – | – | – | Randomized comparative-effectiveness trial of RPM care vs surgeon-only care; longitudinal functional recovery and symptom comparisons; postoperative complication comparisons; threshold-triggered monitoring workflow. | – |
| Rethorst, C. D. 2024 | – | Full-factorial MOST RCT; normal linear mixed models (SAS MIXED) with participant-specific random/separate intercepts to estimate main effects of four intervention components on MVPA and LPA at 3 and 6 months. | – | – | – |
| Abdelhamid, K. 2025 | – | – | Descriptive statistics; confidence intervals; p-values for timing/night-vs-day burden; compliance; PPG arrhythmia screening with confirmatory Holter ECG | – | – |
| Rees-Punia, E. 2025 | – | Trial-within-cohort randomized 2:1; intention-to-treat linear mixed models for MVPA and sedentary-time change at 3, 6 and 12 months; stratified analyses by website use and baseline inactivity. | – | – | – |
| van Steijn, N. J. 2026 | – | Prospective multicentre RCT; new-onset AF compared between groups using risk difference and Cox/time-to-event analysis (HR); 180-day remote monitoring; ECG review within 24 h. | – | – | – |
| Lim, J. S. 2025 | – | – | – | – | Modified intention-to-treat RCT analyses; group comparisons for cognitive endpoints; wrist-actigraphy MVPA adherence/duration comparisons; exploratory interaction/subgroup analyses linking physical activity with cognition/neuroimaging. |
| Chen, Y. 2025 | ML cough-feature models to predict lung function; multimodal COPD classifier combining cough and smartwatch physiology; MAE, Pearson correlation, Bland–Altman, accuracy, sensitivity and specificity | – | ML cough-feature models to predict lung function; multimodal COPD classifier combining cough and smartwatch physiology; MAE, Pearson correlation, Bland–Altman, accuracy, sensitivity and specificity | – | – |
| Kókai, L. L. 2025 | – | – | – | Three-arm randomized clinical trial; intention-to-treat longitudinal treatment-effect models across week 9, 21 and 61, including baseline-MVPA interaction analyses. | – |
| Lödding, P. 2024 | – | Prospective multicentre 1:1 RCT; longitudinal pre/post comparisons of PA and adherence; multivariable regression for predictors of change in exercise-related PA and completed exercise videos. | – | Prospective multicentre 1:1 RCT; longitudinal pre/post comparisons of PA and adherence; multivariable regression for predictors of change in exercise-related PA and completed exercise videos. | – |
| Engdal, M. 2024 | – | Pooled exploratory prospective cohort analysis of four prior RCT datasets; continuous 1-year trajectories estimated using quantile regression models with splines for median, 25th and 75th percentiles. | – | – | – |
| Zwiers, L. C. 2025 | – | – | – | – | Prospective single-blinded two-period two-sequence randomized crossover trial; experimental wearable+symptom algorithm vs symptom-only algorithm; sensitivity, specificity and timing of alerts relative to positive SARS-CoV-2 test. |
| Yarnall, A. J. 2026 | – | – | – | – | International clinical-validation cohort; PD vs matched-control and disease-stage comparisons across 24 DMOs; compliance/feasibility analyses. |
| Jones, T. 2026 | Intention-to-treat. Logistic regression for 12-month exacerbation status adjusted for condition, baseline age and gender; Poisson regression for exacerbation counts; Kaplan–Meier survival analysis with Wilcoxon test for time to first exacerbation; GLMMs with restricted maximum likelihood for secondary outcomes including device-measured MVPA, with condition, time and condition×time fixed effects and participant nested within condition as random effect; multiple imputation by chained equations/predictive mean matching (10 datasets). Accelerometer raw data processed using validated machine-learned random forest classifiers; 15-s non-overlapping windows; ENMO threshold for walking classification. | Intention-to-treat. Logistic regression for 12-month exacerbation status adjusted for condition, baseline age and gender; Poisson regression for exacerbation counts; Kaplan–Meier survival analysis with Wilcoxon test for time to first exacerbation; GLMMs with restricted maximum likelihood for secondary outcomes including device-measured MVPA, with condition, time and condition×time fixed effects and participant nested within condition as random effect; multiple imputation by chained equations/predictive mean matching (10 datasets). Accelerometer raw data processed using validated machine-learned random forest classifiers; 15-s non-overlapping windows; ENMO threshold for walking classification. | Intention-to-treat. Logistic regression for 12-month exacerbation status adjusted for condition, baseline age and gender; Poisson regression for exacerbation counts; Kaplan–Meier survival analysis with Wilcoxon test for time to first exacerbation; GLMMs with restricted maximum likelihood for secondary outcomes including device-measured MVPA, with condition, time and condition×time fixed effects and participant nested within condition as random effect; multiple imputation by chained equations/predictive mean matching (10 datasets). Accelerometer raw data processed using validated machine-learned random forest classifiers; 15-s non-overlapping windows; ENMO threshold for walking classification. | Intention-to-treat. Logistic regression for 12-month exacerbation status adjusted for condition, baseline age and gender; Poisson regression for exacerbation counts; Kaplan–Meier survival analysis with Wilcoxon test for time to first exacerbation; GLMMs with restricted maximum likelihood for secondary outcomes including device-measured MVPA, with condition, time and condition×time fixed effects and participant nested within condition as random effect; multiple imputation by chained equations/predictive mean matching (10 datasets). Accelerometer raw data processed using validated machine-learned random forest classifiers; 15-s non-overlapping windows; ENMO threshold for walking classification. | – |
| Khairat, S. 2026 | – | Secondary analysis of parent randomized pilot trial. Quantitative: descriptive frequencies/means; removal of missing/abnormal (negative) Fitbit data (18%); participant-level 7-day moving averages for daily steps and walking distance; intervention-vs-control t tests; linear regression of moving averages over time to compare temporal trends; significance threshold P<.05. Qualitative: inductive thematic analysis of 11 intervention exit interviews in Dedoose using a 25-code codebook developed by two coders; quantitative and qualitative findings integrated using an explanatory sequential mixed-methods design. | Secondary analysis of parent randomized pilot trial. Quantitative: descriptive frequencies/means; removal of missing/abnormal (negative) Fitbit data (18%); participant-level 7-day moving averages for daily steps and walking distance; intervention-vs-control t tests; linear regression of moving averages over time to compare temporal trends; significance threshold P<.05. Qualitative: inductive thematic analysis of 11 intervention exit interviews in Dedoose using a 25-code codebook developed by two coders; quantitative and qualitative findings integrated using an explanatory sequential mixed-methods design. | Secondary analysis of parent randomized pilot trial. Quantitative: descriptive frequencies/means; removal of missing/abnormal (negative) Fitbit data (18%); participant-level 7-day moving averages for daily steps and walking distance; intervention-vs-control t tests; linear regression of moving averages over time to compare temporal trends; significance threshold P<.05. Qualitative: inductive thematic analysis of 11 intervention exit interviews in Dedoose using a 25-code codebook developed by two coders; quantitative and qualitative findings integrated using an explanatory sequential mixed-methods design. | – |
| Khetrapal, P. 2024 | – | Secondary observational analysis specified in the iROC protocol. Included participants with ≥3 days of data and ≥100 steps/day. Chi-square for categorical comparisons; Student t test or ANOVA for continuous variables; Kaplan–Meier plots with log-rank tests for time-to-event data; Pearson correlation for continuous associations; linear regression for relationships between activity and length of stay or days alive out of hospital within 90 days, with separate models incorporating participant demographics and baseline/postoperative activity, chair-to-stand or EQ5D self-assessed health. Two-sided alpha 0.05; SPSS v29 and GraphPad Prism v9. | Secondary observational analysis specified in the iROC protocol. Included participants with ≥3 days of data and ≥100 steps/day. Chi-square for categorical comparisons; Student t test or ANOVA for continuous variables; Kaplan–Meier plots with log-rank tests for time-to-event data; Pearson correlation for continuous associations; linear regression for relationships between activity and length of stay or days alive out of hospital within 90 days, with separate models incorporating participant demographics and baseline/postoperative activity, chair-to-stand or EQ5D self-assessed health. Two-sided alpha 0.05; SPSS v29 and GraphPad Prism v9. | Secondary observational analysis specified in the iROC protocol. Included participants with ≥3 days of data and ≥100 steps/day. Chi-square for categorical comparisons; Student t test or ANOVA for continuous variables; Kaplan–Meier plots with log-rank tests for time-to-event data; Pearson correlation for continuous associations; linear regression for relationships between activity and length of stay or days alive out of hospital within 90 days, with separate models incorporating participant demographics and baseline/postoperative activity, chair-to-stand or EQ5D self-assessed health. Two-sided alpha 0.05; SPSS v29 and GraphPad Prism v9. | – |
| Kosik-Rose, E. 2026 | – | Baseline-only secondary analysis of TAK-861-2001 phase 2 RCT; no treatment-arm or dose data analysed. Epoch-level pulse-rate preprocessing excluded pulse rate 0/missing, device wear <50%, and physiologically implausible pulse-rate/activity combinations. Numerical experiments used two-stage bootstrap (500 repetitions) across simulated invalid-data levels to evaluate RMSE, MAE, mean error, error SD, Pearson r, concordance correlation coefficient and ICC, leading to a ≥70% valid-minute threshold per day. Population means and pulse-rate–motor-activity associations were estimated with linear mixed-effects models in R 4.3.1 with participant random intercepts; association models also included random slopes. Sensitivity analyses excluded high nighttime motor-activity values above the 95th and 80th percentiles. Fixed-effect p values used t tests with Satterthwaite degrees of freedom. | Baseline-only secondary analysis of TAK-861-2001 phase 2 RCT; no treatment-arm or dose data analysed. Epoch-level pulse-rate preprocessing excluded pulse rate 0/missing, device wear <50%, and physiologically implausible pulse-rate/activity combinations. Numerical experiments used two-stage bootstrap (500 repetitions) across simulated invalid-data levels to evaluate RMSE, MAE, mean error, error SD, Pearson r, concordance correlation coefficient and ICC, leading to a ≥70% valid-minute threshold per day. Population means and pulse-rate–motor-activity associations were estimated with linear mixed-effects models in R 4.3.1 with participant random intercepts; association models also included random slopes. Sensitivity analyses excluded high nighttime motor-activity values above the 95th and 80th percentiles. Fixed-effect p values used t tests with Satterthwaite degrees of freedom. | – | – |
| Krell, V. 2024 | – | Two-arm non-randomized controlled intervention with participant-preference allocation (TE n=61; rehabilitation-sports control n=31), 6-month intervention plus 6-month follow-up. Baseline characteristics descriptive. Primary VO2peak non-inferiority analysis used adjusted linear regression with 95% CIs and predefined margin −1.50 ml/min/kg; adjustment variables selected using a DAG. Secondary endpoints were descriptive with adjusted linear-regression non-inferiority analyses; no multiplicity adjustment. Primary-endpoint missing data (13.9%) handled by predictive-mean-matching multiple imputation (10 imputations; mice in R 4.3.1). Fitbit valid day ≥10 h wear; valid week ≥4 valid days; wearable table included participants with ≥50% valid weeks. Steps reported mean/valid day and active minutes mean weekly sum. | Two-arm non-randomized controlled intervention with participant-preference allocation (TE n=61; rehabilitation-sports control n=31), 6-month intervention plus 6-month follow-up. Baseline characteristics descriptive. Primary VO2peak non-inferiority analysis used adjusted linear regression with 95% CIs and predefined margin −1.50 ml/min/kg; adjustment variables selected using a DAG. Secondary endpoints were descriptive with adjusted linear-regression non-inferiority analyses; no multiplicity adjustment. Primary-endpoint missing data (13.9%) handled by predictive-mean-matching multiple imputation (10 imputations; mice in R 4.3.1). Fitbit valid day ≥10 h wear; valid week ≥4 valid days; wearable table included participants with ≥50% valid weeks. Steps reported mean/valid day and active minutes mean weekly sum. | Two-arm non-randomized controlled intervention with participant-preference allocation (TE n=61; rehabilitation-sports control n=31), 6-month intervention plus 6-month follow-up. Baseline characteristics descriptive. Primary VO2peak non-inferiority analysis used adjusted linear regression with 95% CIs and predefined margin −1.50 ml/min/kg; adjustment variables selected using a DAG. Secondary endpoints were descriptive with adjusted linear-regression non-inferiority analyses; no multiplicity adjustment. Primary-endpoint missing data (13.9%) handled by predictive-mean-matching multiple imputation (10 imputations; mice in R 4.3.1). Fitbit valid day ≥10 h wear; valid week ≥4 valid days; wearable table included participants with ≥50% valid weeks. Steps reported mean/valid day and active minutes mean weekly sum. | – |
| Lee, J. 2024 | – | Intention-to-treat analyses. Between-group outcomes at individual time points were compared using linear regression; changes across baseline, 2 weeks and 6 months used linear mixed-effects models with visit and visit×group interaction and participant random intercepts. Sensitivity analysis used propensity-score matching: logistic regression estimated treatment probability using covariates including season and baseline social function, followed by 1:1 nearest-neighbor matching with caliper 0.2. Two-sided P<0.05; Stata 16. | – | – | – |
| Leps, C. 2025 | – | Observational analysis pooling both original CRBP-TS randomized groups because physical activity did not differ between groups. Multiple linear regression models used wearable MVPA or steps as exposures and adjusted for age, gender, BMI (except body-composition models) and baseline value of the dependent variable. Extreme outliers (>3×IQR) were excluded before modelling; residual assumptions, linearity, homoscedasticity and multicollinearity were checked. Results reported with adjusted R², unstandardized beta coefficients, 95% CIs and p values. IBM SPSS Statistics v29. | – | – | – |
| Li, G. 2025 | – | Secondary longitudinal observational analysis of data from a five-arm pilot RCT. Parent trial randomized 110 participants, but this report selected the 77 completers and did not compare randomized intervention groups; all participants had received the weekly reports. Descriptive statistics; multiple imputation for missing data; mixed-effects models with participant-level random effects for 84-day trajectories and effects of weekly reports on steps, sleep and symptoms, including report × time interaction; lagged regression using prior-day steps (scaled per 5,000 steps) and sleep (per 2 h) to predict next-day symptoms; moderation analyses with interaction terms for baseline characteristics. SPSS 25 and Stata 15. | Secondary longitudinal observational analysis of data from a five-arm pilot RCT. Parent trial randomized 110 participants, but this report selected the 77 completers and did not compare randomized intervention groups; all participants had received the weekly reports. Descriptive statistics; multiple imputation for missing data; mixed-effects models with participant-level random effects for 84-day trajectories and effects of weekly reports on steps, sleep and symptoms, including report × time interaction; lagged regression using prior-day steps (scaled per 5,000 steps) and sleep (per 2 h) to predict next-day symptoms; moderation analyses with interaction terms for baseline characteristics. SPSS 25 and Stata 15. | Secondary longitudinal observational analysis of data from a five-arm pilot RCT. Parent trial randomized 110 participants, but this report selected the 77 completers and did not compare randomized intervention groups; all participants had received the weekly reports. Descriptive statistics; multiple imputation for missing data; mixed-effects models with participant-level random effects for 84-day trajectories and effects of weekly reports on steps, sleep and symptoms, including report × time interaction; lagged regression using prior-day steps (scaled per 5,000 steps) and sleep (per 2 h) to predict next-day symptoms; moderation analyses with interaction terms for baseline characteristics. SPSS 25 and Stata 15. | – |
| Liu, C. 2026 | – | Single-blind quasi-experimental matched-group study; allocation by registration order/scheduling rather than randomization. Experimental group received 10 weekly 130-min BMS sessions; comparison group usual care. Primary analyses used intention-to-treat. Descriptive statistics; chi-square tests for categorical variables; Mann–Whitney U tests for baseline continuous variables; generalized estimating equations for group, time and group×time effects, adjusting for sex and VI severity and, for sleep outcomes, average daytime physical activity; incomplete repeated measures handled under missing-at-random without imputation; Cohen's d for between-group change effect sizes. SPSS v24. | Single-blind quasi-experimental matched-group study; allocation by registration order/scheduling rather than randomization. Experimental group received 10 weekly 130-min BMS sessions; comparison group usual care. Primary analyses used intention-to-treat. Descriptive statistics; chi-square tests for categorical variables; Mann–Whitney U tests for baseline continuous variables; generalized estimating equations for group, time and group×time effects, adjusting for sex and VI severity and, for sleep outcomes, average daytime physical activity; incomplete repeated measures handled under missing-at-random without imputation; Cohen's d for between-group change effect sizes. SPSS v24. | Single-blind quasi-experimental matched-group study; allocation by registration order/scheduling rather than randomization. Experimental group received 10 weekly 130-min BMS sessions; comparison group usual care. Primary analyses used intention-to-treat. Descriptive statistics; chi-square tests for categorical variables; Mann–Whitney U tests for baseline continuous variables; generalized estimating equations for group, time and group×time effects, adjusting for sex and VI severity and, for sleep outcomes, average daytime physical activity; incomplete repeated measures handled under missing-at-random without imputation; Cohen's d for between-group change effect sizes. SPSS v24. | – |
| Liu, D. 2024 | – | 1:1 randomized, double-blind, placebo-controlled parallel-group pilot effectiveness RCT. Primary modified intention-to-treat analysis excluded 3 randomized participants with no study/sleep data; no imputation. Continuous outcomes assessed for normality and compared with Student's t tests or Mann–Whitney tests; categorical outcomes with Fisher's exact tests. Primary total sleep time was additionally adjusted for the two randomization stratification variables using linear regression. Sensitivity 'treatment received as intended' analysis excluded four participants who received study medication for <2 h. Two-sided alpha 0.05; no multiple-comparison adjustment, so prespecified secondary outcomes were considered exploratory. Stata IC 17. | 1:1 randomized, double-blind, placebo-controlled parallel-group pilot effectiveness RCT. Primary modified intention-to-treat analysis excluded 3 randomized participants with no study/sleep data; no imputation. Continuous outcomes assessed for normality and compared with Student's t tests or Mann–Whitney tests; categorical outcomes with Fisher's exact tests. Primary total sleep time was additionally adjusted for the two randomization stratification variables using linear regression. Sensitivity 'treatment received as intended' analysis excluded four participants who received study medication for <2 h. Two-sided alpha 0.05; no multiple-comparison adjustment, so prespecified secondary outcomes were considered exploratory. Stata IC 17. | – | – |
| Mura, M. 2025 | – | 1:1 randomized controlled single-centre trial; intention-to-treat analyses. Baseline categorical variables: Pearson chi-squared; continuous variables: Student t test or Wilcoxon after distribution inspection. Outcomes analysed with linear mixed-effects models with participant random intercept and fixed effects for time, arm, and time×arm interaction; planned contrasts with emmeans. Additional multivariable linear regression adjusted for sex, age, diabetes, smoking status, and baseline value. Binary IPH presence analysed with generalized linear mixed-effects model using binomial distribution/logit link. R 4.1.2. | 1:1 randomized controlled single-centre trial; intention-to-treat analyses. Baseline categorical variables: Pearson chi-squared; continuous variables: Student t test or Wilcoxon after distribution inspection. Outcomes analysed with linear mixed-effects models with participant random intercept and fixed effects for time, arm, and time×arm interaction; planned contrasts with emmeans. Additional multivariable linear regression adjusted for sex, age, diabetes, smoking status, and baseline value. Binary IPH presence analysed with generalized linear mixed-effects model using binomial distribution/logit link. R 4.1.2. | – | – |
| Page, L. L. 2024 | – | – | Device-agreement analysis using Polar H10 as criterion. Lin concordance correlation coefficient (rc) with 95% CIs assessed agreement; Bland–Altman plots visualized agreement; absolute paired differences and relative error rates were calculated at each exercise stage. Data analysed in SPSS 27 and RStudio 1.3.1093. Analysis included 200 supervised exercise sessions from 14 participants. | – | – |
| Ptomey, L. T. 2026 | – | Primary analysis used two-level hierarchical linear mixed-effects models for repeated MVPA measurements nested within participants, testing arm, time (linear/nonlinear) and arm×time interaction and adjusting for age and sex. Missing outcomes were handled with model-based imputation after assessment of missing-at-random assumptions. ANCOVA compared 6-month MVPA adjusting for baseline MVPA, age and sex. MVPA was log-transformed for right skew. Sedentary time used analogous linear mixed models; functional fitness used ANCOVA. Valid ActiGraph data required ≥10 h/day on ≥3 days including 1 weekend day. Activity intensity used Aguilar-Farías sedentary and Freedson vector-magnitude cut points. | – | Primary analysis used two-level hierarchical linear mixed-effects models for repeated MVPA measurements nested within participants, testing arm, time (linear/nonlinear) and arm×time interaction and adjusting for age and sex. Missing outcomes were handled with model-based imputation after assessment of missing-at-random assumptions. ANCOVA compared 6-month MVPA adjusting for baseline MVPA, age and sex. MVPA was log-transformed for right skew. Sedentary time used analogous linear mixed models; functional fitness used ANCOVA. Valid ActiGraph data required ≥10 h/day on ≥3 days including 1 weekend day. Activity intensity used Aguilar-Farías sedentary and Freedson vector-magnitude cut points. | – |
| Torres-Lopez, L. V. 2024 | – | Chronic sleep effects were analysed primarily per protocol using ANCOVA: postintervention sleep outcome as dependent variable, randomized group as fixed factor, and baseline outcome as covariate; raw and standardized z-score effects were reported. Exploratory ITT analyses included all 109 randomized participants and used predictive mean-matching multiple imputation after checking missing-at-random assumptions. Acute subsequent-night effects within the exercise arm used linear mixed regression models with exercise-session attendance as fixed factor, participant ID as random factor, and sleep indicators as outcomes. A one-dimensional statistical parametric mapping (SPM1D) analysis compared 24-hour acceleration curves on attendance versus nonattendance days, with Student t tests identifying significant curve differences. | Chronic sleep effects were analysed primarily per protocol using ANCOVA: postintervention sleep outcome as dependent variable, randomized group as fixed factor, and baseline outcome as covariate; raw and standardized z-score effects were reported. Exploratory ITT analyses included all 109 randomized participants and used predictive mean-matching multiple imputation after checking missing-at-random assumptions. Acute subsequent-night effects within the exercise arm used linear mixed regression models with exercise-session attendance as fixed factor, participant ID as random factor, and sleep indicators as outcomes. A one-dimensional statistical parametric mapping (SPM1D) analysis compared 24-hour acceleration curves on attendance versus nonattendance days, with Student t tests identifying significant curve differences. | – | – |
| Zimniak, L. 2026 | – | Primary between-group comparisons used Mann–Whitney U tests for continuous outcomes and Fisher's exact tests for categorical outcomes, with p<0.05. The primary endpoint was cumulative wearable-recorded steps over POD 0–5. Prespecified exploratory subgroup analyses used negative binomial regression with intervention×surgical-approach interaction terms, adjusted for age, sex, BMI and smoking; incidence-rate ratios with 95% CIs were reported and interaction p-values derived from Wald tests. | Primary between-group comparisons used Mann–Whitney U tests for continuous outcomes and Fisher's exact tests for categorical outcomes, with p<0.05. The primary endpoint was cumulative wearable-recorded steps over POD 0–5. Prespecified exploratory subgroup analyses used negative binomial regression with intervention×surgical-approach interaction terms, adjusted for age, sex, BMI and smoking; incidence-rate ratios with 95% CIs were reported and interaction p-values derived from Wald tests. | – | – |
| Rosenfeldt, A. B. 2026 | – | Daily step counts were preprocessed using ≥500 steps/day as a valid wear-day threshold; participants with <175 valid days were excluded, and the lowest 2% of remaining daily observations by weekday/calendar month were removed to address heteroscedasticity. Step counts were log-transformed. Randomized AE versus UCC differences were analysed with linear mixed-effects regression with participant random intercepts and fixed effects for site and treatment. Longitudinal trends used random intercept-and-slope LMMs with weekday, site and interaction; natural cubic splines for maximum temperature and days since follow-up; and treatment×time interaction. AE exercise versus nonexercise days were modelled similarly. Models used restricted maximum likelihood; marginal means were back-transformed to median steps with 95% CIs using the delta method. Treatment effects were tested with Wald statistics using Kenward–Roger denominator degrees of freedom. | – | Daily step counts were preprocessed using ≥500 steps/day as a valid wear-day threshold; participants with <175 valid days were excluded, and the lowest 2% of remaining daily observations by weekday/calendar month were removed to address heteroscedasticity. Step counts were log-transformed. Randomized AE versus UCC differences were analysed with linear mixed-effects regression with participant random intercepts and fixed effects for site and treatment. Longitudinal trends used random intercept-and-slope LMMs with weekday, site and interaction; natural cubic splines for maximum temperature and days since follow-up; and treatment×time interaction. AE exercise versus nonexercise days were modelled similarly. Models used restricted maximum likelihood; marginal means were back-transformed to median steps with 95% CIs using the delta method. Treatment effects were tested with Wald statistics using Kenward–Roger denominator degrees of freedom. | – |
| Seifert, M. 2024 | – | – | Intention-to-treat primary analysis; per-protocol analysis excluded participants who did not transmit step counts. Distribution assessed using histograms. Between-group continuous comparisons used Student t test or Mann–Whitney U; categorical comparisons Fisher exact or chi-square; repeated dependent physical-activity measures used Friedman test. Multivariable analysis for the primary composite endpoint included variables with univariate P<0.10 (age, sex, eGFR, BMI, and change in step count from baseline to 12 months). Time-to-event analysis used log-rank cumulative event rates from 90 days after PVI. Two-sided alpha 0.05; results reported as mean(SD), median(IQR), or relative risk with 95% CI; SPSS v27. | Intention-to-treat primary analysis; per-protocol analysis excluded participants who did not transmit step counts. Distribution assessed using histograms. Between-group continuous comparisons used Student t test or Mann–Whitney U; categorical comparisons Fisher exact or chi-square; repeated dependent physical-activity measures used Friedman test. Multivariable analysis for the primary composite endpoint included variables with univariate P<0.10 (age, sex, eGFR, BMI, and change in step count from baseline to 12 months). Time-to-event analysis used log-rank cumulative event rates from 90 days after PVI. Two-sided alpha 0.05; results reported as mean(SD), median(IQR), or relative risk with 95% CI; SPSS v27. | – |
| Vetrovsky, T. 2026 | – | – | – | 12-month follow-up of randomized WATCHFUL trial assessing persistence of between-group device-measured PA differences after the 6-month walking intervention. | – |

**Data Extraction Template**

**Study Identification**

- Study ID:
- Study No.:
- Title:
- Continent:
- Country:

**Study Overview**

- Aim of study:
- Results:
- Study Design:
- Study setting:
- Disease/Condition:

**Participant Characteristics**

- Number of participants:
- Age range:
- Mean age:
- Median age:
- Gender distribution:
- Ethnic distribution:

**Wearable Device Information**

- Wearable device:
- Sensor:
- Type of device:
- Application of wearable:
- Placement on body:
- Duration of study:
- Duration of wear:
- Ease of use:

**Outcome Measures**

**Activity Data**

- Primary Endpoint:
- Secondary Endpoint:
- Disease monitoring:
- Other outcomes:

**Heart Rate**

- Primary Endpoint:
- Secondary Endpoint:
- Disease monitoring:
- Other outcomes:

**Blood Oxygen**

- Primary Endpoint:
- Secondary Endpoint:
- Disease characterisation:
- Other outcomes:

**Sleep**

- Primary Endpoint:
- Secondary Endpoint:
- Disease monitoring:
- Other outcomes:

**Respiratory Rate**

- Primary Endpoint:
- Secondary Endpoint:
- Disease characterisation:
- Other outcomes:

**Skin Temperature**

- Primary Endpoint:
- Secondary Endpoint:
- Disease characterisation:
- Other outcomes:

**ECG**

- Primary Endpoint:
- Secondary Endpoint:
- Disease characterisation:
- Other outcomes:

**Blood Pressure**

- Primary Endpoint:
- Secondary Endpoint:
- Disease characterisation:
- Other outcomes:

**Electrodermal Activity**

- Primary Endpoint:
- Secondary Endpoint:
- Disease characterisation:
- Other outcomes:

**Expiratory Variability Index**

- Primary Endpoint:
- Secondary Endpoint:
- Disease characterisation:
- Other outcomes:

**Data Analysis Methods**

**General**

- Data Analysis (summary):

**Statistical & Analytical Methods**

- Artificial Intelligence methods:
- Regression Analysis:
- t-test Analysis:
- Correlation Analysis:
- Non-parametric Analysis:
- Descriptive Statistics:
- Mixed Methods Models:
- Chi-square / Fisher Yates tests:
- False Discovery Rate:
- Inferential Statistics:
- Time Series Analysis:
- ARMA Models:
- Survival Analysis (time-to-event):
- Multivariate Analysis:
- Bayesian Analysis:
- ANOVA:
- Clustering methods:
- Cross-validation:
