# Supplementary material for: Scoping review of wearable device data evaluated in clinical studies
Source: BMJ Open. 2026 Nov 2;16(11):e111027. doi: 10.1136/bmjopen-2025-111027 (PMC13735340; doi:10.1136/bmjopen-2025-111027)
Supplement: Supplementary file 5 [file bmjopen-16-11-s004.docx]

**Wearable data collected from clinical study participants and its application**

| **Clinical Study**  **Application** |  | **Physiological and Behavioural Measure N (%)** | | | | | | | | |
| --- | --- | --- | --- | --- | --- | --- | --- | --- | --- | --- |
|  | **Overall**  **N (%)** | **Activity** | **Heart Rate** | **Heart Rate Variability** | **SpO_2_** | **Sleep** | **RR** | **Skin Temp** | **ECG** | **BP** |
| **Primary Endpoint** | 52 (35) | 37 (25) | 14 (10) | 3 (2) | 3 (2) | 9 (6) | 3 (2) | 4 (3) | 5 (3) | 3 (2) |
| **Secondary Endpoint** | 46 (31) | 37 (25) | 17 (12) | 4 (3) | 3 (2) | 12 (8) | 2 (1) | 0 | 2 (1) | 2 (1) |
| **Disease monitoring** | 49 (33) | 33 (22) | 17 (12) | 5 (3) | 5 (3) | 13 (9) | 5 (3) | 5 (3) | 8 (5) | 1 (1) |
| **Total** | 147 (100) | 106 (72) | 48 (33) | 12 (8) | 11 (7) | 34 (23) | 10 (7) | 9 (6) | 15 (10) | 6 (4) |

**Table Legend**: **Activity**: Includes step count, physical activity, and step distance. **Heart Rate**: Includes Heart Rate, Resting Heart Rate, Mean Heart Rate, Median Heart Rate, RR intervals, Atrial Fibrillation , and irregular rhythm. **Heart Rate Variability:** has been separated from heart rate. SpO_2_: Blood oxygen  **RR**: Respiratory Rate. **ECG**: Electrocardiogram. **BP**: Blood Pressure. Data are presented as n (%); percentages are calculated using the total number of included studies (N=147) and rounded to the nearest whole number. Physiological and behavioural measures are not mutually exclusive.
